# Modeling spatial variation in density of golden eagle nest sites in the western United States

**DOI:** 10.1371/journal.pone.0223143

**Published:** 2019-09-30

**Authors:** Jeffrey R. Dunk, Brian Woodbridge, Todd M. Lickfett, Geoffrey Bedrosian, Barry R. Noon, David W. LaPlante, Jessi L. Brown, Jason D. Tack

**Affiliations:** 1 Department of Environmental Science and Management, Humboldt State University, Arcata, CA, United States of America; 2 U.S. Fish and Wildlife Service, Corvallis, Oregon, United States of America; 3 U.S. Fish and Wildlife Service, Denver Federal Center, Denver, Colorado, United States of America; 4 Department of Fish, Wildlife, and Conservation Biology and Graduate Degree Program in Ecology, Colorado State University, Fort Collins, CO, United States of America; 5 Natural Resource Geospatial, Montague, CA, United States of America; 6 Department of Biology, University of Nevada Reno, Reno, NV, United States of America; 7 U.S. Fish and Wildlife Service, Missoula, Montana, United States of America; Peking University, CHINA

## Abstract

In order to contribute to conservation planning efforts for golden eagles (*Aquila chrysaetos*) in the western U.S., we developed nest site models using >6,500 nest site locations throughout a >3,483,000 km^2^ area of the western U.S. We developed models for twelve discrete modeling regions, and estimated relative density of nest sites for each region. Cross-validation showed that, in general, models accurately estimated relative nest site densities within regions and sub-regions. Areas estimated to have the highest densities of breeding golden eagles had from 132–2,660 times greater densities compared to the lowest density areas. Observed nest site densities were very similar to those reported from published studies. Large extents of each modeling region consisted of low predicted nest site density, while a small percentage of each modeling region contained disproportionately high nest site density. For example, we estimated that areas with relative nest density values <0.3 represented from 62.8–97.8% ($\bar{x}$ = 82.5%) of each modeling area, and those areas contained from 14.7–30.0% ($\bar{x}$ = 22.1%) of the nest sites. In contrast, areas with relative nest density values >0.5 represented from 1.0–12.8% ($\bar{x}$ = 6.3%) of modeling areas, and those areas contained from 47.7–66.9% ($\bar{x}$ = 57.3%) of the nest sites. Our findings have direct application to: 1) large-scale conservation planning efforts, 2) risk analyses for land-use proposals such as recreational trails or wind power development, and 3) identifying mitigation areas to offset the impacts of human disturbance.

## Introduction

The information needed to support effective conservation planning includes an understanding of how a species’ distribution, abundance, and demography vary with spatial heterogeneity in natural and anthropogenic features of its environment. At one end of the information spectrum, the distinction between a species’ area of occupancy (where it actually occurs within its geographic range) and its extent of occurrence (its geographic range; [1]) could identify areas where conservation efforts should be focused. Toward the other extreme, understanding how a species’ demography varies with habitat heterogeneity greatly aids in the identification of important areas and habitats for conservation, and enables modeling of the consequences of conservation actions (or inaction; e.g., [2, 3]). For many species of concern, however, demographic information (e.g., birth and survival rates) is not available and conservation planning must rely on indirect proxies of fitness including occurrence and abundance. Habitat selection theory suggests that territorial species should conform to the ideal despotic distribution (IDD; [4]), under which individuals select the highest suitability area that is currently unoccupied, and that the resulting distribution of territorial individuals is one where the highest suitability areas have higher densities of individuals and lower suitability areas have lower densities. Experimental and empirical evidence have generally supported the IDD theory [5, 6], including studies on raptors that have found an inverse relationship between territory size and prey abundance [7–10]. Thus, in the absence of data on how fitness varies with habitat for territorial species, variation in the density of territorial individuals can be interpreted as a proxy for spatial variation in habitat quality.

Using density as a proxy for habitat quality may be especially valuable in conservation planning for species with broad geographic ranges encompassing heterogeneous environments. For such species, it is unusual to have demographic information from multiple populations occupying different ecological settings (see [11] for an exception). This circumstance necessitates analyses and modeling with more readily available data on species occurrence and the relationships between location data and environmental covariates. To be useful for management and conservation, the resulting models must explicitly incorporate regional variation in habitat suitability.

These challenges are exemplified by conservation planning efforts for golden eagles (*Aquila chrysaetos*) whose breeding populations in western North America span diverse ecosystems from coastal woodlands and temperate forests to sagebrush steppe and southwestern deserts [12]. Golden eagles are large-bodied apex predators that occupy large but variable breeding home ranges (10s - >3,000 km^2^; [13]), and territories tend to be sparsely distributed at landscape scales. However, territory density can be relatively high (>6 territories/100 km^2^ [14]) in localized areas with abundant nest substrates and prey populations. Breeding territories typically contain multiple nests and support long-term occupancy across multiple generations of golden eagles [15].

In contrast to the relative stability of breeding distributions, golden eagles exhibit a wide variety of intra- and inter-annual movement patterns throughout the annual cycle. For example, populations occupying northern latitudes migrate 1,000s of km between breeding and wintering areas [16, 17], while more southerly populations tend to be resident [12]. Even within resident populations, sub-adult golden eagles (2–4 years of age) may exhibit wide variation in dispersal distances ranging from 10s - >1,000 km [18]. The combination of resident, migratory and dispersing individuals may result in high seasonal variation in the density and composition of golden eagles occupying a given landscape.

As a consequence of their protected status under the Bald and Golden Eagle Protection Act (Eagle Act; 16 U.S.C 668-668d) and potential and actual threats to their populations, golden eagles have received substantial attention from both regulatory agencies [19] and researchers [20]. Golden eagle populations in the western U.S. are stable or slightly decreasing [19, 20], but there is growing concern that increased energy development and land-use change may result in significant future population declines. Threats such as lead poisoning, electrocution, habitat modification/loss, energy development (especially wind energy), and outdoor recreation have long been known or suspected to impact golden eagle populations [19, 21–26]. However, the extent to which these factors, individually or in concert, influence overall golden eagle populations is uncertain. Strategies for managing or reducing such threats require both spatial data on the distribution of hazards (e.g., electrocution risk or lead poisoning) as well as spatial data on the distribution and abundance of golden eagles [27].

Due to the extensive areas required for persistence of golden eagle populations, and myriad threats to their populations, conservation planning for eagles requires planning at broad spatial scales. Population management objectives codified in regulations implementing the Eagle Act require management actions to be “consistent with the goals of maintaining stable or increasing breeding populations in all golden eagle management units and the persistence of local populations throughout the geographic range of each species” (Eagle Permits; Revisions to Regulations for Eagle Incidental Take and Take of Eagle Nests, 81 C.F.R Sect. 91494). A key feature of maintaining or increasing any population is protection of breeding areas. Thus, motivated by the Eagle Act’s requirement to maintain local and regional populations, we modeled the relative density of golden eagle nest sites (hereafter, relative nest site density [RND]) at the scale of large ecological regions (range = 87,288–711,384 km^2^) to account for broad-scale spatial heterogeneity in habitat relationships in the western U.S.

Data describing golden eagle breeding sites in the western U.S. are routinely collected by land management agencies, researchers, and energy developers during the course of project planning. These data are often collected opportunistically (i.e., without a formal sampling design; but see [28, 29]), limiting their application in presence-absence analysis designs. Presence-only (or presence-available) species distribution models (SDMs), however, have become powerful and popular tools for evaluating species-environment relationships (e.g., [30]). Presence-only models allow for analyses of opportunistically collected data such as museum records, citizen science observations, and monitoring data collected by land management agencies. Importantly, absence data are generally not recorded during these surveys. Conservation planning entails making decisions in the context of incomplete information [31], and presence-only models can provide insights into species-environment relationships that are more informative than location data alone [32–34]. However, the utility of a model based on uncertain or incomplete data depends on its validity and an understanding of its limitations.

MaxEnt [35] is one of the most popular software packages for modeling presence-only data, having been cited >10,000 times (Google Scholar, https://scholar.google.com, accessed 23 Aug 2019). The MaxEnt model algorithm, based on presence-available data, performs identically to fitting an inhomogeneous Poisson point process model [36–39]. As a result, model output can be interpreted as a measure of relative density [40] of the modeled events (e.g., individuals, den sites, rest sites, nest sites), and estimates provided from such models are proportional to event density [41]. Because it is referenced to area, relative density is a more accurate description of the modeled events. Using some simple transformations developed by Boyce et al. [42], we show how MaxEnt output can be used to estimate the magnitude of differences in density of modeled events among areas even when absolute density is unknown. These transformations provide a clear ecological interpretation of MaxEnt’s output, and improve the utility of predictions for conservation and management applications.

SDMs are often used to identify locations within the modeled landscape that have particularly high suitability or likelihood of occurrence values. Such areas may be subsequently prioritized for protection or other conservation action [3, 43, 44]. Similarly, SDMs can also be used to identify landscape locations that have low values where conservation is not prioritized and where development may be incentivized [45]. For species of conservation concern, often a relatively small percentage of the landscape within the species’ range contains the majority of the individuals/nests/dens in that landscape [3, 46, 47]. In such cases, relatively modest conservation actions (e.g., restrictions on allowable land uses) targeted to these areas may result in very large conservation benefits (e.g., [27]).

We developed and evaluated RND models to serve as a foundation for golden eagle conservation planning in the western U.S. Our findings have a number of direct applications relevant to golden eagle conservation including land management planning by federal, state and tribal agencies, evaluation of land-use proposals by regulatory agencies, and decision support for energy developers. Model results can support quantitative risk assessments for a variety of potential risks, including energy development (both renewable and conventional; [45]), electrocution [27], wildland fire, lead poisoning, andvarious land management decisions. Our research goals were to develop models that: 1) could accurately predict the relative density of golden eagle nest sites; 2) were well-calibrated (i.e., the prediction of RND is accurately scaled to observed nest density rather than “simply” discriminating nest sites from non-nest sites); 3) were scaled to reflect the spatial scale at which golden eagles respond to variation in habitat features; 4) could assess habitat use patterns at spatial scales relevant to both potential threats and management actions; 5) were robust (i.e., not over- or under-fit to the data that are used to train the models) and generalizable to the extent of the modeled landscape; and 6) could be used to inform a wide variety of management/conservation actions.

## Materials and methods

### Study area

**Fig 1:**
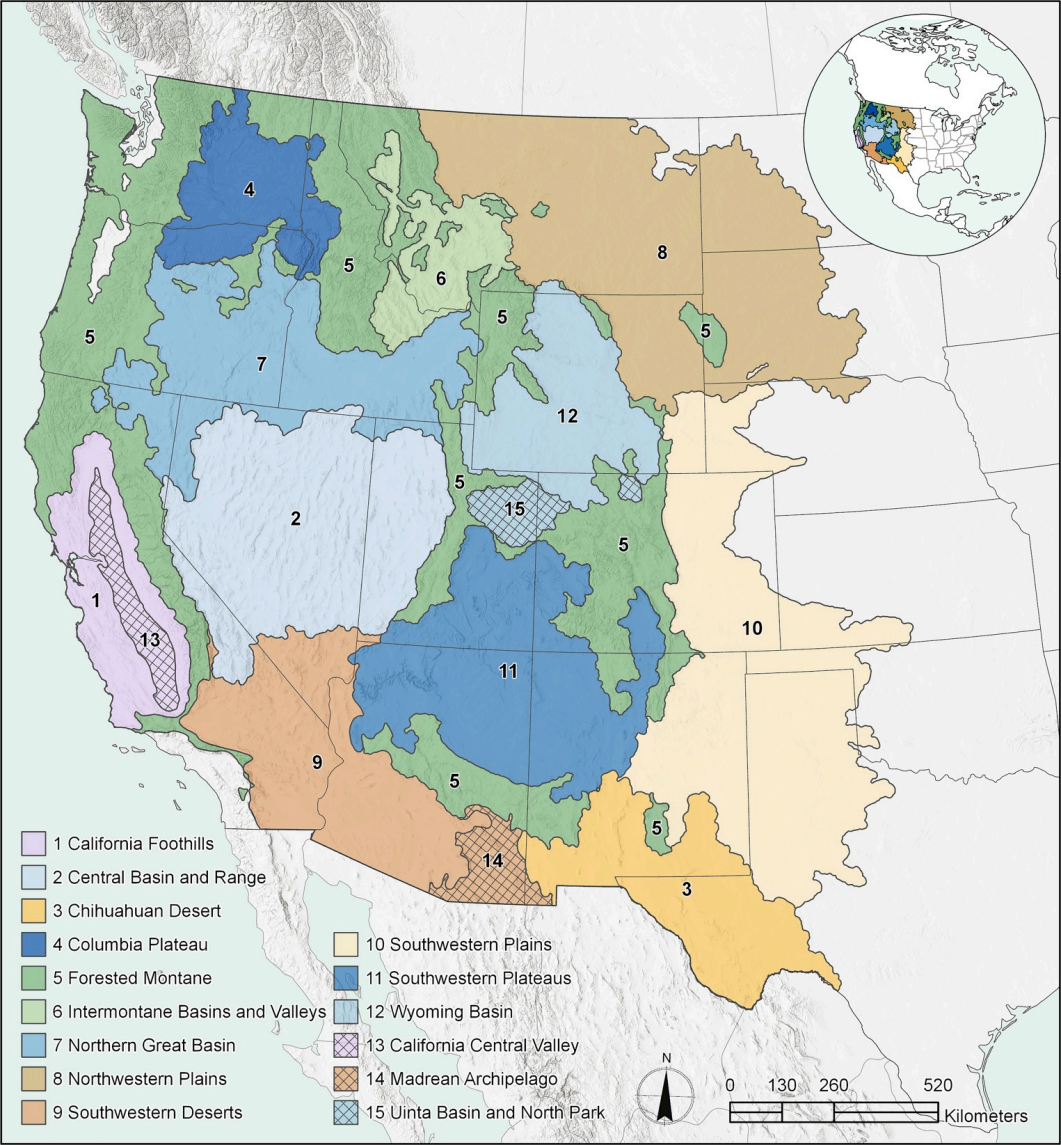
Geographic extent of study area with boundaries of modeling regions (1–12) shown in colored fill and projection regions (13–15) with crosshatch.

Our study area corresponds to the extent of the golden eagle’s breeding range within the conterminous U.S., roughly west of the 100^th^ meridian (Fig 1). We initiated data collection based on the breeding range provided by Kochert et al. [12], and subsequently modified the eastern boundary based on the distribution of nest records and associated habitat features. The study area consists of multiple distinct ecological regions, ranging from southwestern deserts and sagebrush-dominated interior basins to the Rocky Mountains and Great Plains.

### Regional models: Partitioning the golden eagle’s range

We incorporated regional variation in environmental attributes into our modeling process by partitioning the golden eagle’s range in the western coterminous U.S. into 12 discrete modeling regions (Fig 1) and developing models separately for each region (see [37, 45]). We based our modeling regions on Level III Ecoregions established by the North American Commission for Environmental Cooperation (CEC) [48]. Ecoregions differ broadly in terms of vegetation composition, edaphic conditions, landform, and climate. Given the wide range of habitat types throughout our study area, we expected patterns of golden eagle habitat use to be more similar within than among Ecoregions. For example, the majority of golden eagles in the California Coastal Sage, Chaparral and Oak Woodlands Ecoregion nest in oak woodlands and forage predominantly for California ground squirrels (*Oto*s*permophilus beechyi*) in annual grassland habitats [14]. In contrast, golden eagles nesting in the Central Basin and Range Ecoregion rely on hares and rabbits (Family Leporidae) in sagebrush habitats and typically build nests on cliffs and rocky outcrops [12, 49]. Fitting a single SDM across such heterogeneous regions would likely result in an overly general model that would fail to identify region-specific environmental relations and have poor predictive ability in any given region.

We adjusted CEC Ecoregion boundaries, and in some cases combined Ecoregions or portions of Ecoregions, to improve the alignment of modeling region boundaries with habitat gradients (e.g., sagebrush cover) important to golden eagles and their prey. We also masked the modeling regions to exclude areas with features that were not suitable golden eagle habitat (e.g., large bodies of water and playas). Overall we developed and evaluated models within 12 modeling regions (Table 1; Fig 1).

**Table 1:** Characteristics of sample size of golden eagle nest sites and modeling regions used for developing golden eagle nesting area models in the western U.S. Initial number of nests is the full sample of nests we had available to us and that passed our quality control process. Number of thinned nest sites is the sample of nest sites used for model development, after we thinned the initial sample to reduce the chance of pseudoreplication. Modeling region size is total size of modeling region, whereas modeling area size is the union of 20-km radius circles around the thinned nest sites. Modeling area percentage of modeling region is the result of dividing the modeling area by modeling region sizes (x 100). Number of sub-regions is the number of discrete sub-regions within each modeling region.

Modeling Region	Initial number of nests	Number of thinned nests	Modeling region size (km^2^)	Modeling area size (km^2^)	Modeling area percentage of modeling region	Number of sub-regions
California Foothills	2,535	259	100,465	69,383	69	5
Central Basin and Range	7,018	902	321,183	250,469	78	7
Chihuahuan Desert	1,012	118	179,684	51,769	29	3
Columbia Plateau	2,057	279	118,750	63,852	54	6
Forested Montane	3,658	646	711,384	247,495	35	6
Intermontane Basins and Valleys	482	219	87,288	60,516	69	2
Northern Great Basin	4,470	1,050	286,350	213,143	74	6
Northwestern Plains	5,150	977	474,172	274,186	58	8
Southwestern Deserts	1,151	288	251,394	119,902	48	7
Southwestern Plains	471	273	491,295	127,572	26	7
Southwestern Plateaus	1,562	604	306,872	183,734	60	5
Wyoming Basin	3,552	946	154,282	146,310	95	4

### Compiling nest data, sample selection process

We acquired data describing golden eagle nest locations by contacting groups and individuals that collected and maintained such data, including state, federal, tribal, and non-governmental organizations. We solicited all available nest records with locational accuracy <120 m. Because terminology used to describe nest status in the data was inconsistent, we reclassified nest status as “In-Use,” (direct observations of behavior indicative of a nest containing eggs), “Occupied” (documented presence of an adult pair or sign of recent nest repair or use, “Unoccupied” (adult eagles not observed), or “Unknown” [50, 51]. Only In-Use or Occupied nest sites were used in modeling.

A “presence” datum in our dataset was an individual golden eagle nest site supporting an “occupied” or “in-use” nest. However, the initial dataset included many spatially and temporally replicated nest locations due to data redundancies and as a consequence of golden eagle pairs often maintaining multiple nests within their territory, with only one being used in a given year [15]. To address redundancies, we used a hierarchical selection algorithm [45] to thin spatially clustered nest locations into one nest site based on: 1) the most recent observation of a nest with the highest level of nest status (e.g., In-Use > Occupied); and 2) imposing a 3-km threshold (thinning) distance between nests based on typical nearest-neighbor distances and core-use area size [12, 52]. Restricting nest sites included in our data set to those >3-km apart reduced over-representation of intensively surveyed areas where golden eagle territories were very closely spaced (e.g., Central California Coast Ranges; [29] and the Snake River canyon in southwestern Idaho; [53]).

Early in the process of compiling nest location data, it became apparent that large portions of some regions had few or no survey records. To address these apparent data gaps, we worked directly with research institutions and state agencies to conduct targeted surveys for golden eagle nest sites in portions of Texas, Oklahoma, Kansas, Colorado, New Mexico, Utah, Arizona, Montana and Idaho, resulting in 314 additional nest sites.

### Conceptual model: Expert elicitation and literature review

The behavior and general ecology of golden eagles are well known, but most studies of breeding habitat selection have been short-term and conducted in relatively small study areas. As a result, our understanding of the environmental features associated with nest site selection at local *and* landscape scales, and the geographic variation in nesting habitats, is incomplete. To derive an initial set of candidate variables, we reviewed the published literature on the breeding ecology of golden eagles, including habitat use patterns, and solicited expert input from biologists familiar with golden eagle nesting behavior within each modeling region. Given our goal of fitting models comparable across regions, we restricted candidate variables to those available throughout all modeling regions. One limitation of this approach, for example, was that data on the distribution and abundance of important prey of golden eagles were omitted because they were not available for all regions. Some variables were proxies for unmeasured factors believed to more directly influence golden eagle behavior. The final set of variables was a compromise between factors hypothesized to directly or indirectly influence nest site selection with data availability across all regions (see S1 Table for source data for variable estimates).

### Modeling procedure

Each modeling region was analyzed independently. Within each region, we compiled a list of candidate variables known or hypothesized to be related to golden eagle nest site selection. Since we were uncertain about the spatial scale(s) at which variables most influenced site selection, we calculated the focal means and standard deviation of each variable at six spatial scales (circular neighborhoods with radii of 120 m, 0.5, 1.0, 2.0, 3.2, and 6.4 km). This range of scales was chosen to represent variation in environmental features hypothesized to be relevant to golden eagle nest site selection. For example, at finer scales we could estimate local topographic relief associated with nest substrates on cliffs, whereas at coarser scales we could estimate land cover, terrain features and climate variation at scales likely meaningful to nesting golden eagles through foraging habitats and prey resources.

#### Variable identification; variable reduction

Variables estimated for inclusion in our models included environmental attributes such as topographic indices and landform, land cover, climate indices, wind/uplift, vegetation productivity indices, and anthropogenic features (e.g., agricultural and developed areas) hypothesized to affect nest site selection across ecoregions. To span the possible domain of environmental factors likely to affect golden eagle nest site selection, candidate variables were initially classified into discrete categories based on what aspect of the environment they measured (climate indices, land cover, topographic indices, topographic landforms, vegetation indices, wind and uplift indices, development). We followed a two-step process to identify key variables and to reduce the number of redundant variables for each region based on: 1) estimates of their degree of difference between background and nest locations, and 2) measures of multicollinearity. At step 1, candidate variables at all spatial scales were compared by computing the ratio of the mean nest site value to the mean random site value (1,000 random-site locations). For a given variable, we retained the scale with the largest ratio that had <20% of the locations with non-zero values. At step 2, we computed variance inflation factors (VIF), within variable categories, for the subset of “best”-scale variables from step one. We removed variables with VIF ≥4. Within any variable category, the final set of covariates available for selection in the MaxEnt model fitting process generally had pairwise correlations ρ such that -0.5 ≤ ρ ≤ 0.5.

#### Creating the MaxEnt relative nest site density model

All MaxEnt models were fitted using 100,000 background locations, and by evaluating linear, hinge, and quadratic functional relationships for all covariates (referred to as features in MaxEnt). Many surveys for golden eagle nest sites were not based on a probabilistic sampling design. As a result, many nest site locations arose from opportunistic detections and there were extensive areas within each region without knownnest site locations. To address this source of sampling bias, we restricted the selection of background locations to a polygon defined by the union of all 20-km radius circles centered on the thinned presence locations (hereafter referred to as the *modeled area*, which is distinct from, and smaller than, the *modeling region*; see S1 Fig). Circle size was based on the assumption that nesting golden eagles could easily access an area within a 20-km radius area centered on the nest site. We believe that restricting the selection of background locations in this way partially addressed potential sampling biases in the nest location data (see discussion in [54]).

For each region, the initial fitted model included the complete subset of scale-dependent covariates remaining after the variable reduction process. After evaluating this model, all covariates with percent contributions to the model <1.0 were removed, resulting in a *reduced model*. The percent contribution of each covariate was measured in MaxEnt by the increase in gain of the model by modifying the coefficient for that covariate [35]. This process of reducing model complexity resulted in models that performed nearly identical to the initial models but with many fewer covariates.

#### Optimizing the regularization multiplier

The default MaxEnt algorithm includes a regularization penalty parameter to reduce model over-fitting by forcing some covariate coefficients to be zero. The regularization multiplier is user-defined with a default value of 1.0. Lower values of the regularization multiplier predispose areas with high RND to have covariate values very similar to presence locations, whereas higher values produce a more general model [55, 56]. Given the potential influence of this multiplier on covariate selection, we evaluated eight different regularization multiplier values (0.1, 0.5, 0.75, 1, 2, 3, 4, and 5) and optimized each reduced model’s regularization multiplier based on cross-validation results. We conducted cross-validation, whereby each of 10 times we randomly withheld 25% of the nest sites (with replacement) and trained the model with the remaining 75% of nest sites. In all cases, a random sample of 100,000 background locations was used. We then evaluated the distribution of the 25% withheld nest sites predicted to occur among 10 equal-sized RND bins (e.g., 0.0–0.1, 0.1–0.2, 0.2–0.3 …, 0.9–1.0) based on the model fitted to the (75%) training data (see below). We estimated the mean squared prediction error (MSE) between the predicted number of test nest sites occurring within each of the 10 bins relative to the observed number of test nest sites occurring within each bin. Predicted number of nest sites within each bin was calculated as:

$$N_{p(i)}=N_{t}*p_{A(i)}*{AAF}_{i}$$ (1)

Where:

*N*_*p*(*i*)_ = Number of predicted nest sites in bin i

*N*_*t*_ = total number of nest sites in the test sample,

*p*_*A*(*i*)_ = proportion of the area in bin i (see below), and

*AAF*_*i*_ = area-adjusted frequency (AAF) for bin i (see below, following Boyce et al. [42]).

*AAF*_*i*_ was computed with the training data using the following steps:

1. Compute the size (area) of the modeled area (*A*),
2. Partition relative habitat suitability scores (from MaxEnt output) from each cell into K, equal-interval bins.
3. Compute the area (*a*_*i*_) within each bin i = 1, 2, 3, …, K.
4. Count the number of nest sites (*n*_*i*_) within each bin i.
5. Calculate AAF_i_ as:

$${AAF}_{i}=\frac{p_{N(i)}}{p_{A(i)}}$$ (2)

Where:

*n*_*i*_ = number of nests in bin *i*,*i* = 1,2,…,*K*

*a*_*i*_ = area within bin *i*

*N* = total number of nests = $\sum_{i}n_{i}$

*A* = total area of study region = $\sum_{i}a_{i}$

*p*_*A*(*i*)_ = proportion of area in bin *i* = $\frac{a_{i}}{A}$

*p*_*N*(*i*)_ = proportion of nests in bin *i* = $\frac{n_{i}}{N}$

Assuming nest sites to be randomly distributed across the modeled area—the null hypothesis of no relationship with measured environmental covariates—the predicted number of nest sites within each bin is proportional to the modeled area within a given RND bin (i.e., AAF = 1). Area-adjusted frequencies (AAF) were initially proposed by Boyce et al. [42] as a method of evaluating resource selection functions. Evaluating the rank correlation of bin rank and AAF rank (referred to as the Boyce Index by Hirzel et al. [57]) provides an assessment of the extent to which an estimated model diverges from expectations under a null hypothesis of a random distribution of breeding locations [42].

For each regularization multiplier value, cross-validation resulted in 100 predicted and 100 observed values (10 replicates by 10 bins), and the MSE was calculated as the average squared difference between *N*_*p(i)*_ and *n*_*i*_. We used the regularization multiplier value with the smallest MSE to fit our final models.

Assuming a sample of N nest sites within a given region, the fitted model predicts how those N nest sites are distributed among 10 RND bins. The difference between observed and predicted frequencies across bins is a measure of model fit. Note that in contrast to the more commonly reported MaxEnt output metrics (raw or logistic estimates of relative habitat suitability), our estimates are interpreted as relative density. In addition, comparing AAF values among bins can be used to estimate the extent to which nest density differs among bins.

#### Model evaluation with independent data

For nine regions (all except the Chihuahuan Desert, Intermontane Basins and Valleys, and Southwestern Plateau regions), additional nest location data became available to us after final models had been fit. This provided an opportunity for model evaluation based on independent data not used in model fitting. For those modeling regions with independent data, we evaluated the final model’s ability to accurately predict the distribution of test nest sites among RND bins (using Eq 1) after thinning the new nest locations so that they were ≥ 3 km from each other and from training locations. The predicted number of nest sites in each bin requires an estimate of the proportion of area within each of the 10 RND bins. Therefore, we centered 20-km radius circles on each of the thinned independent nest sites and estimated the proportion of area (union of circles) in each RND bin. We then compared the number of nest sites predicted to be in each RND bin to the actual number occurring in each bin.

#### Geographic evaluation

To evaluate geographic variation in model performance, we subdivided each region into two to eight sub-regions (Figs in S2 Fig) based on U.S. Forest Service Ecological Sections [58]. For each sub-region, we applied our final model to the entire modeled area and estimated the number of nest sites predicted to occur within each of 10 RND bins and compared those predictions to the observed number of nest sites within each bin. Estimated number of nest sites within each RND bin within each sub-region was based on an adjustment to Eq (1) as follows:

$$N_{p(i)SR(j)}=N_{t}*p_{A(i)}*p_{SR(j)}*{AAF}_{i}$$ (3)

Where:

*N*_*p*(*i*)*SR*(*j*)_ = Number of predicted nest sites in bin *i* of sub-region *j*,

*p*_*SR*(*j*)_ = proportion of the modeled area in sub-region *j*, *j* = 1, 2, 3,…, K.

The geographic evaluation of model performance within sub-regions provided insights into the spatial variation in model performance within a modeling region. If the model performed poorly in a particular sub-region, it would suggest that it might be reasonable to consider: 1) developing a sub-region specific model; 2) focusing future field survey efforts in that sub-region to increase the sample size of nest sites; or, 3) utilizing more nuanced considerations of risk assessment/conservation opportunities in those areas.

#### Projecting models

Each model was developed and evaluated within the modeling area and subsequently projected to the remaining area of its modeling region (Figs in S1 Fig). We provide descriptive statistics on the proportion of the modeling region that the modeling area represented, and the amount of modeling areas and modeling regions in each of 10 RND bins.

#### Projecting models outside of modeling regions

In three cases we projected models to portions of ecoregions outside of the focal modeling region that had similar environmental conditions (Figs in S1 Fig). This allowed us to project models to nearly all of the western U.S. study area, including areas for which data were lacking. We projected the California Foothills model to the California Central Valley due to the near absence of available training data, and limited extent of potentially suitable habitat in that region. The Madrean Archipelago also had a small sample of nest sites, and was included by projecting the Southwestern Deserts model. Although the Uinta Basin and North Park areas likely had an adequate sample of nest sites to generate a new model, they contained highly similar environmental conditions to those in the adjacent Wyoming Basin region for which we had a model. We therefore projected the Wyoming Basin model to the Uinta Basin and North Park. Performance of the projected models was evaluated by comparing the number of observed nest sites to the number of predicted (using Eq 1) nest sites in each of 10 RND bins, where observed nest sites were independent nest locations thinned by the same criteria as the training data.

## Results

We compiled 160,510 golden eagle nest records and identified 33,118 that met our criteria for discreteness, occupancy status and spatial accuracy. After the thinning process, the presence sample included 6,561 nest sites, ranging from 118–1,050 (($\bar{x}$ = 547) among modeling regions (Table 1).

Among regions we considered 276–578 variables. After selecting the “best” scale for each variable and conducting VIF analyses, the number of covariates evaluated in initial models ranged from 18–34 (Table 2) among regions. Final models included from six—14 covariates (Table 2). The most influential covariate in each region’s final model was related to terrain steepness (mean or variation) at local spatial scales (120 m for 11 regions and 1 km for one region; Table 3, Figs in S3 Fig). Overall, individual topographic indices covariates contributed 37.2–94.2% to fitting each full model (Table 3, Figs in S3 Fig). Additional covariates across modeling region’s models included landcover, climate indices, wind and uplift indices, and developed areas. Although terrain covariates weren’t identical across all modeling regions’ final models, the functional forms of the terrain covariates in each region were generally quite similar. RND increased with increasing steepness, generally in a threshold shape with the positive influence of steepness on RND declining above moderate steepness values. Final models for the California Foothills, Forested Montane, Intermontane Basins and Valleys, and Wyoming Basin included sagebrush or grassland habitat covariates, at 0.5–6.4 km scales, as influential covariates (Table 3, Figs in S3 Fig), and in each case showed a strongly positive relationship (linear or threshold) between RND and amount of, or variation in amount of, grassland or sagebrush habitat. In contrast, for the Northwestern Plains’ final model, SD of cultivated cropland was an influential covariate (Table 3, Figs in S3 Fig), and showed an inverse and linear relationship with RND.

**Table 2:** Number of initial variables considered, number of covariates in the initial model, and number of covariates in each modeling region’s final model.

Modeling Region	Initial number of variables	Number of covariates in initial model	Number of covariates in final model
California Foothills	578	23	10
Central Basin and Range	457	22	10
Chihuahuan Desert	566	18	6
Columbia Plateau	457	25	13
Forested Montane	566	34	11
Intermontane Basins and Valleys	566	28	12
Northern Great Basin	276	21	11
Northwestern Plains	457	20	12
Southwestern Deserts	578	31	8
Southwestern Plains	511	22	10
Southwestern Plateaus	578	33	8
Wyoming Basin	284	24	14

**Table 3:** List of covariates that contributed ≥5% to each modeling region’s final model. Values beneath each modeling region are the covariate’s percentage contribution to fitting each final model.

Covariate category	Covariate description	Mean or Standard Deviation	Scale	Modeling Region											
				CAFO	CBRA	CHDE	COPT	FOMO	IMBV	NGRB	NWPL	SWDE	SWPL	SWPT	WYBA
Climate Indices	Annual moisture index	Mean	0.5 km							7.5					
	Degree days above 5 degrees C	Mean	3.2 km												5.4
Landcover	Proportion alfalfa landcover	SD	3.2 km					6.8							
	Proportion deciduous, evergreen, and mixed forest	Mean	6.4 km	5.9											
	Proportion of cottonwood landcover	Mean	1.0 km								7.2				
	Proportion of cultivated cropland landcover	SD	6.4 km								10.6				
	Proportion of grassland landcover	Mean	3.2 km	27.8											
	Proportion of low and tall sagebrush landcover	Mean	6.4 km												10.1
	Proportion of sparsely vegetated landcover	Mean	6.4 km								15.0				
	Proportion of tall sagebrush landcover	Mean	1.0 km				5.0								
	Proportion of tall sagebrush landcover	Mean	3.2 km					13.8							
	Proportion of tall sagebrush landcover	SD	0.5 km						12.5						
	Proportion pinyon-juniper and juniper woodland	SD	3.2 km					7.1							
	Proportion riparian woodland landcover	SD	6.4 km		6.6										
	Proportion shrub landcover	Mean	3.2 km					5.7							
Topographic Indices	Local Elevational Difference (LED)	SD	120 m	47.2				37.2							
	Topographic wetness index (TWI)	Mean	120 m						10.5						
	Terrain elevation	SD	120 m									66.6			
	Terrain Ruggedness Index (TRI)	SD	120 m			94.2							80.9		
	Terrain slope	Mean	120 m									16.8			
	Terrain slope	SD	120 m		71.4			14.4	37.1		54.2			71.7	
Topographic Landforms	Proportion of flat landforms	SD	1.0 km						9.7						
	Proportion of flat landforms	SD	2.0 km				10.5								
	Proportion of gently sloping landforms	SD	0.5 km	11.6	7.3					18.9					
	Proportion of ridge landforms	Mean	120 m											14.3	
	Proportion of ridge landforms	Mean	2.0 km				7.1								
	Proportion of steeply sloping landforms	Mean	120 m				63.6								
	Proportion of steeply sloping landforms	Mean	1.0 km										11.0		
	Proportion of steeply sloping landforms	SD	120 m												60.4
	Proportion of steeply sloping landforms	SD	1.0 km							51.7					
	Proportion of valley landforms	Mean	1.0 km						8.4						
Wind and Uplift	Wind power class at 50 m	SD	0.5 km									7.7			
Development	Proportion of road landcover	SD	3.2 km						7.1						

CAFO = California Foothills, CBRA = Central Basin and Range, CHDE = Chihuahuan Desert, COPT = Columbia Plateau, FOMO = Forested Montane, IMBV = Intermontane Basins and Valleys, NGRB = Northern Great Basin, NWPL = Northwestern Plains, SWDE = Southwestern Deserts, SWPL = Southwestern Plains, SWPT = Southwestern Plateaus, WYBA = Wyoming Basin.

The optimal regularization multiplier value varied from 0.75–6 across modeling regions (Table 4). Because the lowest MSE for the Southwestern Plains, Southwestern Deserts, and California Foothills was found at the highest regularization value initially assessed (5), we evaluated those regions at multiplier values of 6 and 7. We found that the MSE increased at higher values for the Southwestern Plains and Southwestern Deserts, but decreased for the California Foothills (Table 4). After models were re-estimated using the optimized regularization multiplier, the contribution of a small number of covariates dropped to below 1% but were retained in the models.

**Table 4:** Mean squared prediction error (MSE) among regularization multiplier values for each modeling regions’ final golden eagle nesting area model. The lowest MSE value is italicized and has gray shading. For the Southwestern Plains, Southwestern Deserts, and California Foothills modeling regions, the lowest MSE was found at regularization values of 5 (the largest value we initially evaluated), so we evaluated two larger regularization multiplier values (6 and 7) to determine whether MSE continued to decline with increasing regularization.

Modeling Region	Regularization Value									
	0.1	0.5	0.75	1	2	3	4	5	6	7
California Foothills	10.89	11.03	8.13	7.5	10.36	7.17	9.23	7.88	*6*.*21*	8.31
Central Basin and Range	35.4	30.08	*28*.*94*	29.3	31.59	34.7	36.37	38.8		
Chihuahuan Desert	5.46	4.17	3.12	3.62	3.29	*2*.*82*	3.75	3.33		
Columbia Plateau	14.78	12.6	12.4	10.85	8.66	*8*.*36*	9.97	10.71		
Forested Montane	27.87	22.08	27.34	22.23	26.38	20.87	*14*.*12*	21.3		
Intermontane Basins and Valleys	17.62	8.74	7.51	7.57	*5*.*16*	7.42	7	8.42		
Northern Great Basin	30.02	32.52	32.75	*26*.*25*	36.11	34.56	32.95	31.16		
Northwestern Plains	33.92	31.96	33.41	29.65	41.59	25.7	*25*.*21*	26.86		
Southwestern Deserts	12.35	7.62	9.74	9.66	5.98	8.55	5.95	*5*.*83*	11.3	7.32
Southwestern Plains	12.26	9.95	9.62	9.87	8.38	7.06	6.97	*6*.*71*	8.98	8.85
Southwestern Plateaus	21.5	16.03	*14*.*65*	18.67	19.38	16.92	18.18	14.97		
Wyoming Basin	40.14	34.52	33.3	43.73	34.95	*25*.*59*	28.21	40.07		

### Model performance

#### Predicted versus observed densities: Cross-validation

**Fig 2:**
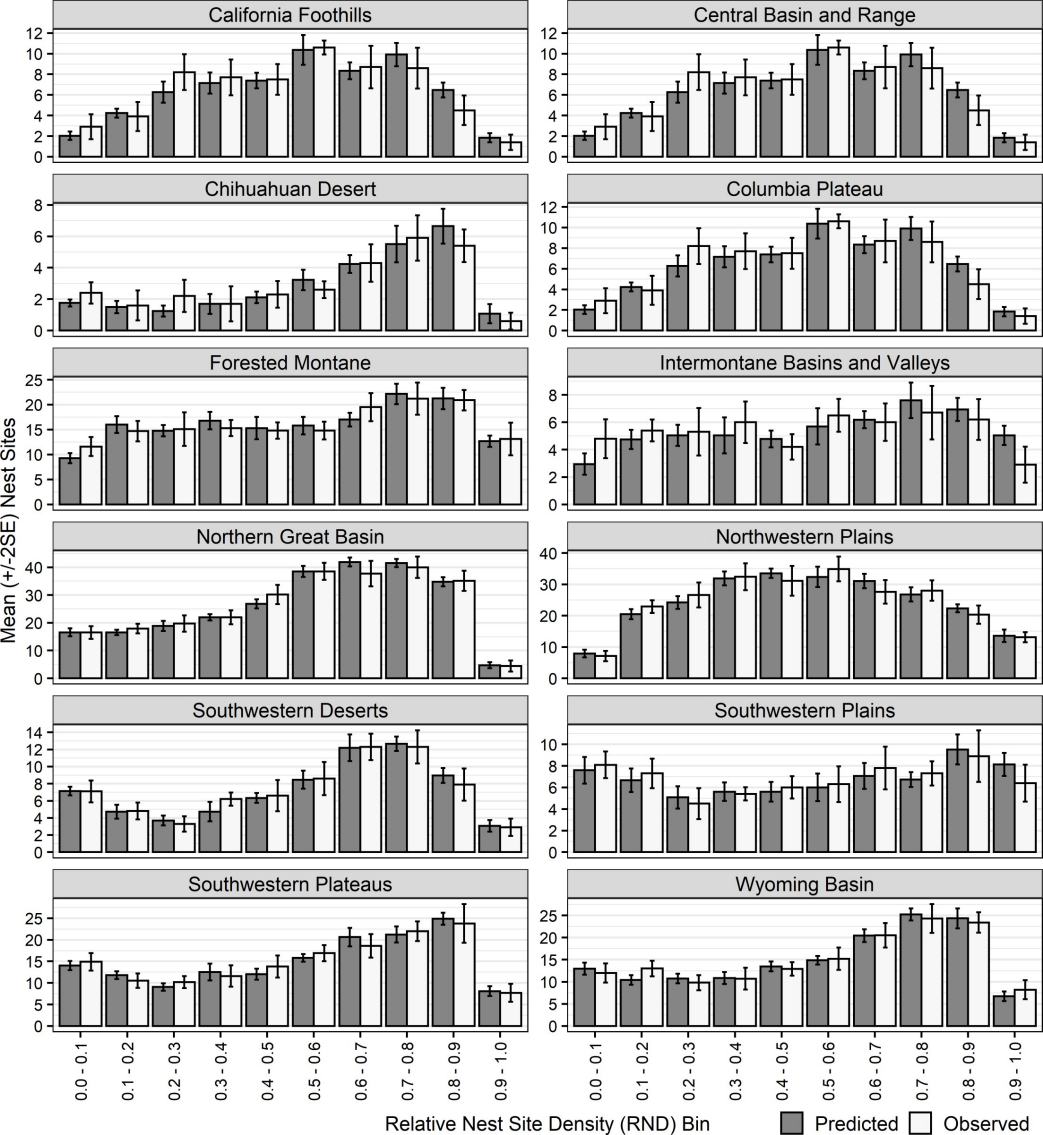
Bar graphs of mean (±2SE) predicted and observed number of golden eagle nest sites in each of 10 relative nest density (RND) bins within each modeling region. Both predicted and observed numbers were from the 25% withheld data from 10 cross-validations of each region’s model.

Cross-validation revealed that for each of the 12 modeling regions, model predictions were consistent with the distribution of presence data in the evaluation data set (Fig 2). Deviations between predictions and withheld data were greatest for the Columbia Plateau, Southwestern Plains, Intermontane Basins and Valleys, California Foothills, and Chihuahuan Desert modeling regions, which had some of the smallest sample sizes of nest sites coupled with modeling areas being a smaller percentage of their respective regions (Table 1).

#### Predicted versus observed densities in sub-regions

**Fig 3:**
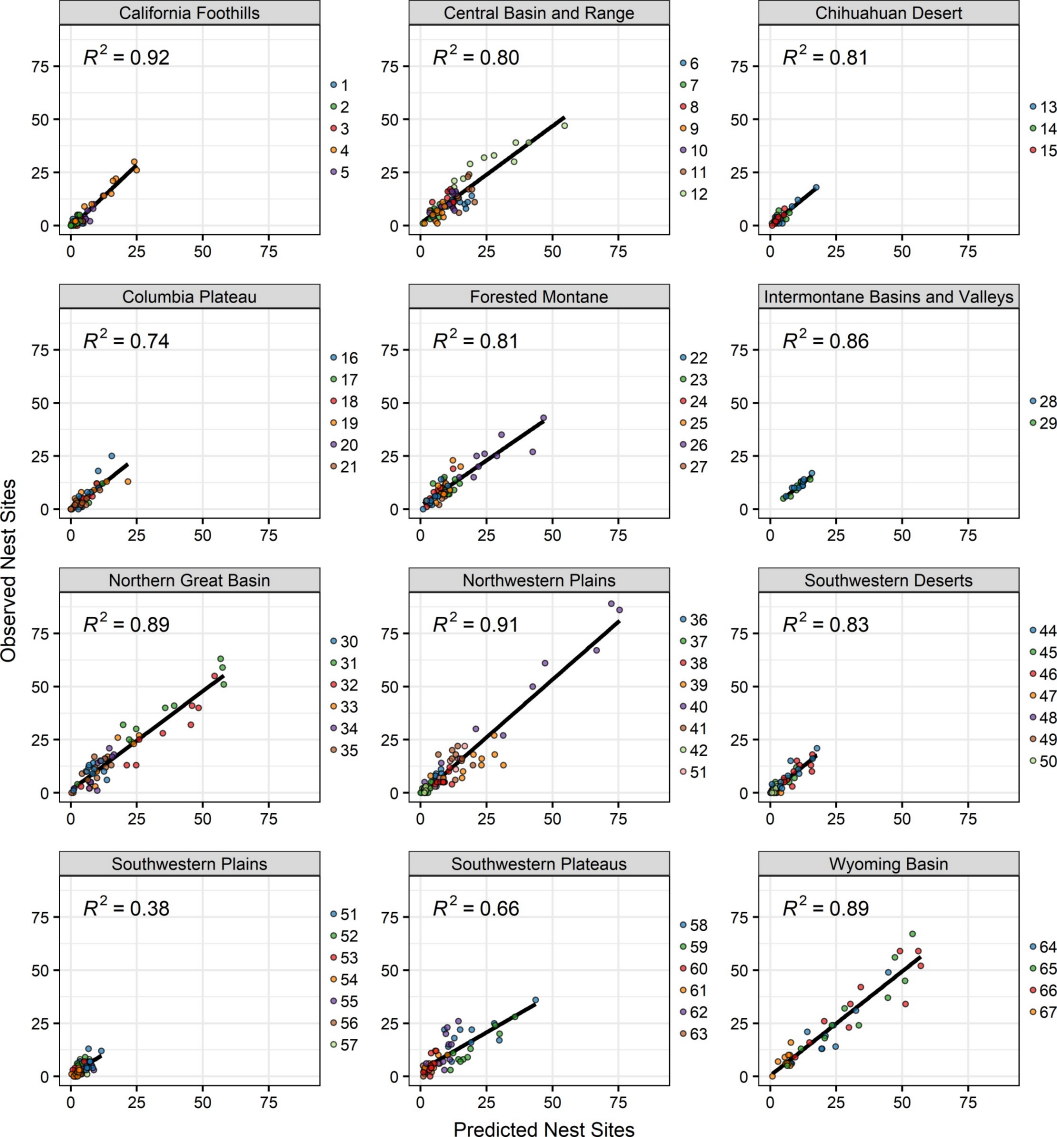
Scatterplots of predicted versus observed number of golden eagle nest sites among 10 RND bins within each modeling region’s sub-regions. Numbers in each region’s plot refer to sub-regions. Sub-region names can be found in S2 Fig.

Overall, regional models predicted nest site densities of golden eagles within sub-regions very well (Fig 3). Coefficients of determination between estimated number of nest sites within RND bins within sub-regions and observed number of golden eagle nest sites within RND bins ranged from 0.36 (Southwestern Plains) to 0.92 (Northwestern Plains; and nine of 12 r^2^ values were >0.75; Fig 3). In no cases did models predict extremely small or large numbers of nest sites when the opposite reality existed.

#### Independent test data

**Fig 4:**
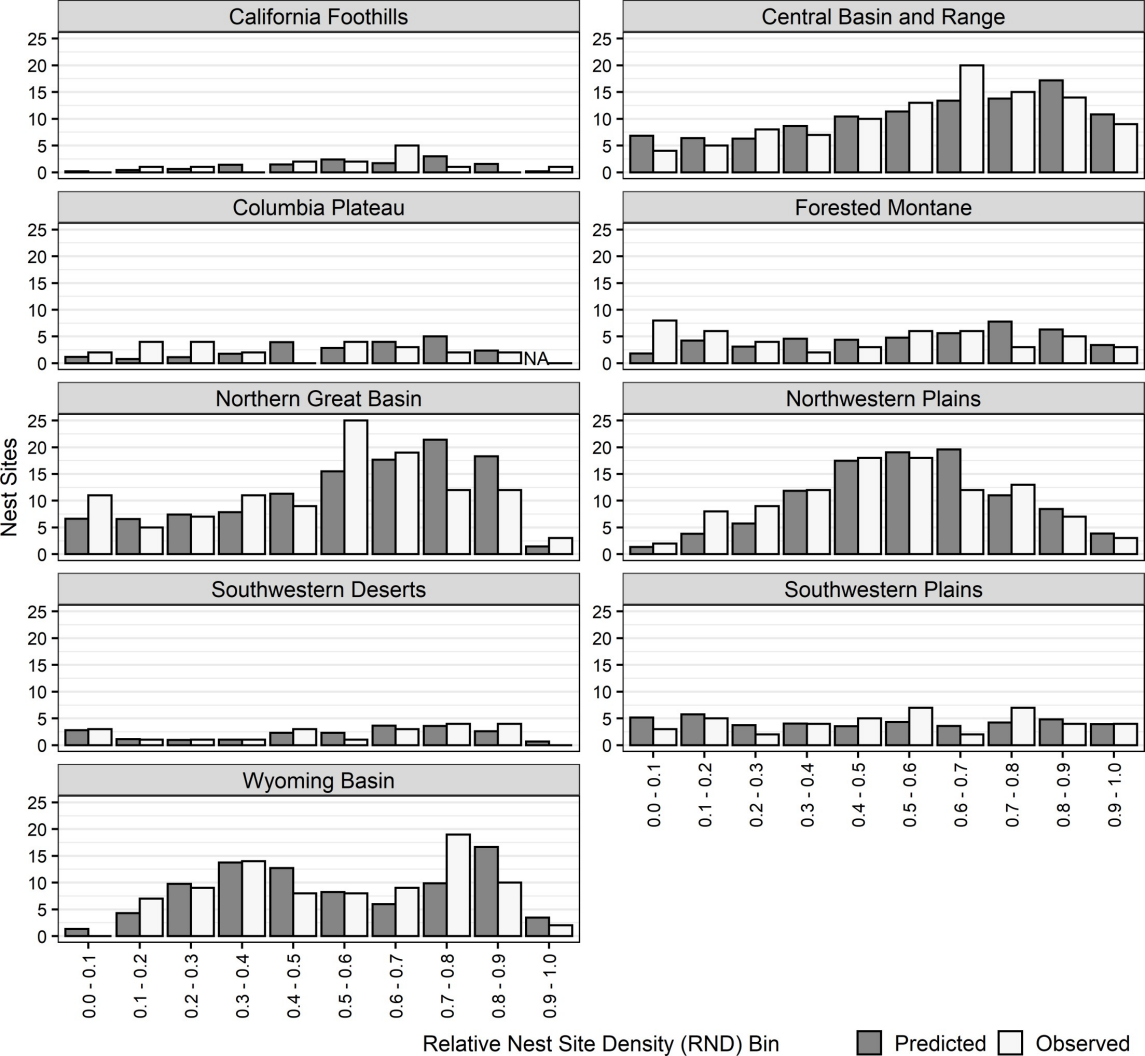
Bar chart of distribution of observed and predicted nests sites (observed–predicted) within RND bins for 553 independent nest sites within nine modeling regions.

We obtained 553 independent nest site locations within nine modeling regions. The number of independent nest sites by region ranged from 13 (California Foothills) to 114 (Northern Great Basin). We compared the number of predicted to number of observed nest sites occurring within each RND bin within each region, and generally found only minor differences between predicted and observed nest sites within RND bins. Among all independent nest sites, 40% of the differences, between predicted and observed, were less than 1, 71% were less than 2.5, and 91% were less than 5; the largest difference was 9.5 (Fig 4).

#### Projecting models outside of modeling regions

**Fig 5:**
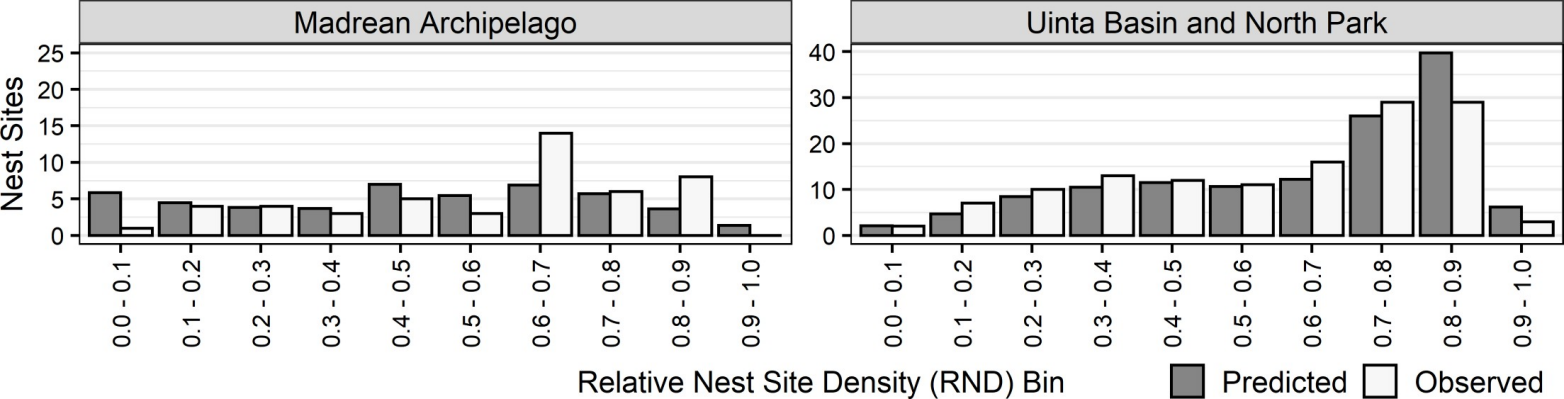
Bar plots of the distribution of predicted and observed nest sites among RND bins in the Madrean Archipelago, and Uinta Basin and North Park.

Three regional models were projected to large areas with similar conditions. We had independent nest site data for the Madrean Archipelago (n = 48 nest sites) and the Uinta Basin and North Park (n = 132 nest sites), and used those to evaluate how well the projected models predicted the distribution of independent nest sites among RND bins. The Southwestern Deserts Model was projected to the Madrean Archipelago, and in general the model under-predicted the distribution of nest sites within higher RND (>0.5) bins (Fig 5) and over-predicted the distribution of nest sites with lower RND (<0.5) values. The Wyoming Basin model was projected to the Uinta Basin and North Park, and it quite accurately predicted the distribution of nest sites among RND bins (Fig 5). Due to the small number of nest sites in the California Central Valley, we were unable to meaningfully evaluate the projection of the California Foothills model.

### Area-adjusted frequencies

The rank correlation between bin and AAF ranks was > 0.96 for all regions (it was 1.0 for 10 regions) indicating a highly non-random distribution of nest sites among RND bins (Table 5). In low RND bins the magnitude of difference from random expectation was not as pronounced as it was for high RND bins (Table 5). For example, among all modeling regions, the highest RND bins (0.9–1.0) had between 21.0–166.2 times more nest sites than expected based on area within that bin, whereas the lowest RND bin (0–0.1) had from 4.2–16 times fewer nest sites than expected (Table 5). Within modeling regions, variation in AAF values among bins revealed extremely large differences in relative densities of nest sites. For example, the factor by which the highest bin’s relative nest site density exceeded the lowest bin’s ranged from 132–2,660 among regions (Table 6).

**Table 5:** Area Adjusted Frequencies of each modeling regions’ final model among RND bins. The Columbia Plateau modeling region had too few observations in bin 0.9–1 to be meaningfully estimated.

RND Bin	Modeling Region											
	CAFO	CBRA	CHDE	COPT	FOMO	IMBV	NGRB	NWPL	SWDE	SWPL	SWPT	WYBA
0.0–0.1	0.160	0.143	0.062	0.091	0.113	0.116	0.101	0.104	0.146	0.171	0.118	0.241
0.1–0.2	0.300	0.441	2.724	0.409	0.529	0.424	0.485	0.323	0.574	0.529	0.934	0.290
0.2–0.3	0.403	0.859	5.050	0.976	0.705	0.824	0.952	0.572	0.840	1.369	1.375	0.517
0.3–0.4	0.919	1.897	11.307	1.947	1.667	0.979	1.397	1.126	1.254	2.721	2.750	0.888
0.4–0.5	1.197	3.278	18.813	5.194	2.374	2.501	2.538	1.970	3.605	3.534	4.858	1.545
0.5–0.6	2.798	4.912	18.197	4.723	3.805	4.149	4.618	3.205	4.585	6.153	7.395	2.287
0.6–0.7	2.873	7.965	68.391	9.308	6.688	5.952	7.323	6.020	10.201	7.394	15.187	2.797
0.7–0.8	8.554	12.000	68.006	17.691	14.781	14.719	13.872	7.495	16.626	13.268	18.006	5.785
0.8–0.9	11.942	25.908	212.490	26.360	23.203	22.798	26.150	15.039	27.620	25.829	44.545	14.853
0.9–1.0	21.007	66.911	166.180		56.634	52.560	24.460	31.820	52.345	68.430	113.590	32.946

CAFO = California Foothills, CBRA = Central Basin and Range, CHDE = Chihuahuan Desert, COPT = Columbia Plateau, FOMO = Forested Montane, IMBV = Intermontane Basins and Valleys, NGRB = Northern Great Basin, NWPL = Northwestern Plains, SWDE = Southwestern Deserts, SWPL = Southwestern Plains, SWPT = Southwestern Plateaus, WYBA = Wyoming Basin.

**Table 6:** Factor by which golden eagle nest density varies from the lowest bin for each modeling regions’ final model.

RND Bin	Modeling Region											
	CAFO	CBRA	CHDE	COPT	FOMO	IMBV	NGRB	NWPL	SWDE	SWPL	SWPT	WYBA
0.0–0.1	1.0	1.0	1.0	1.0	1.0	1.0	1.0	1.0	1.0	1.0	1.0	1.0
0.1–0.2	1.9	3.1	43.6	4.5	4.7	3.6	4.8	3.1	3.9	3.1	7.9	1.2
0.2–0.3	2.5	6.0	80.8	10.7	6.2	7.1	9.4	5.5	5.8	8.0	11.6	2.1
0.3–0.4	5.8	13.2	181.0	21.4	14.7	8.4	13.8	10.8	8.6	15.9	23.2	3.7
0.4–0.5	7.5	22.9	301.2	57.1	21.0	21.5	25.0	18.9	24.7	20.6	41.1	6.4
0.5–0.6	17.5	34.3	291.3	51.9	33.6	35.6	45.5	30.8	31.5	35.9	62.5	9.5
0.6–0.7	18.0	55.6	1094.9	102.3	59.1	51.1	72.2	57.8	70.0	43.1	128.4	11.6
0.7–0.8	53.6	83.7	1088.7	194.4	130.7	126.4	136.8	72.0	114.1	77.4	152.2	24.0
0.8–0.9	74.8	180.8	3401.7	289.6	205.1	195.8	257.9	144.4	189.5	150.6	376.6	61.7
0.9–1.0	131.6	466.8	2660.4		500.6	451.3	241.3	305.6	359.2	399.1	960.2	136.9

CAFO = California Foothills, CBRA = Central Basin and Range, CHDE = Chihuahuan Desert, COPT = Columbia Plateau, FOMO = Forested Montane, IMBV = Intermontane Basins and Valleys, NGRB = Northern Great Basin, NWPL = Northwestern Plains, SWDE = Southwestern Deserts, SWPL = Southwestern Plains, SWPT = Southwestern Plateaus, WYBA = Wyoming Basin.

### Nest site distribution among RND bins in modeling areas

**Fig 6:**
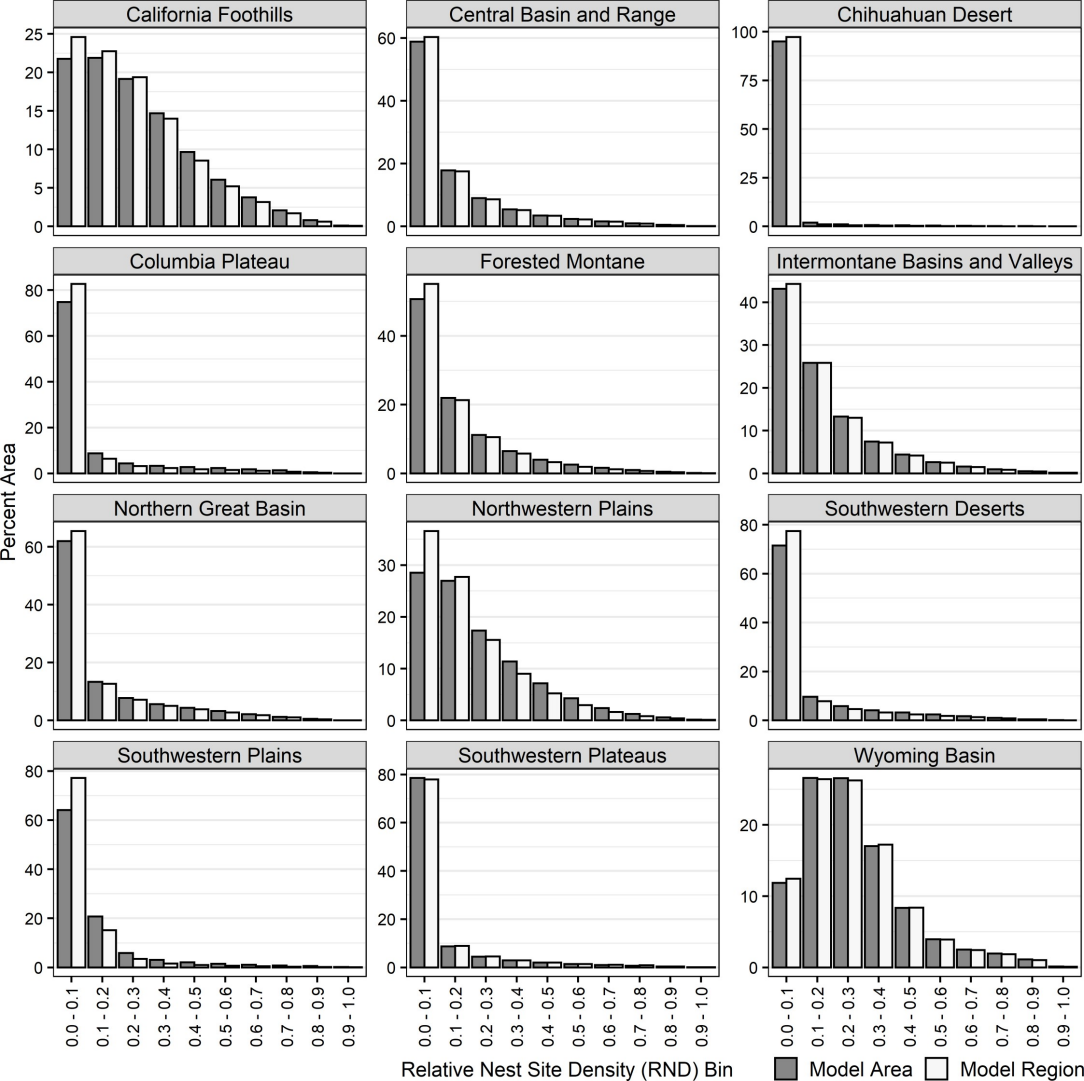
Percent area within ten equal-sized relative nest site density (RND) bins in modeling areas (gray bars) and modeling regions (white bars).

**Fig 7:**
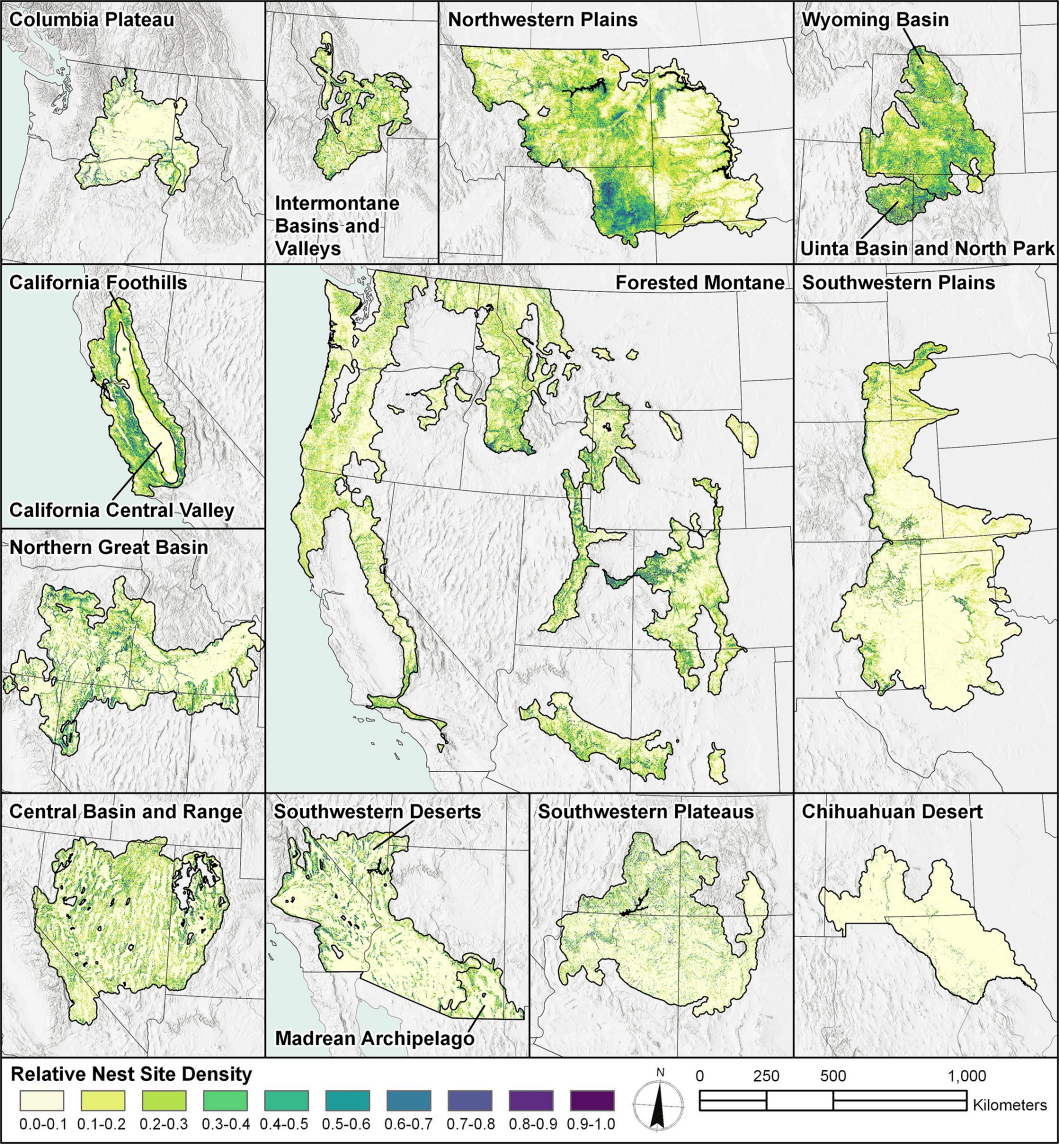
Maps of the distribution of RND in all regions.

All modeling areas and regions were primarily composed of low RND areas, while high RND areas had limited spatial extent (Figs 6 and 7, S4 Fig, and S2 Table). Hence, a disproportionately large number of nest sites were predicted to occur on a relatively small percentage of the landscape. For example, we estimated that areas with RND values <0.3 represented from 62.8–97.8% ($\bar{x}$ = 82.5%) of each modeling area, and those areas contained from 14.7–30.0% ($\bar{x}$ = 22.1%) of the nest sites. In contrast, areas with RND values >0.5 represented from 1.0–12.8% ($\bar{x}$= 6.3%) of modeling areas, and those areas contained from 47.7–66.9% ($\bar{x}$ = 57.3%) of the nest sites.

### Distribution of RND within modeling areas and modeling regions

Modeling areas represented from 26% - 95% ($\bar{x}$ = 58%) of the extent of their respective modeling regions. To evaluate if modeling areas were representative of their regions, we compared the percentage of each modeling area and its respective region represented within each RND bin (Fig 6). In all regions, the largest differences occurred for the lowest valued RND bin; in 11 of 12 cases, modeling areas had less of the lowest RND bin than the modeling regions, with the largest difference being 13 percentage points (SWPL; mean difference of absolute values was 4.3 percentage points). For higher valued RND bins (RND values >0.5), 59 of 60 differences were <1 percentage point, with the largest difference being 1.3 percentage points.

In general, the majority area in all regions had low RND values. For example, the mean percent of regions with RND values <0.3 was 85.1 (range = 65.1–98.9), and seven of the 12 modeling regions had >60% of their area with RND values ≤ 0.1 (Fig 6, Table B in S2 Table). In contrast, high-valued RND areas were a very small percentage of each region (Fig 6, Table B in S2 Table), with RND bin 0.9–1.0 being <0.14% of each region’s area. RND values >0.5 occurred in 0.51% - 10.7% of the 12 modeling regions.

## Discussion

We developed relative nest site density models for golden eagles for use in large-scale conservation prioritization efforts. Within all modeling regions only a small proportion of each region’s area was predicted to have high RND values, and large areas had relatively low RND values. The highest RND bin intervals were estimated to have 132–2,660 times greater nesting densities than the lowest bin interval (Table 6). Thus, prioritization of conservation actions on a small portion of each region could have a disproportionately large benefit to breeding golden eagles.

**Fig 8:**
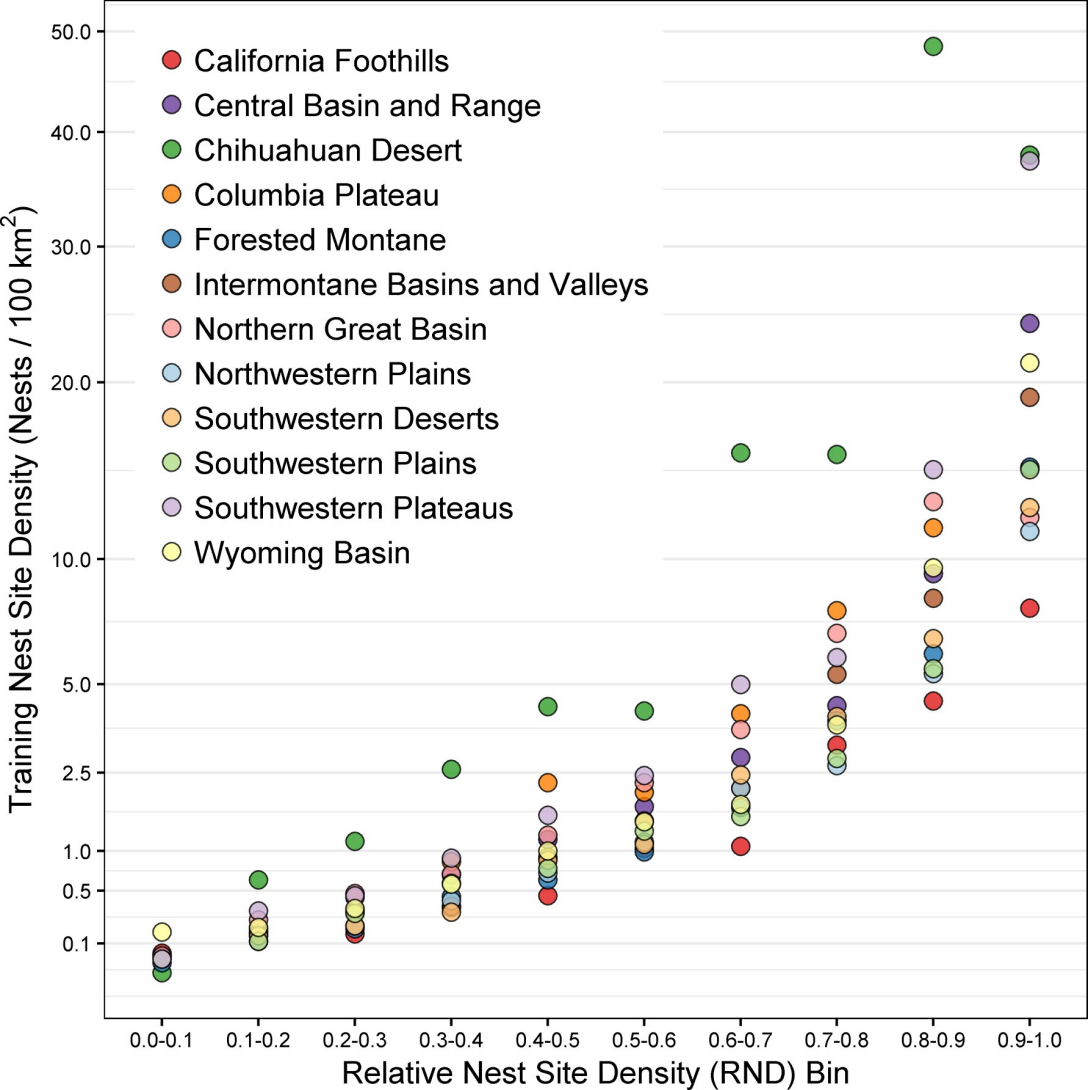
Observed densities of golden eagle nest sites used in model training within ten equal-sized relative nest site density (RND) bins, for each of twelve modeling regions.

Given the large geographic extent of the golden eagle’s range in the western U.S., our subdivision of this area into discrete modeling regions was warranted, and resulted in good to excellent models. Explicit consideration of unique local and regional scale habitat associations, population characteristics and constraints is essential for effective conservation planning for broadly distributed species such as greater sage-grouse (*Centrocercus urophasianus*)[59], northern spotted owls (*Strix occidentalis caurina*)[3], and golden eagles [60, 61]. A consequence of developing multiple regional models, however, is that relative nest density values (RND and AAF) from individual regions are not directly comparable among regions. For example, an RND value of 0.8 in one region may correspond to a different observed density than RND of 0.8 in another region (Fig 8).

We sought models that would provide reliable predictions of relative density of golden eagle nest sites, as opposed to describing and quantifying the species’ niche. Nonetheless, it is important that model predictions are interpretable if they are to be incorporated into management and conservation planning. Our models reflected well-known patterns of habitat selection exhibited by golden eagles across the western U.S. and corresponded well to previously published descriptions and models of breeding habitat [62, 45]. Topographic features corresponding to nest substrates on cliffs and steep terrain at finer scales (120 m– 1.0 km) were important predictors in all model regions (Table 3). This finding is consistent with empirical studies and regional models of breeding habitat selection in areas where golden eagles nest predominantly on cliffs and rock outcrops [62, 45]. Field studies have consistently identified variable terrain and gentle slopes, along with wind and orographic uplift variables, as important predictors of golden eagle space-use within home ranges [52, 62–64]; in our study these variables were moderately important covariates in 8 of 12 modeling regions (Figs in S3 Fig). Our models also incorporated land cover variables thought to influence prey availability positively, such as sagebrush and grassland cover, and riparian habitat; or negatively, including barren areas, cropland, and introduced annual grasses (e.g. cheatgrass, *Bromus tectorum*) [52, 64, 65]. At coarser scales (1.0–6.4 km) surrounding nest sites, model predictions were influenced by regionally specific suites of covariates related to foraging and prey habitats. In the California Foothills modeling region, where most golden eagles nest in woodlands, our model predictions were strongly influenced by the combination of rugged terrain, grass cover and woodland cover covariates (Figs in S3 Fig). These variables were found to be correlated with spatial variation in occupancy and reproduction of golden eagle territories studied by Wiens et al. [2] in the Diablo Range portion of our California Foothills modeling region.

Cross-validation showed that all models accurately predicted numbers of golden eagle nest sites within RND bins, and geographic evaluation showed consistently good predictive success among sub-regions for nine regions. We believe model results for the Central Basin and Range, Northwestern Plains, Northern Great Basin, Wyoming Basin, Southwestern Plateaus, Southwestern Deserts, Forested Montane, and Chihuahuan Desert were strongly validated. Models for the Columbia Plateau, Southwestern Plains, Intermontane Basins and Valleys, and California Foothills were less robust, but still performed quite well. For these four regions, models could likely be improved by increasing samples of nest sites from larger portions of each region. Variation in model quality among regions was related to variation in sample size and dispersion patterns of thinned nest sites, as well as the degree of contrast between high and low RND in regions. In general, as sample size increased and as modeling areas represented larger portions of modeling regions, model predictive power increased, and models were more robust. The Chihuahuan Desert, for example, had by far the largest percentage (95%) of its area in the lowest RND bin. Although we had a small sample of thinned nest sites in the Chihuahuan Desert, the model performed well, we suspect owing to so much of the region being of poor nesting suitability (i.e., high contrast).

When projecting the Southwestern Deserts, and Wyoming Basin models to the Madrean Archipelago, and Uinta Basin and North Park, respectively, we found that only the model projected to the Uinta Basin and North Park resulted in accurate predictions of the distribution of independent nest sites (Fig 5). Although the projected models may be the best current estimates of the distribution of RND in the projected to areas, we would recommend development of RND models specifically within the Madrean Archipelago.

Converting SDM continuous outputs into binary classifications results in the loss of information [66], especially considering that we can estimate the magnitude of differences in nest-site density among RND bins within a modeling region by calculating AAF values. A binary classification of our RND output within each region would have combined areas with large differences in densities of golden eagle nest sites in both the “suitable” and “unsuitable” or “present” and “absent” categories. For example, for all regions, the AAF values in the highest bin (0.9–1.0) were from 21–166 times greater than would be expected based on a random distribution of nesting sites (Table 5). Furthermore, in seven modeling regions, the highest RND bin was estimated to have more than twice the density of nest sites as the next lower bin (Table 5). A binary classification of the results would have resulted in a complete loss of this understanding of differences, which could have large consequences for conservation and management.

In the absence of designed, probabilistic nest surveys, the actual number of golden eagle nest sites within each region could not be estimated. Observed densities of golden eagle nest sites within RND bins in all modeling regions were consistent with nesting densities reported in the literature (e.g., [64, 28, 2]). Two important points need to be made about our observed golden eagle nest site densities within RND bins. First, they are based on a sample of thinned nest sites, and it is certain that those underestimate the actual number of independent golden eagle nest sites in modeling areas. Second, our density calculations assume contiguous areas of each RND bin, whereas the reality is that nearly all breeding sites contain mixtures of varying RND values. Few golden eagle pairs have uniformly contiguous territory-sized areas of high-value RND habitat available to them. Nonetheless, we believe the variation we estimated in AAF values among bins represents real variation, and the steep gradient between low and high RND bin values represents differences with real biological relevance.

We interpreted model results based on MaxEnt output as measures of relative nest site density. Royle et al. [67] criticized the interpretation of MaxEnt output as a habitat suitability index, in part because “it is not suitable for making explicit predictions of an actual state variable.” We believe our approach, based on modifying MaxEnt output by computing an AAF, transformed MaxEnt output into a biologically relevant state variable (relative density) that has a clear biological interpretation. Although AAF is proportional to density, it is more than a simple ranking of densities among RND bins because the magnitude of differences in density among bins can be explicitly estimated. Our modeling results provide direct measures of spatial variation in golden eagle nest site density and how this variation relates to spatial variation in environmental covariates. We believe our modeling approach is broadly applicable to other species and circumstances, including the modeling of presence-only data collected opportunistically.

The golden eagle nest data we compiled came from a broad range of survey efforts, including systematic statewide aerial surveys, local research projects, and landscape-scale surveys by land management agencies. However, we recognize several potential sources of bias in the data. For example, state and federally required surveys of public land targeted for oil, gas and coal extraction, as well as wind and solar energy development areas, often resulted in relatively high densities of golden eagle breeding sites recorded in those landscapes. Conversely, large expanses of privately owned ranch and farmland receive few surveys, as do remote wilderness areas. Even in surveyed areas, negative survey results are not always recorded into agency databases. To address potential spatial bias in the distribution of presence locations, Phillips et al. [54] recommended restricting the selection of available locations to a small neighborhood surrounding the presence locations. In our analyses, we sampled available locations within the union of a 20-km radius buffer (the modeling area) around all thinned golden eagle nest sites. Because golden eagle home ranges can be very large, and individuals often make periodic excursions <75 km outside of their home ranges in relatively short periods [52, 13], we assumed that each nesting pair could assess the 20-km radius area surrounding their breeding site.

Halvorsen [68] identified three motivations for fitting SDMs to presence-only data: 1) to estimate an ecological response, 2) for spatial predictions to unsampled locations, and 3) projective prediction modeling (i.e., projecting the model outside of the fitted area). Our models addressed all three goals, with an emphasis on spatial prediction. We chose to fit models using covariates supported by previous studies and current understanding of golden eagle nest site selection. This approach supported our primary focus on spatial prediction of golden eagle nesting density, while resulting in biologically interpretable models for use in conservation planning.

### Conservation applications

SDMs have broad applicability in conservation planning, but until recently have seldom been incorporated into on-the-ground conservation at ecosystem or regional scales [30]. Notable exceptions include habitat prioritization to support conservation of greater sage-grouse [69] and critical habitat designation for the northern spotted owl [3]. We developed our golden eagle nest site models with the goal of providing reliable planning and decision support tools to inform on-the-ground conservation actions.

When considering golden eagle conservation in conjunction with other land-use decisions, our models offer the critical insight that while deleterious impacts on a small percentage of each modeling region could have disproportionately large population-level consequences to eagles, there is also the opportunity for spatially efficient conservation actions that disproportionately benefit golden eagles. Application of the models to specific golden eagle management-related questions requires a thorough understanding of model characteristics, including limitations, appropriate scales for drawing inference, and in some instances, federal policies and guidance governing implementation of the Eagle Act.

When projected as maps depicting relative density of golden eagle nest sites, our models provide a regional- and landscape-scale analysis tool for proactive planning of development projects that potentially impact eagles. This approach is valuable for a variety of land-use activities ranging from planning recreational vehicle trails on public lands to energy infrastructure projects, most notably siting of wind energy development [70, 45]. Vulnerability of golden eagles to collision with wind turbines, coupled with the Eagle Act’s prohibition of unauthorized incidental ‘take’ of eagles and subsequent legal risk to energy companies, provide an incentive for proactive risk reduction through appropriate project siting. Our models are ideally suited for landscape-scale ‘desktop’ analyses of potential eagle exposure described in federal guidance for wind energy development (Stage 1 Landscape-scale Site Assessment [71]), as an initial step in assessing and comparing potential development areas. The cost and time investment for landscape-scale surveys to support these assessments can be prohibitively costly and time-consuming, making use of a model-based framework an effective and efficient means to identify areas of relatively high risk (eagle exposure) for more detailed study or targeted field surveys. Recognizing that golden eagles are among many biological, economic and social considerations that influence the decision-making process during ‘prospecting’ by wind energy developers [72], we encourage integration of our models into existing landscape analysis tools such as the Western Association of Fish and Wildlife Agencies’ Crucial Habitat Assessment Tool (CHAT; https://www.wafwa.org/initiatives/crucial_habitat_assessment_tool/), The Nature Conservancy’s Site Wind Right program (https://Nature.org/sitewindright), and the National Renewable Energy Laboratory’s Wind Prospector toolkit (https://maps.nrel.gov/wind-prospector/).

Our models can be used to integrate golden eagle breeding habitat into ecosystem- or regional-scale conservation planning efforts, such as sagebrush and greater sage-grouse initiatives (https://www.wafwa.org/initiatives/sagebrush_ecosystem_initiative/). The roughly 1.8 million km^2^ sagebrush biome encompasses 50% of the golden eagle’s range in the western contiguous US, and 79% of the nest sites available for our modeling effort. Golden eagles and greater sage-grouse face many of the same threats in the sagebrush biome (e.g., wildfire, development, agricultural conversion, and other forms of loss or fragmentation of sagebrush [73, 74]), and there is a high degree of overlap in high quality breeding habitat for both species [75]. Thus, conservation efforts aimed at ameliorating threats in the sagebrush biome are likely to benefit both species [22, 75]) and other sagebrush-associated species of conservation concern [76, 77]. For example, landscape-scale removal of western juniper (*Juniperus occidentalis*) to improve habitat conditions for sage-grouse [78, 79] would be anticipated to also benefit golden eagle foraging habitat and important prey such as cottontail rabbits (*Sylvilagus* spp. [80]), but could have negative impacts on eagles if implemented in high-quality nesting habitat where junipers in steep terrain are used as nest sites.

Our RND models provide a spatially explicit measure of golden eagle abundance (exposure) which combined with indices of various hazards in a formal risk analysis framework can identify priority areas for conservation action and mitigation. Bedrosian et al. [27] combined our Northwestern Plains model with a spatial model of power pole density (a surrogate for electrocution hazard [81]) to create a spatially explicit index of electrocution risk. Such risk maps provide decision support tools to guide efficient allocation of conservation resources (e.g., power pole retrofitting) to reduce take of golden eagles or as targeted compensatory mitigation to offset permitted take of golden eagles occurring elsewhere[19].

SDMs are an important component of spatially explicit population models, which are increasingly popular for forecasting population responses to changes in climate, habitat, and anthropogenic stressors [82, 83]. Wiens et al. [2] combined an SDM of golden eagle nesting habitat suitability with spatial data on eagle prey abundance and potential risk factors (e.g., wind turbines, roads, and powerlines) in a spatial demographic model to simulate eagle population responses to proposed energy development scenarios in California. Because our nest site density models are based on relative habitat suitability, they can serve as spatial layers in similar demographic models to evaluate relative effectiveness of golden eagle conservation scenarios at regional or larger scales.

The appropriateness of our models for conservation applications is constrained by the spatial scale at which the models are applied. The base resolution of our models is 120-m (1.44-ha) pixels, but evaluation of, at least, thousands of pixels is necessary before meaningful comparisons of relative nest density can be made. Our models are intended for application at regional, landscape, and project assessment area scales (i.e., from thousands to millions of ha); and should not be used at the scale of small projects (hundreds of ha or less). From an ecological perspective, it is important to recognize that our models represent only golden eagles occupying breeding sites, not the full range of habitats used by non-breeding, wintering, or migrating eagles.

Relative density models do not represent estimates of probability of occurrence or absolute abundance. Although our relative density models can estimate the magnitude of difference in nesting golden eagle densities among various areas, they do not estimate density per-se. We caution against using our models by themselves for estimating actual (as opposed to relative) abundance of eagles for use in (for example) estimating eagle exposure in a collision risk model to predict collision fatalities at wind energy facilities [71, 84], or estimation of mortality offset by a proposed compensatory mitigation project. Given an estimated or hypothetical number of nest sites or territories within a landscape or region, however, our models can be used to predict how those nest sites would be distributed.

Because we developed models separately for each modeling region, relative nest density values (RND and AAF) from individual regions are not directly comparable among regions. Each model’s results are relative only to other areas within the same region. For larger-scale (e.g., state- or west-wide) conservation planning, standardization among regional models could be accomplished using the area-adjusted frequency of training data (naïve density).

Modeling to support conservation planning is, in our view, an iterative process; models should be refined when improved data become available. In the course of compiling and analyzing golden eagle nest site data, we identified areas with few surveys or nest records for model training and in some cases (California Foothills, Columbia Plateau, and Southwestern Plains) developed models that, although useful, would likely be improved with a larger, more spatially balanced training sample of nest sites.

## Supporting information

S1 Fig Maps of modeling areas for twelve modeling regions of the western United States. (PDF)

S2 Fig Maps of evaluation sub-regions for twelve modeling regions of the western United States. (PDF)

S3 Fig Functional forms of model covariates for twelve modeling regions of the western United States. (PDF)

S4 Fig Maps of golden eagle relative nest site density (RND) for twelve modeling regions of the western United States. (PDF)

S1 Table Data sources for all covariates. (XLSX)

S2 Table Percentage of each (A) modeling area and (B) modeling region composed of each of 10 RND classes/bins based on each modeling regions’ final model.
(PDF)

S3 Table Acknowledgment of individuals and institutions who contributed data and valuable input to this project. (PDF)
