# Supplementary material for: Modeling spatial variation in density of golden eagle nest sites in the western United States
Source: PLoS One. 2019 Sep 30;14(9):e0223143. doi: 10.1371/journal.pone.0223143 (PMC6768475; doi:10.1371/journal.pone.0223143)
Supplement: S1 Fig — (PDF) [file pone.0223143.s001.pdf]

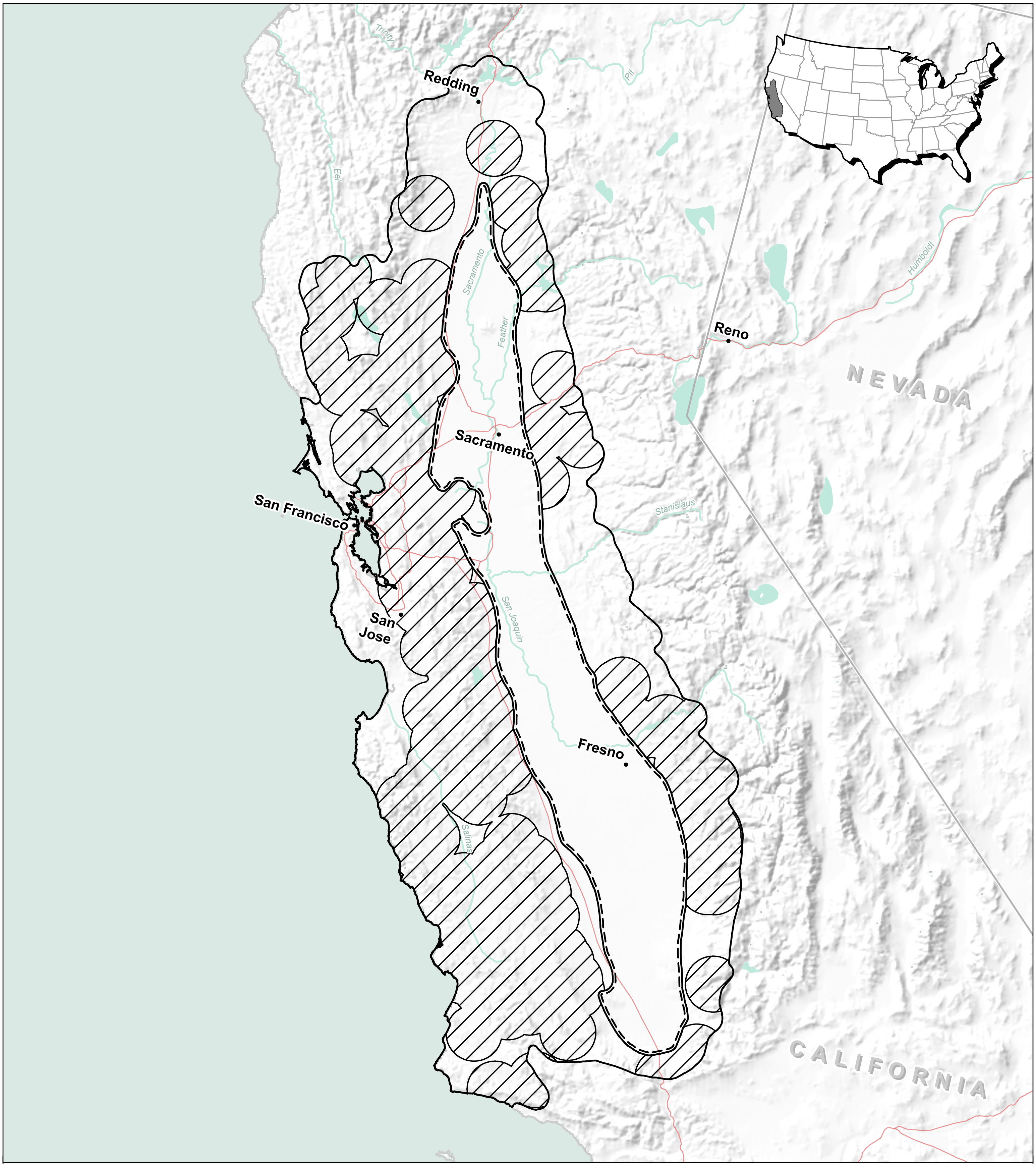

Golden Eagle Nest Site Model

Model Region and Modeling Areas

California Foothills & California Central Valley

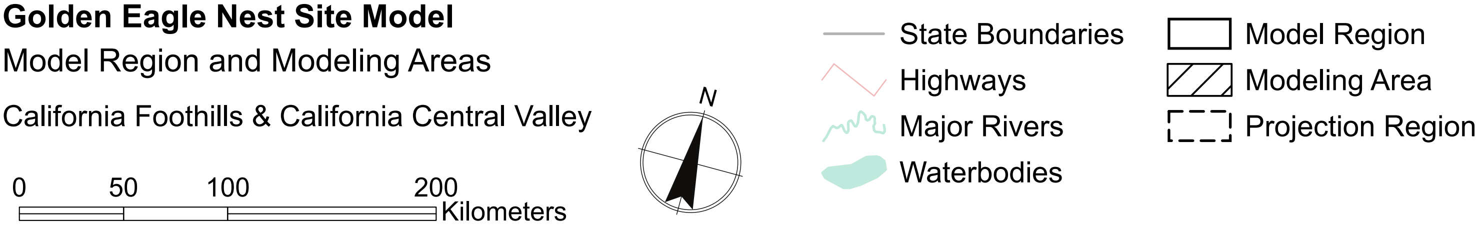

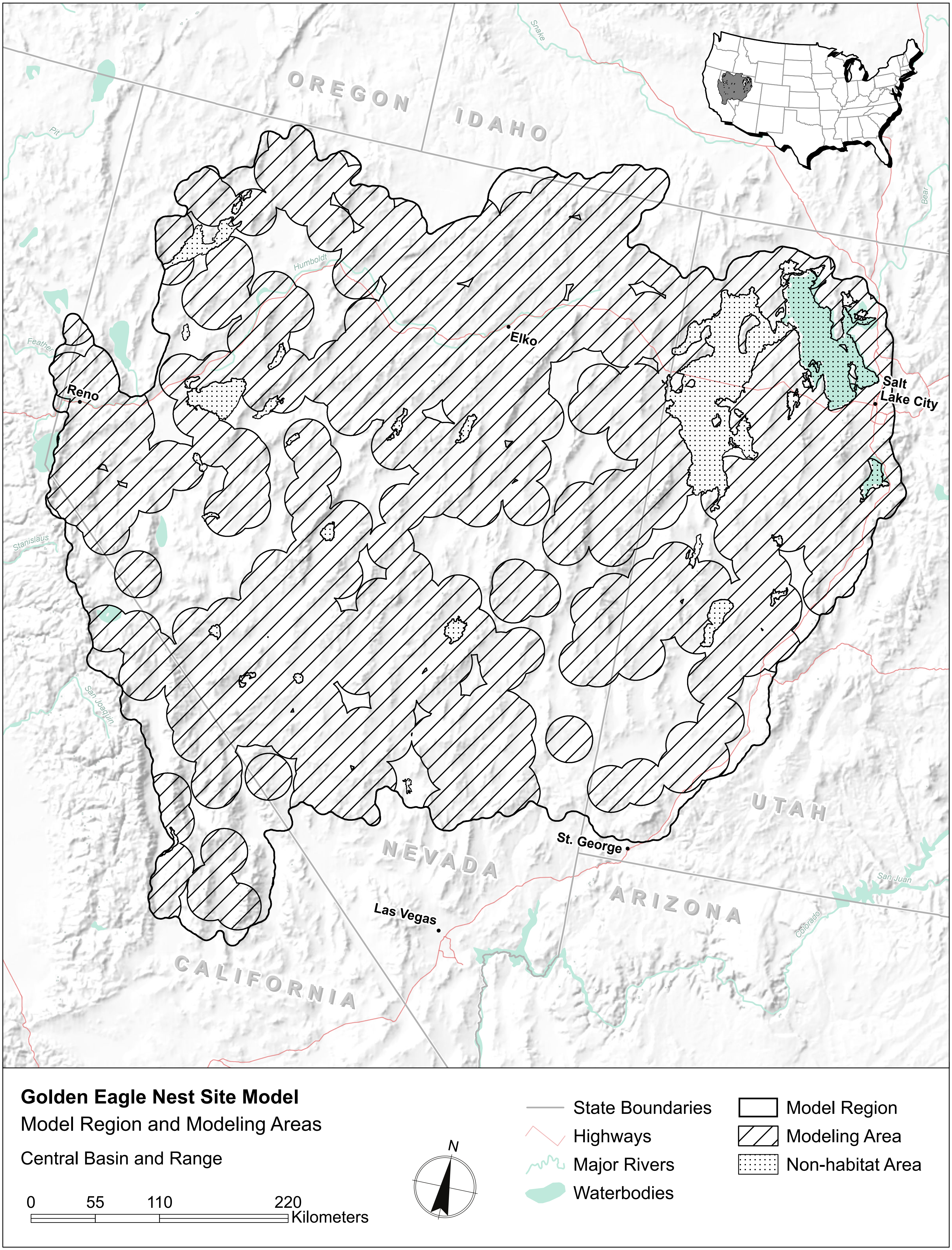

Golden Eagle Nest Site Model

Model Region and Modeling Areas

Central Basin and Range

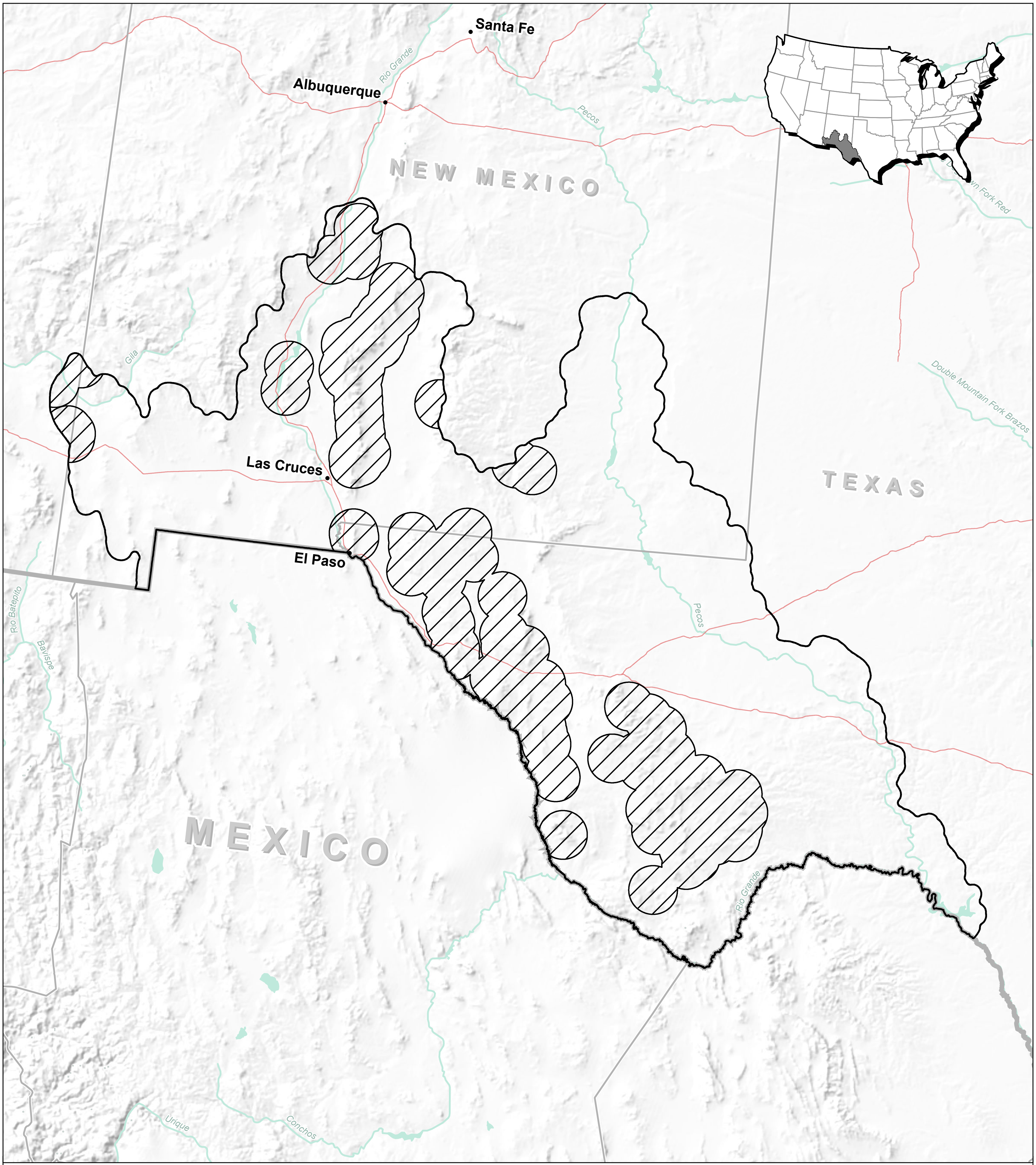

Golden Eagle Nest Site Model

Model Region and Modeling Areas

Chihuahuan Desert

0 55 110 220 Kilometers

N

State Boundaries
Highways
Major Rivers
Waterbodies
Model Region
Modeling Area

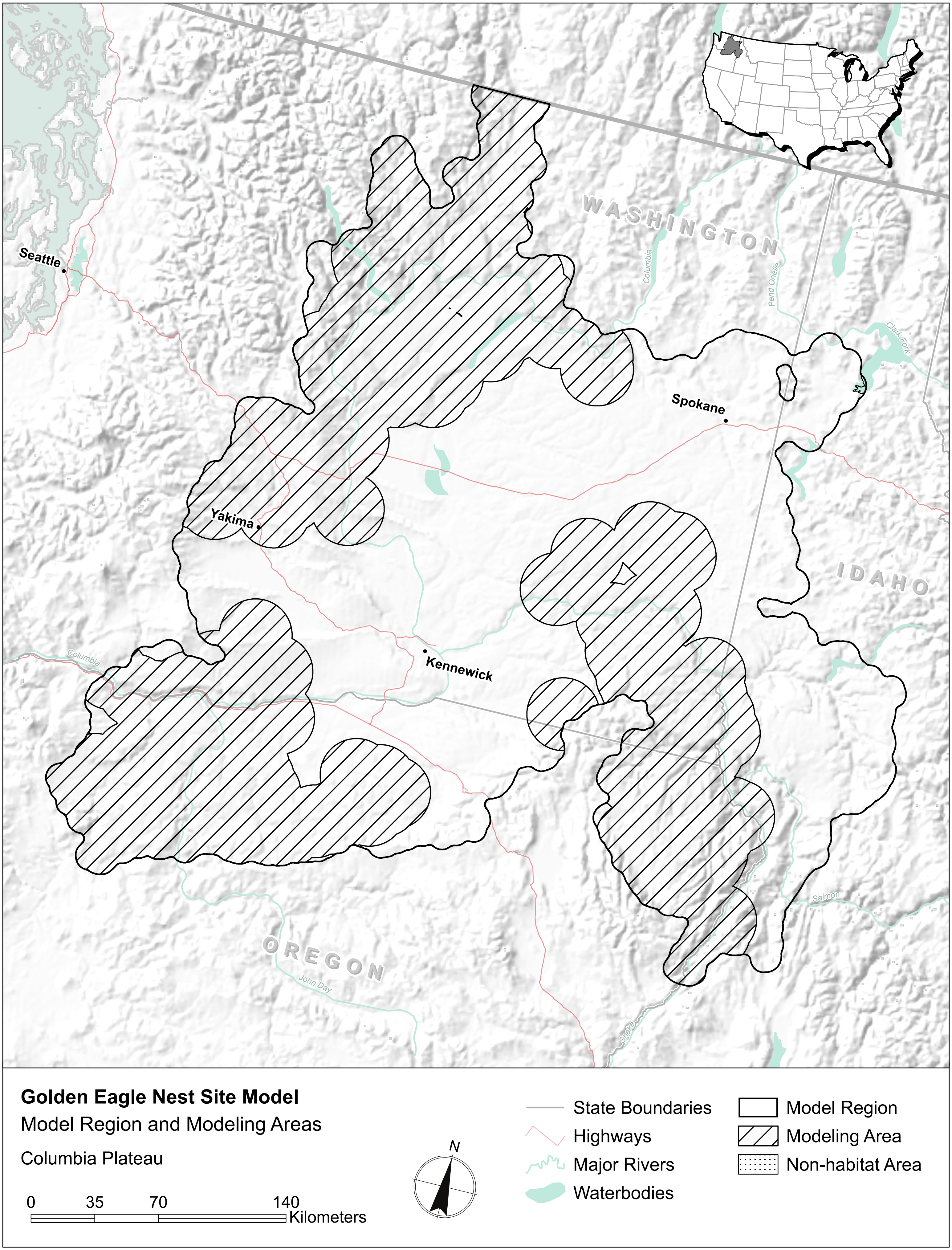

Seattle
Yakima
Kennewick
Spokane
WASHINGTON
IDAHO
OREGON
Columbia
Pend Oreille
Clark Fork
Salmon
John Day
Snake
Golden Eagle Nest Site Model
Model Region and Modeling Areas
Columbia Plateau
0
35
70
140
Kilometers
N
State Boundaries
Highways
Major Rivers
Waterbodies
Model Region
Modeling Area
Non-habitat Area

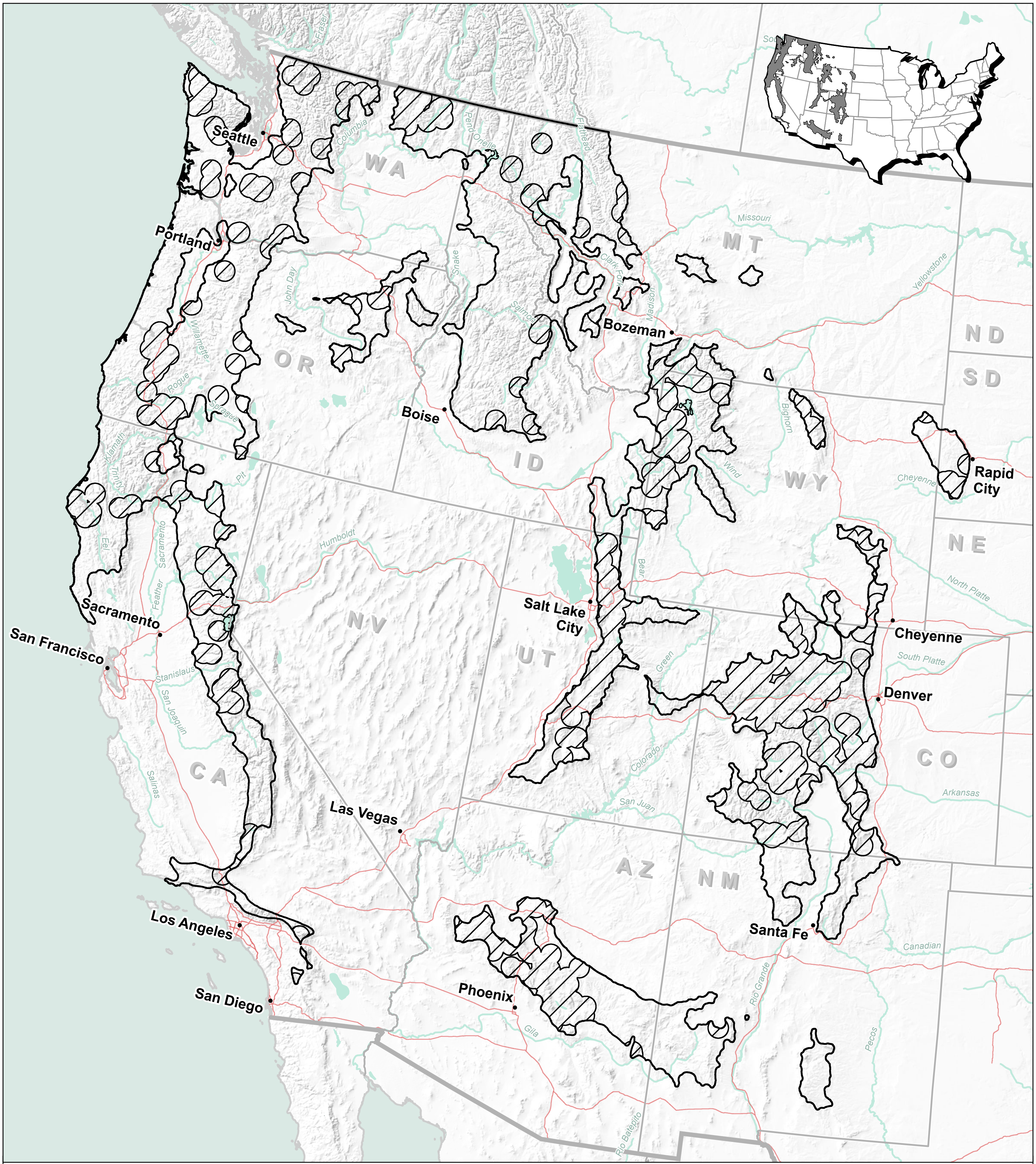

Golden Eagle Nest Site Model

Model Region and Modeling Areas

Forested Montane

0 140 280 560 Kilometers

N

State Boundaries
Highways
Major Rivers
Waterbodies

Model Region
Modeling Area
Non-habitat Area

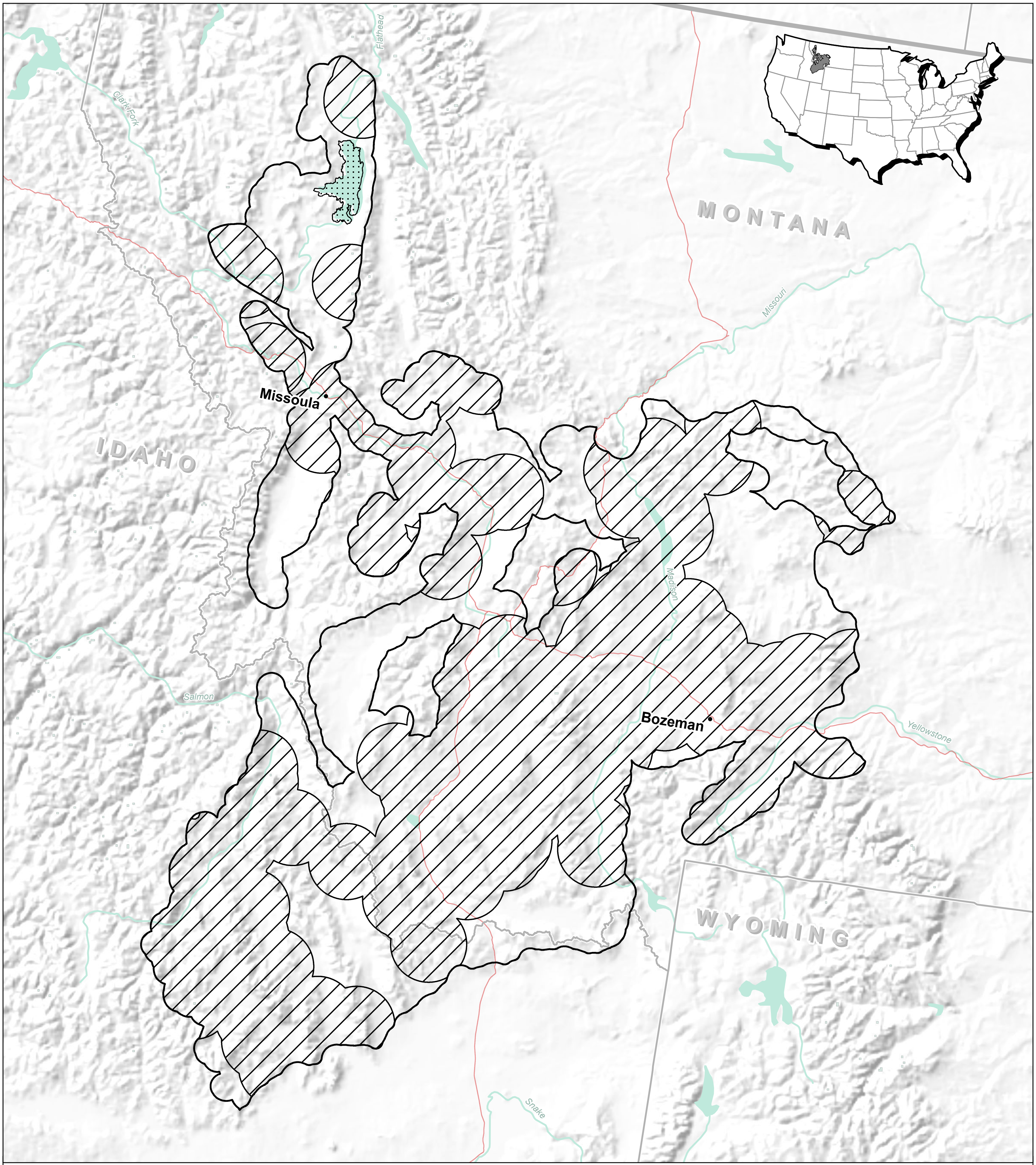

Golden Eagle Nest Site Model

Model Region and Modeling Areas

Intermontane Basins and Valleys

0 37.5 75 150 Kilometers

N

State Boundaries
Highways
Major Rivers
Waterbodies
Model Region
Modeling Area
Non-habitat Area

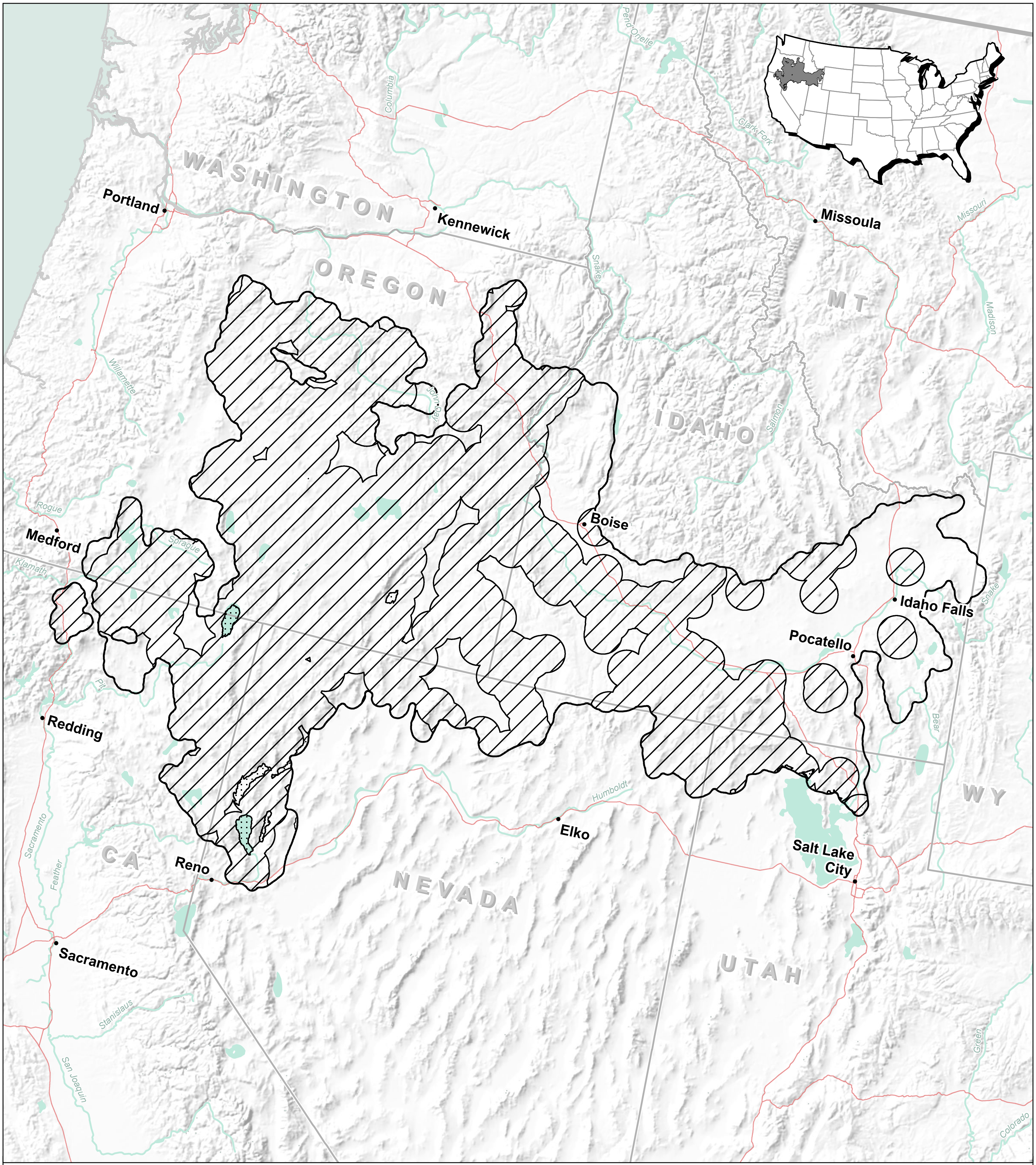

Golden Eagle Nest Site Model

Model Region and Modeling Areas

Northern Great Basin

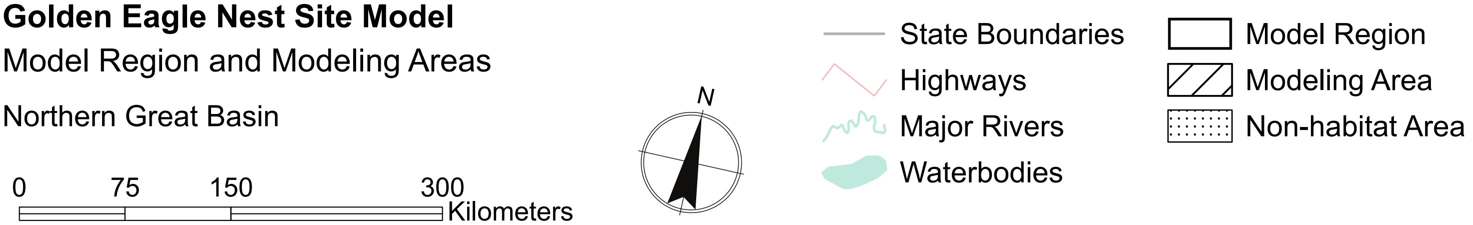

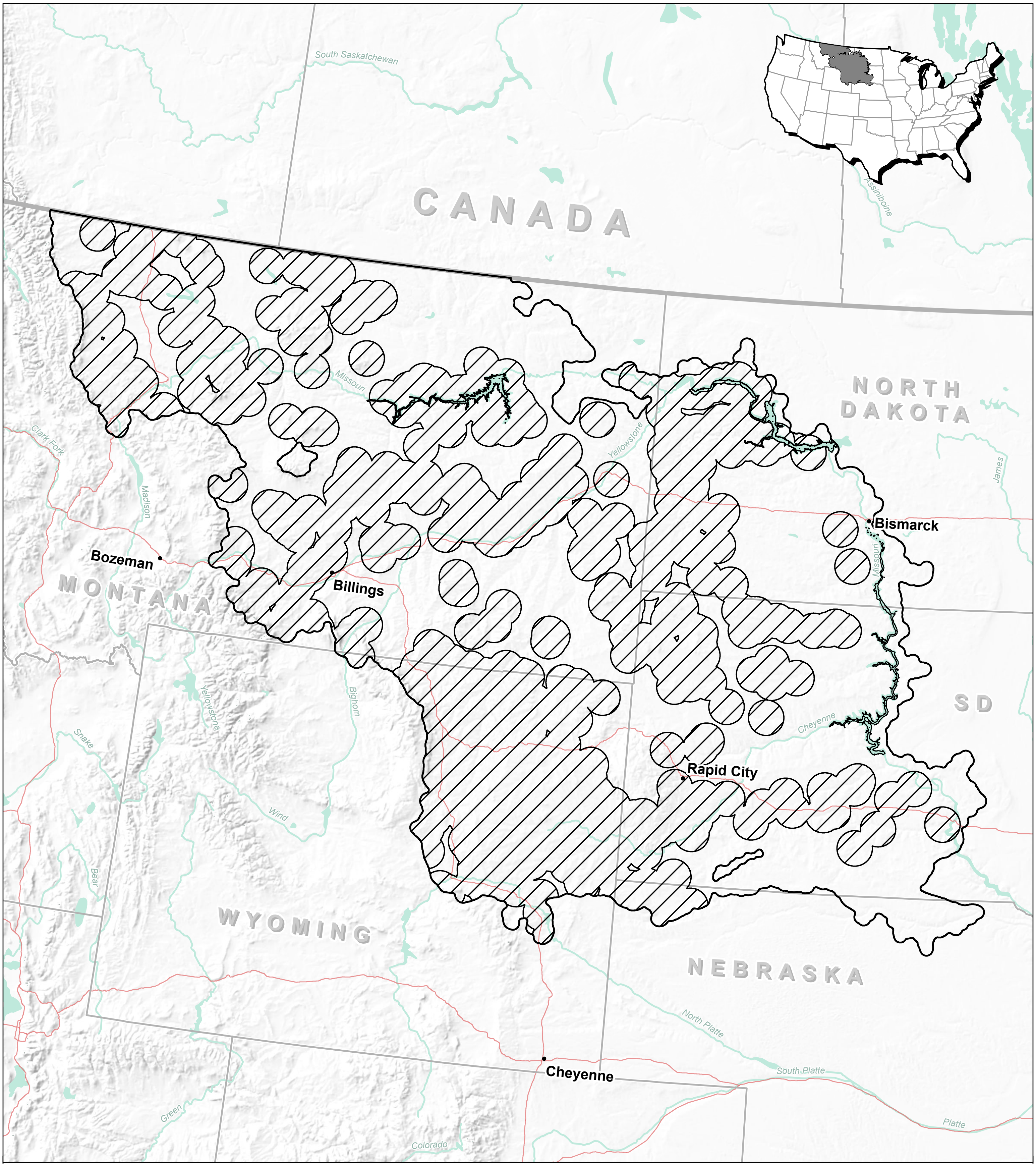

Golden Eagle Nest Site Model

Model Region and Modeling Areas

Northwestern Plains

0 80 160 320 Kilometers

N

State Boundaries
Highways
Major Rivers
Waterbodies

Model Region
Modeling Area
Non-habitat Area

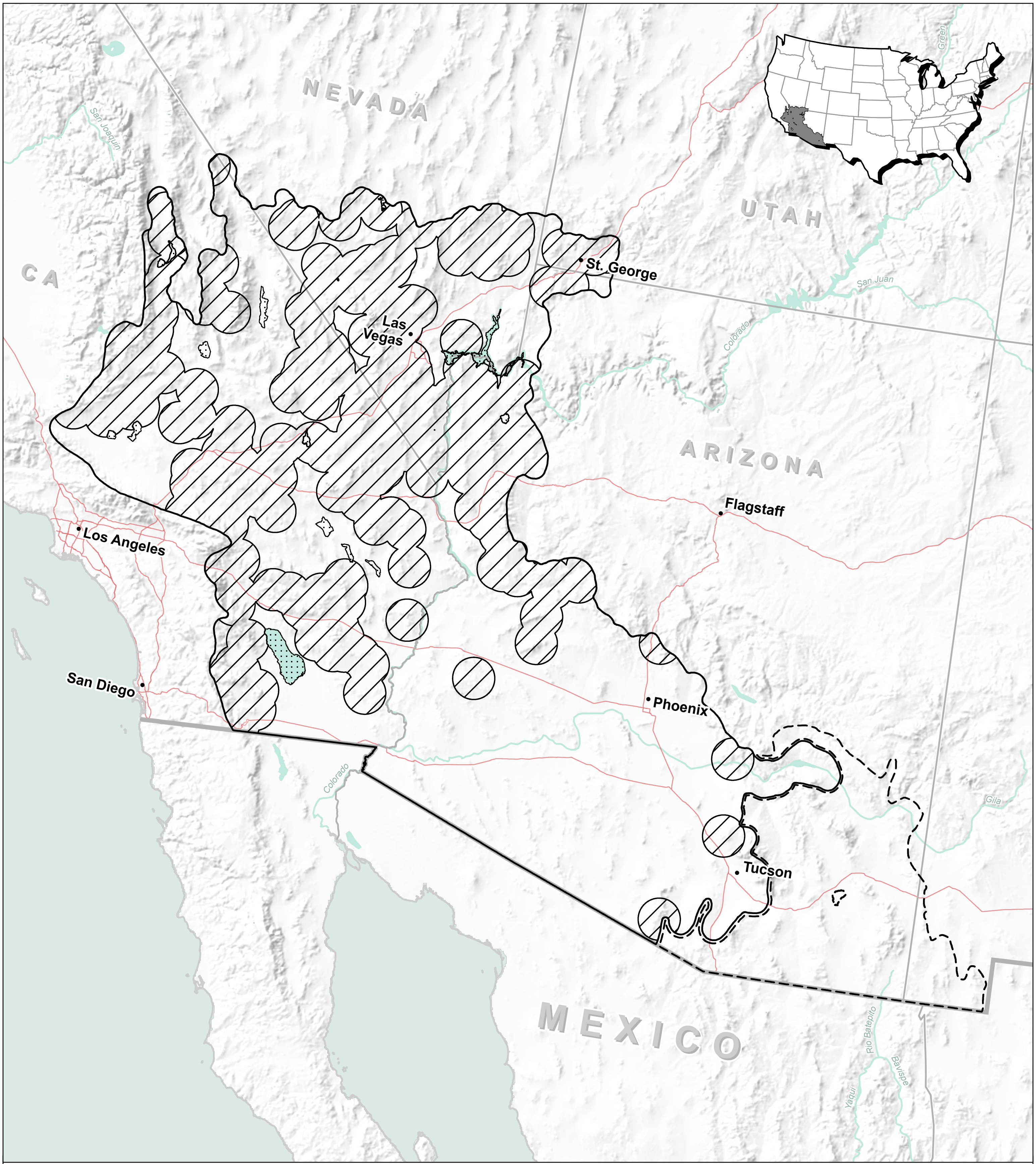

Golden Eagle Nest Site Model

Model Region and Modeling Areas

Southwestern Deserts & Madrean Archipelago

0 65 130 260 Kilometers

State Boundaries
Highways
Major Rivers
Waterbodies
Model Region
Modeling Area
Projection Region
Non-habitat Area

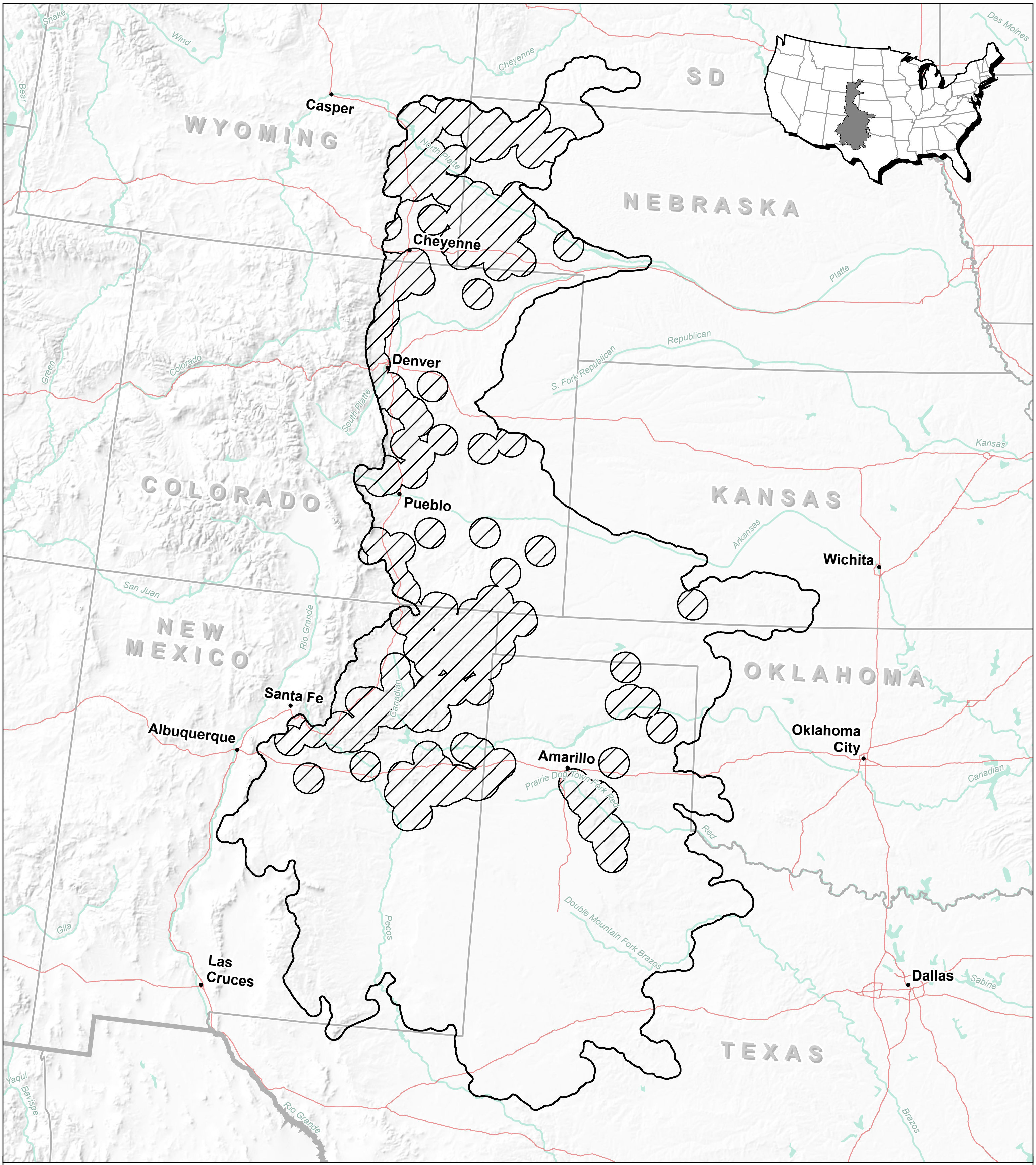

Golden Eagle Nest Site Model

Model Region and Modeling Areas

Southwestern Plains

0 95 190 380 Kilometers

State Boundaries

Highways

Major Rivers

Waterbodies

Model Region

Modeling Area

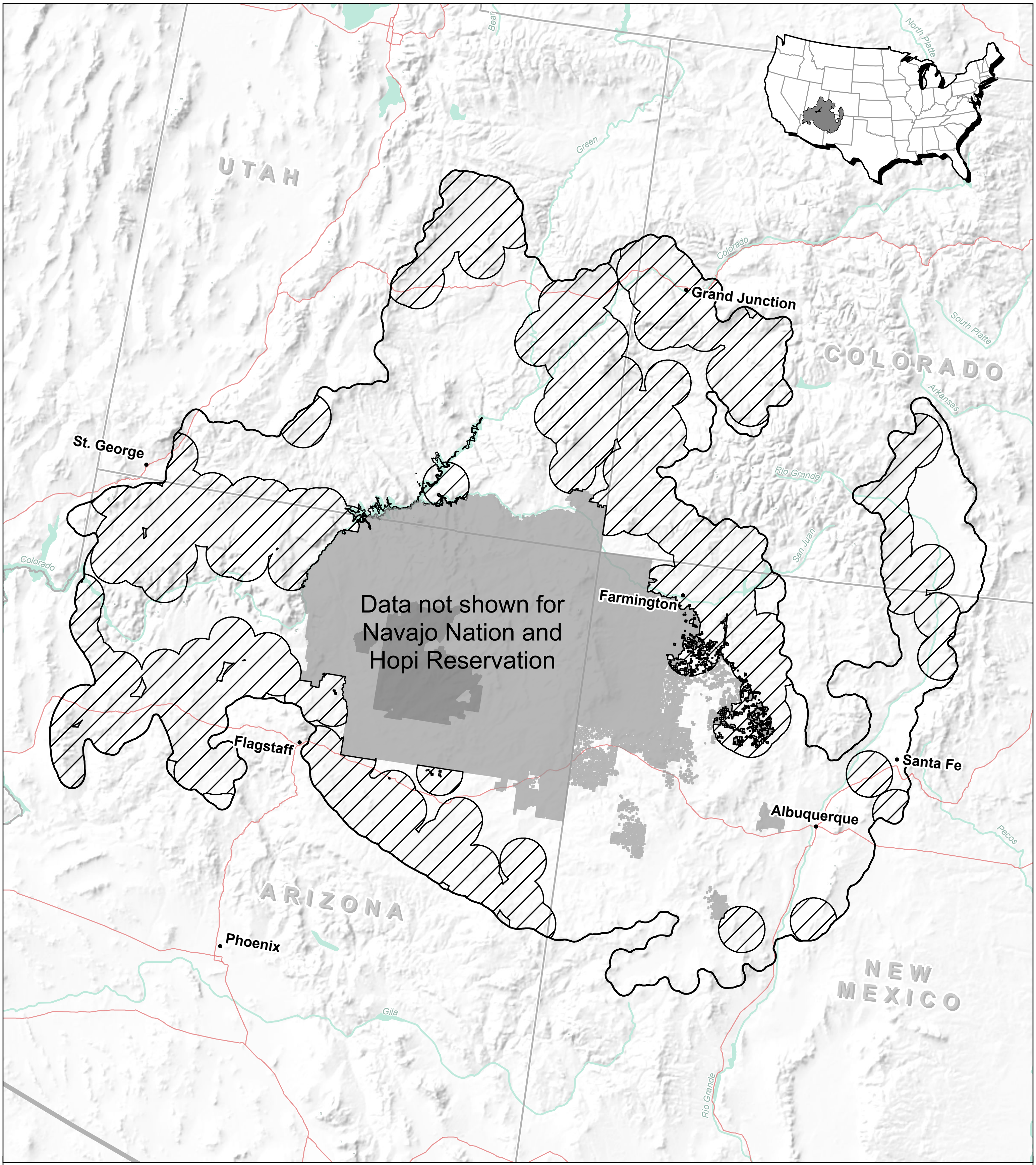

Golden Eagle Nest Site Model

Model Region and Modeling Areas

Southwestern Plateaus

0 62.5 125 250 Kilometers

N

State Boundaries
Highways
Major Rivers
Waterbodies
Model Region
Modeling Area
Non-habitat Area

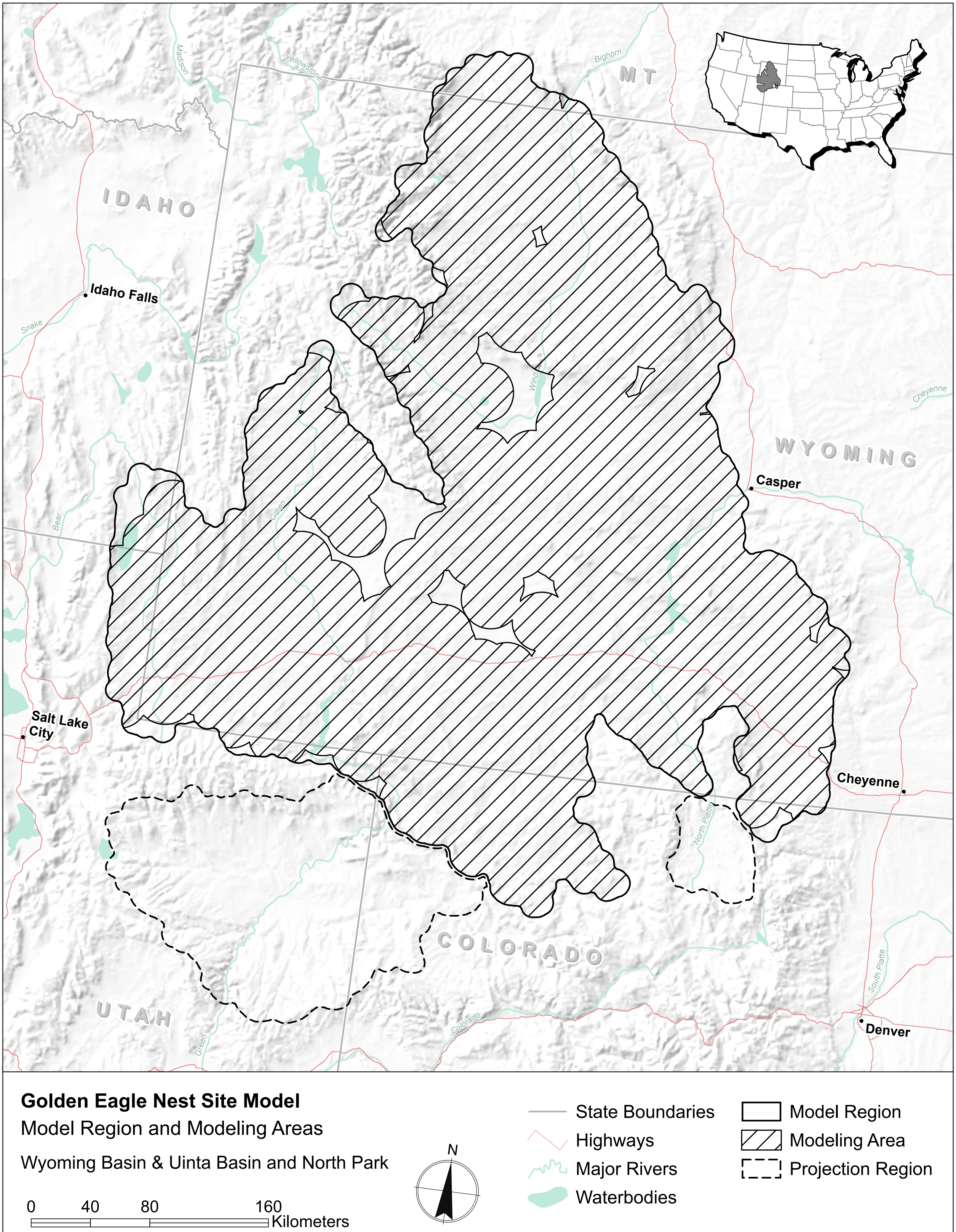

Golden Eagle Nest Site Model

Model Region and Modeling Areas

Wyoming Basin & Uinta Basin and North Park

State Boundaries
Highways
Major Rivers
Waterbodies
Model Region
Modeling Area
Projection Region
