# Supplementary material for: Modeling spatial variation in density of golden eagle nest sites in the western United States
Source: PLoS One. 2019 Sep 30;14(9):e0223143. doi: 10.1371/journal.pone.0223143 (PMC6768475; doi:10.1371/journal.pone.0223143)
Supplement: S2 Fig — (PDF) [file pone.0223143.s002.pdf]

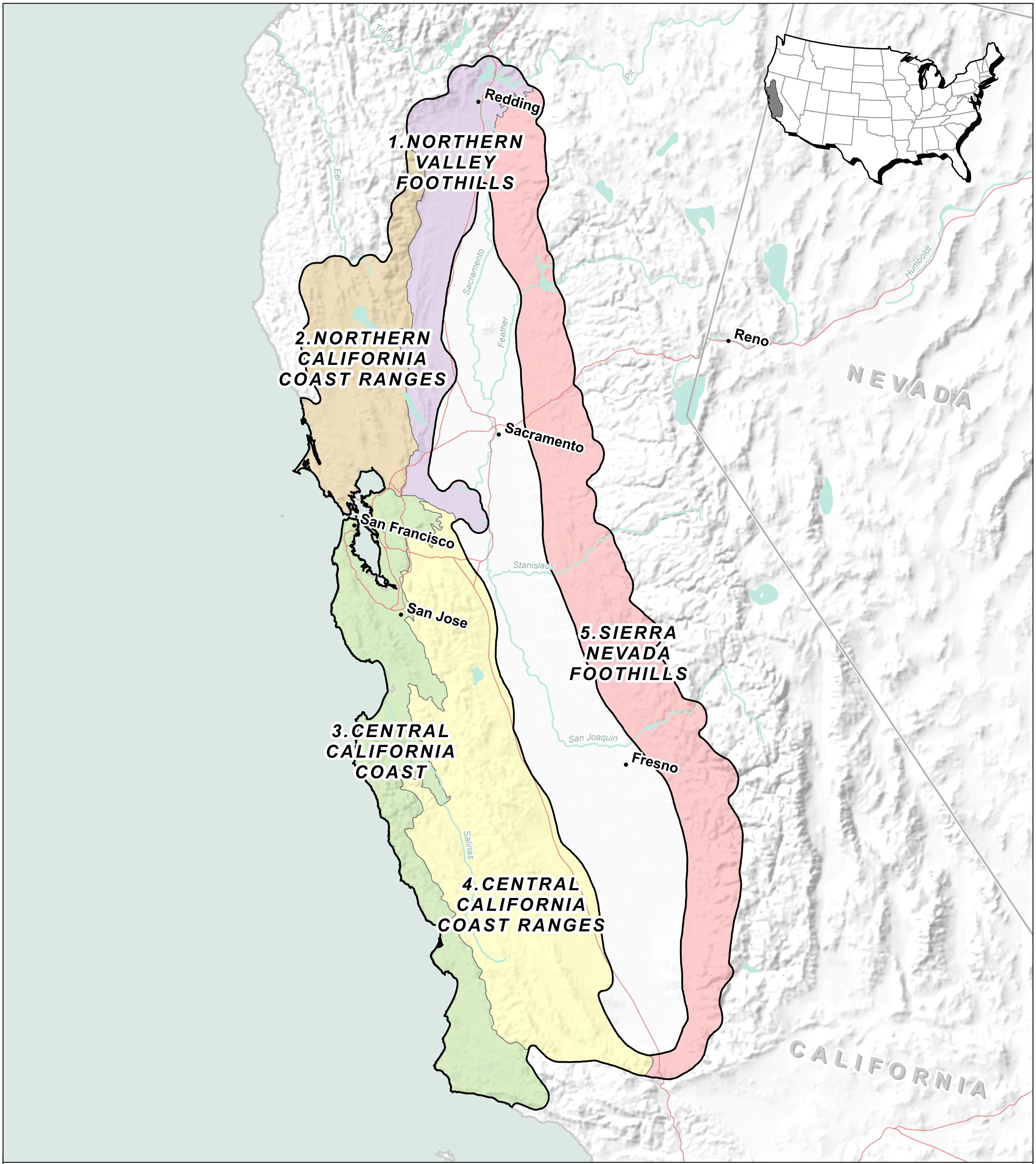

Golden Eagle Nest Site Model

Model Subregions

California Foothills & California Central Valley

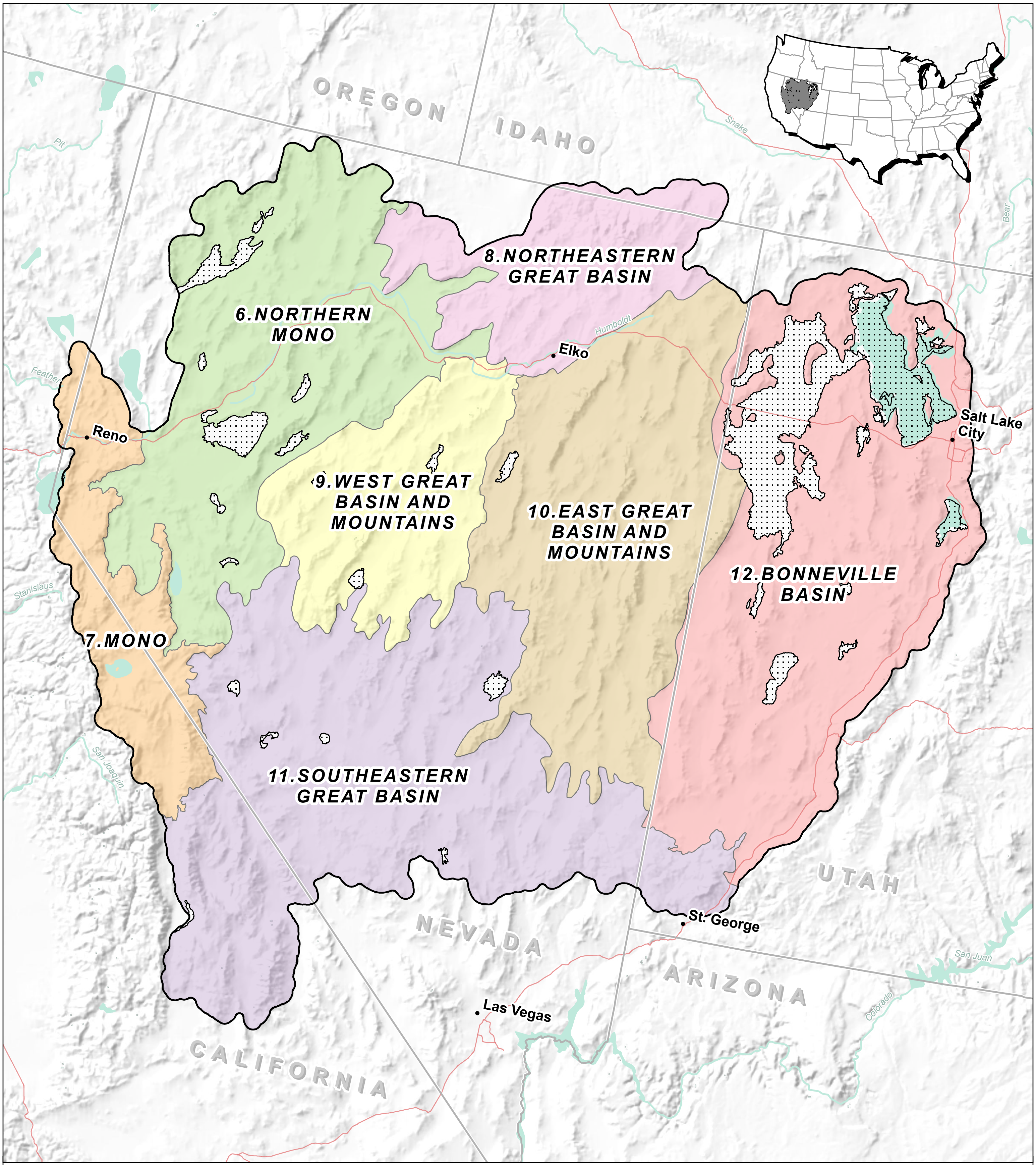

Golden Eagle Nest Site Model

Model Subregions

Central Basin and Range

0 55 110 220 Kilometers

N

State Boundaries
Highways
Major Rivers
Waterbodies
Model Region
Model Subregion
Non-habitat Area

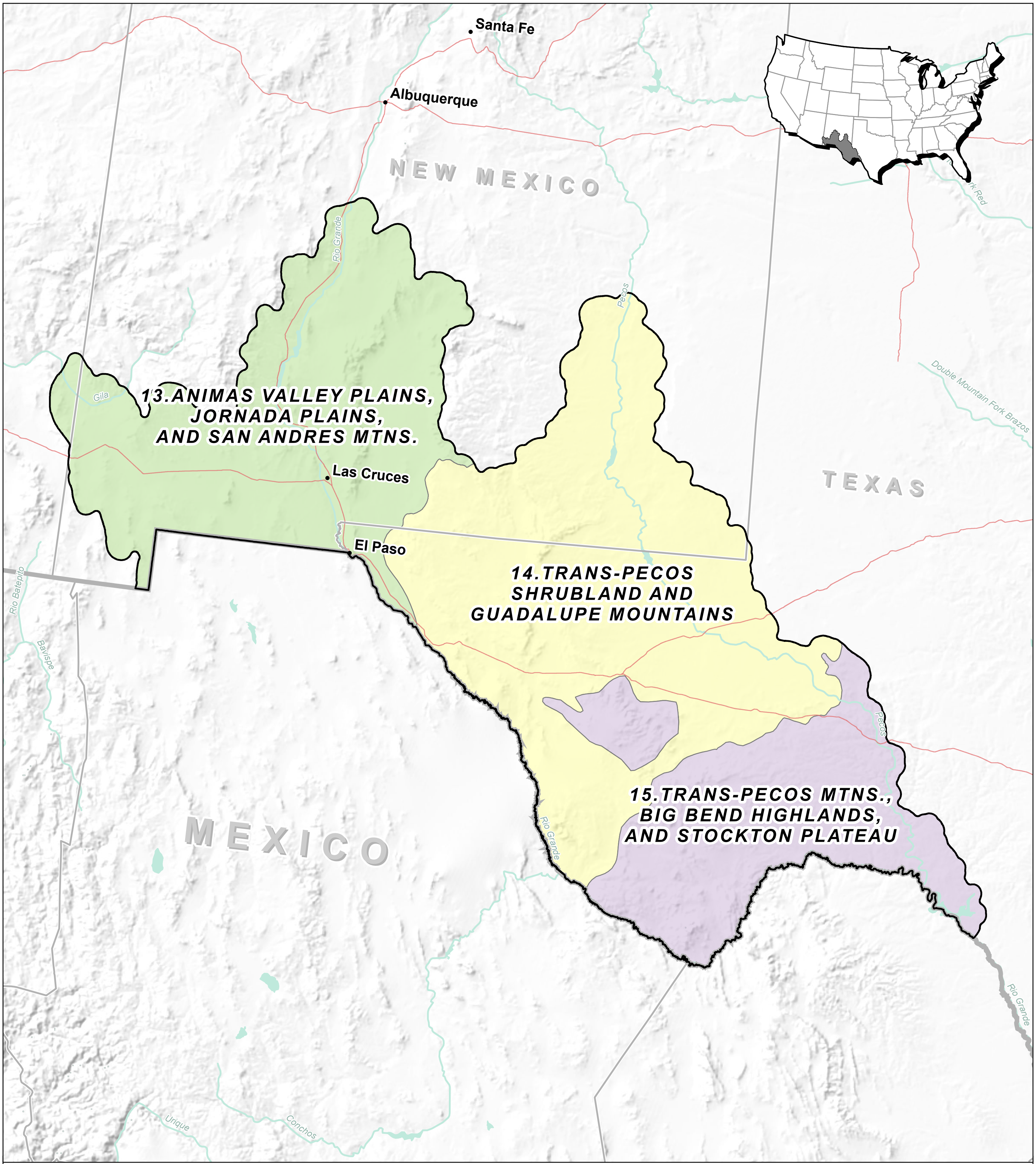

Golden Eagle Nest Site Model

Model Subregions

Chihuahuan Desert

0 55 110 220 Kilometers

N

State Boundaries
Highways
Major Rivers
Waterbodies
Model Region
Model Subregion

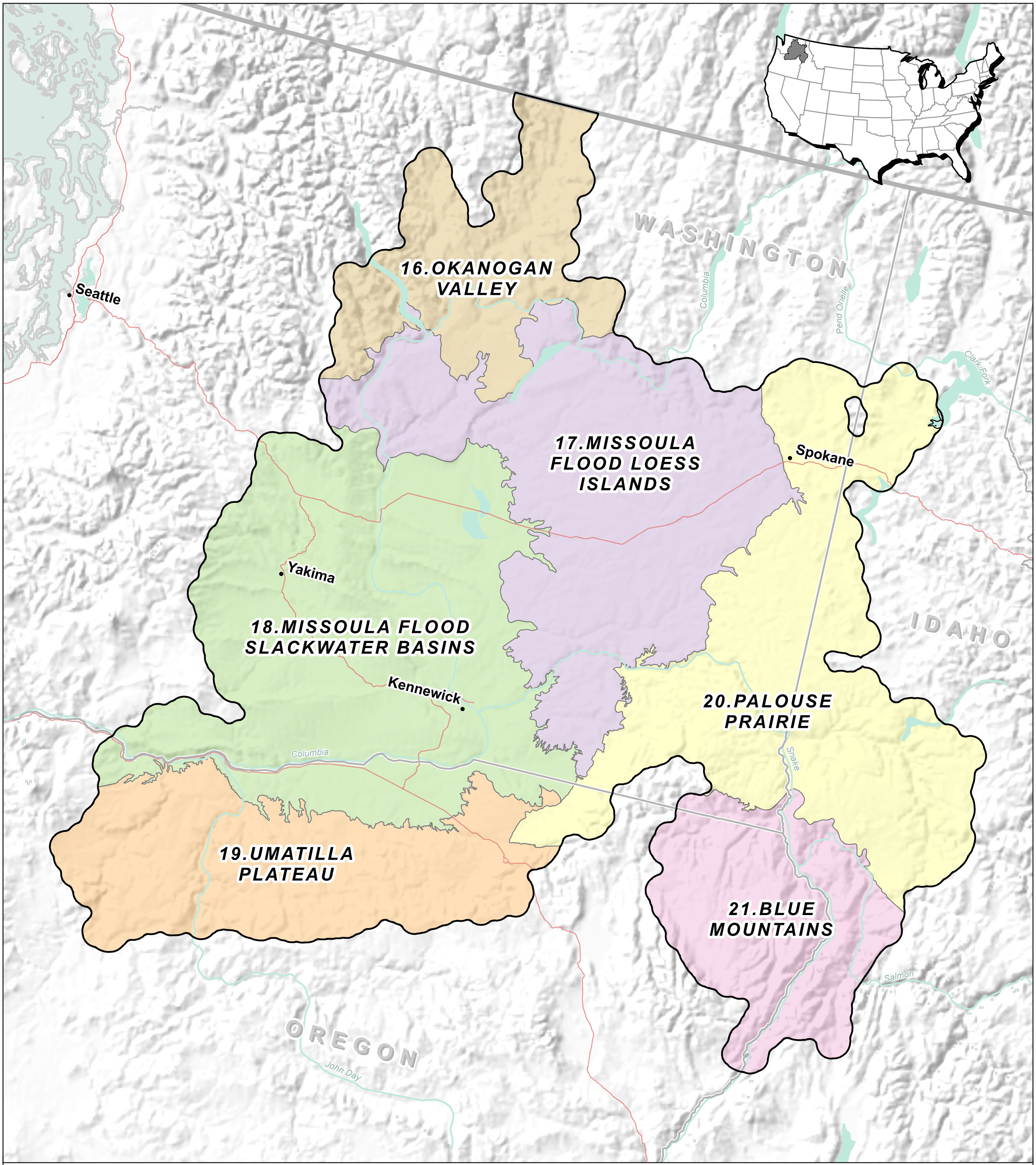

Golden Eagle Nest Site Model

Model Subregions

Columbia Plateau

0 35 70 140 Kilometers

N

State Boundaries

Highways

Major Rivers

Waterbodies

Model Region

Model Subregion

Non-habitat Area

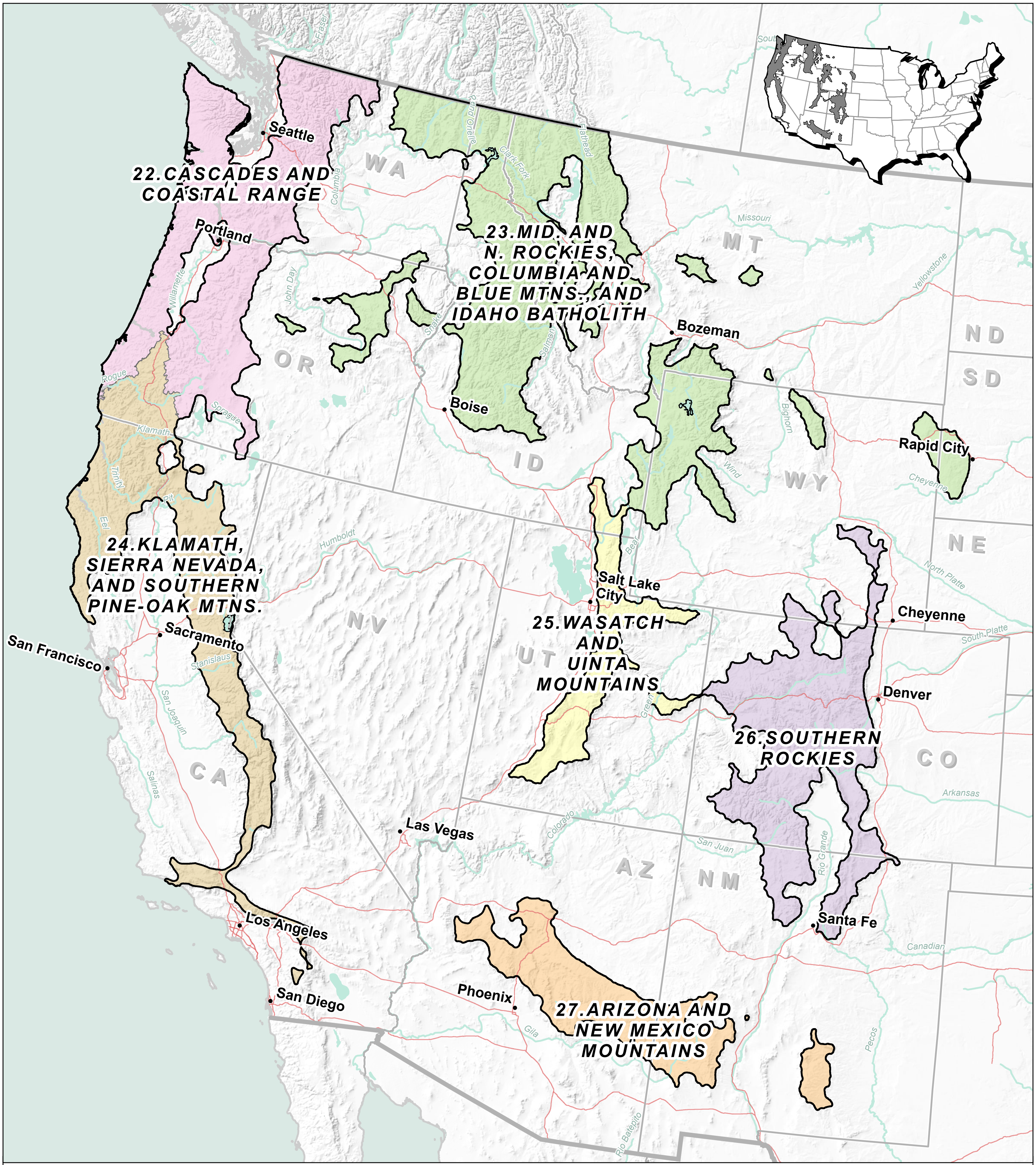

Golden Eagle Nest Site Model

Model Subregions

Forested Montane

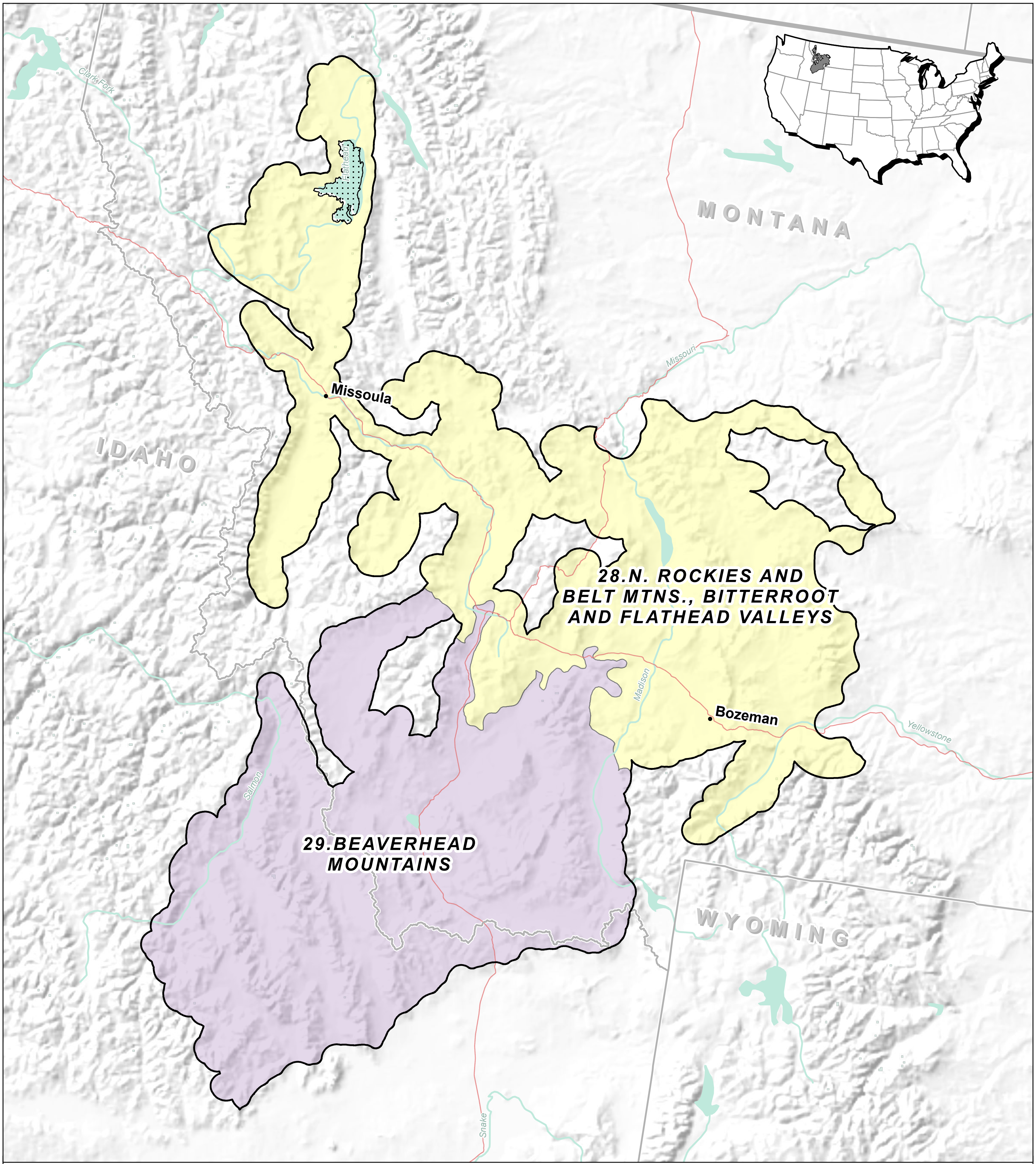

Golden Eagle Nest Site Model

Model Subregions

Intermontane Basins and Valleys

0 37.5 75 150 Kilometers

N

State Boundaries
Highways
Major Rivers
Waterbodies

Model Region
Model Subregion
Non-habitat Area

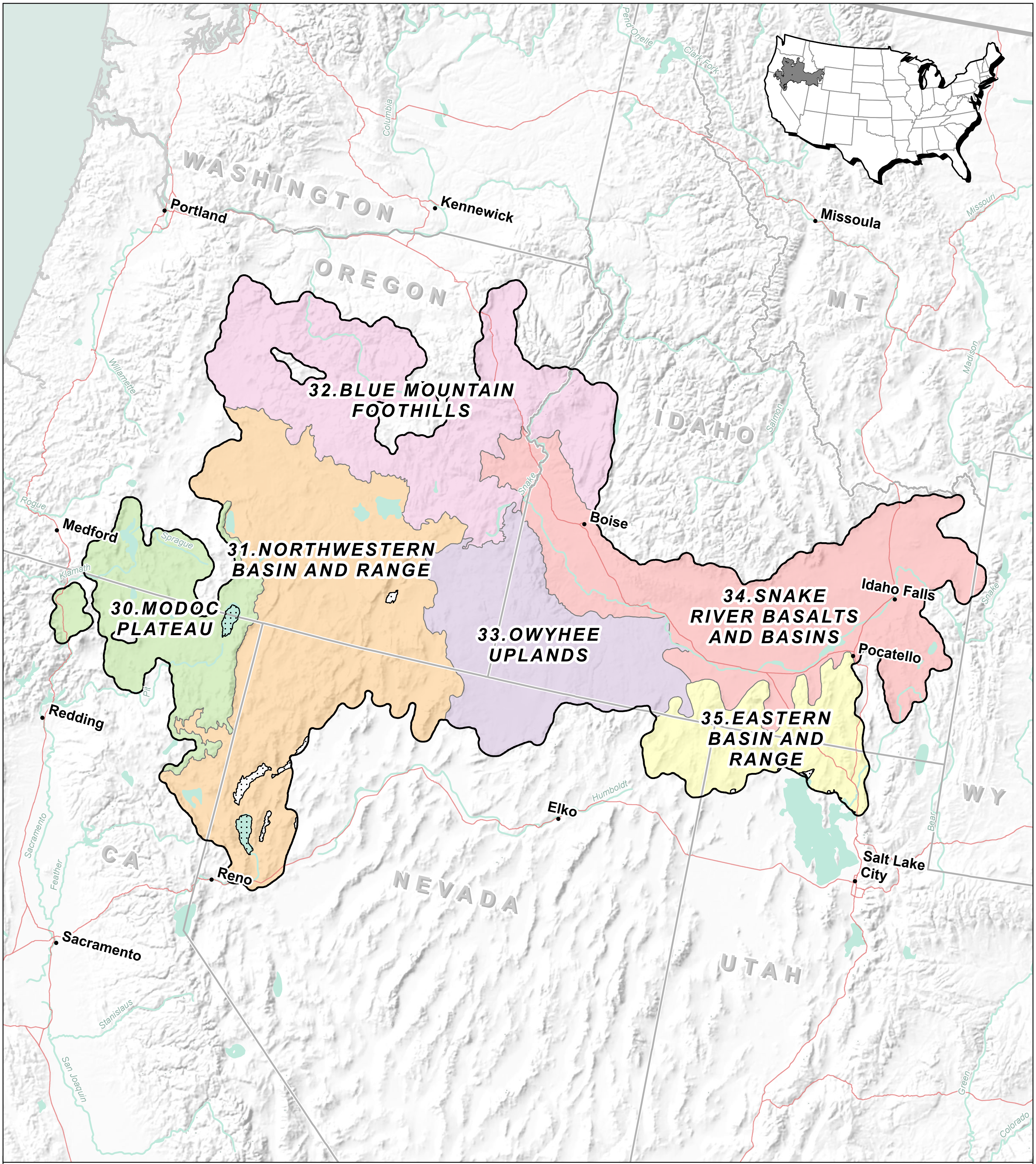

Golden Eagle Nest Site Model

Model Subregions

Northern Great Basin

0 75 150 300 Kilometers

N

State Boundaries
Highways
Major Rivers
Waterbodies
Model Region
Model Subregion
Non-habitat Area

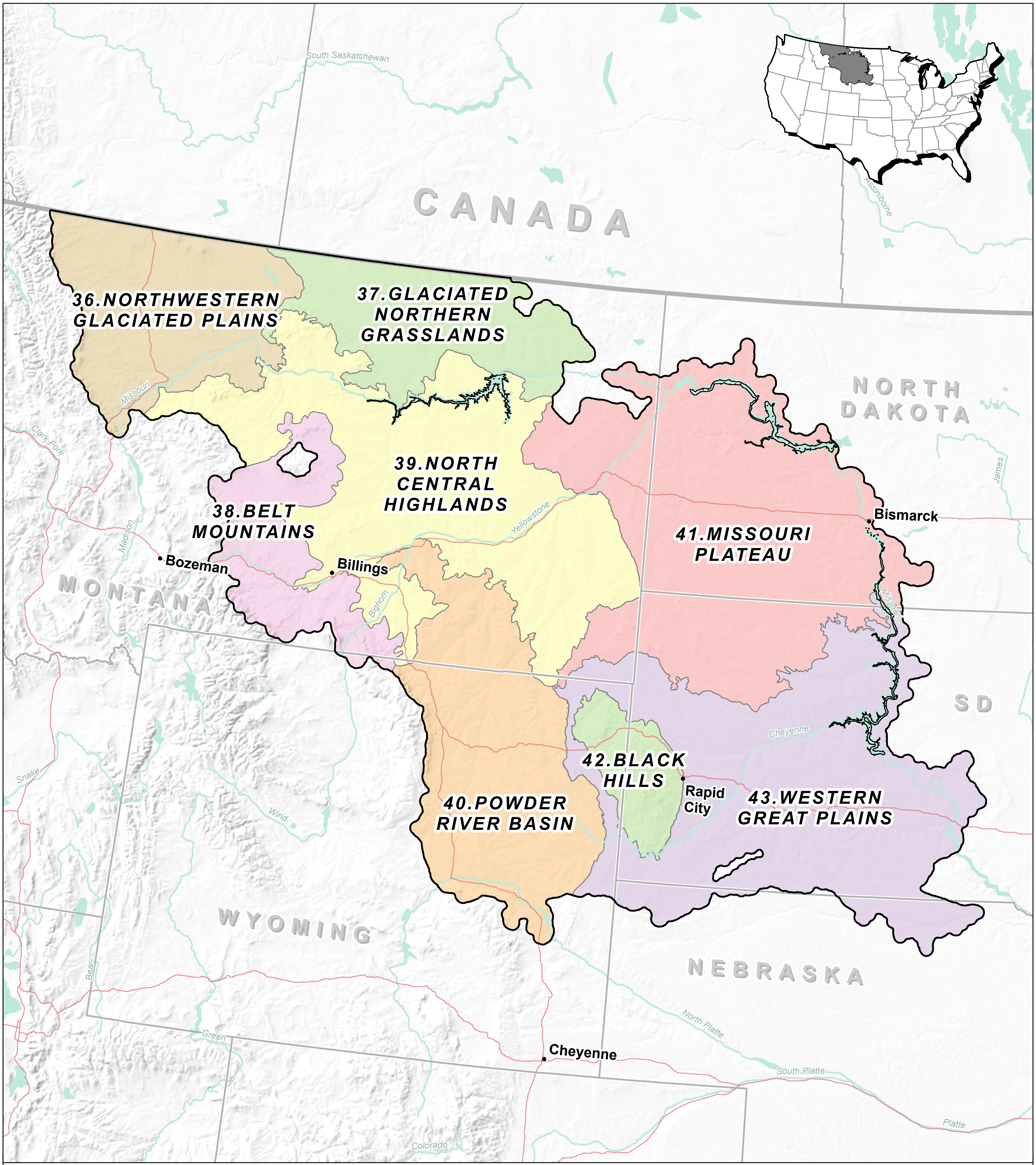

Golden Eagle Nest Site Model

Model Subregions

Northwestern Plains

0 80 160 320 Kilometers

N

State Boundaries
Highways
Major Rivers
Waterbodies
Model Region
Model Subregion
Non-habitat Area

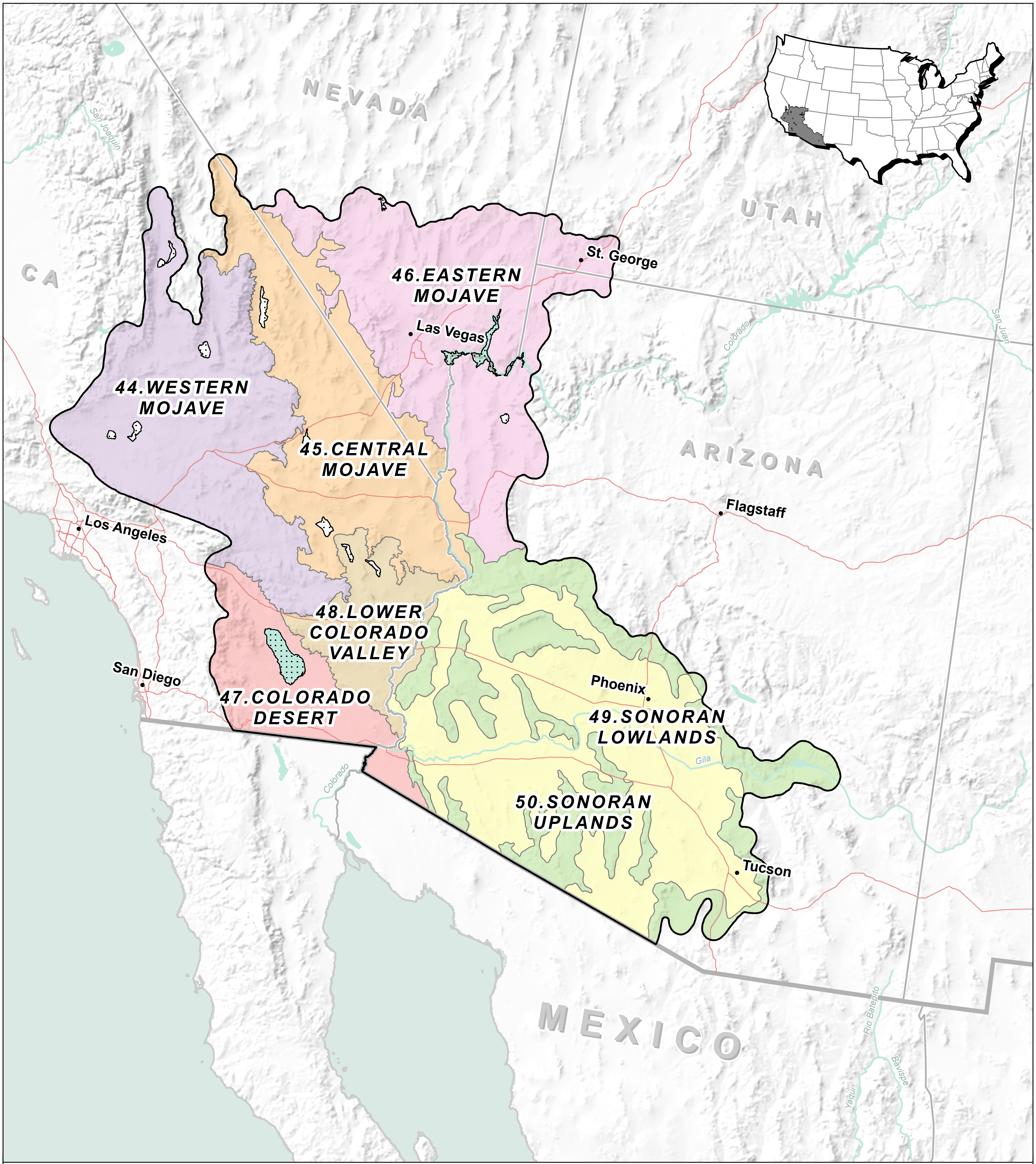

Golden Eagle Nest Site Model

Model Subregions

Southwestern Deserts & Madrean Archipelago

0 65 130 260 Kilometers

N

State Boundaries
Highways
Major Rivers
Waterbodies

Model Region
Model Subregion
Non-habitat Area

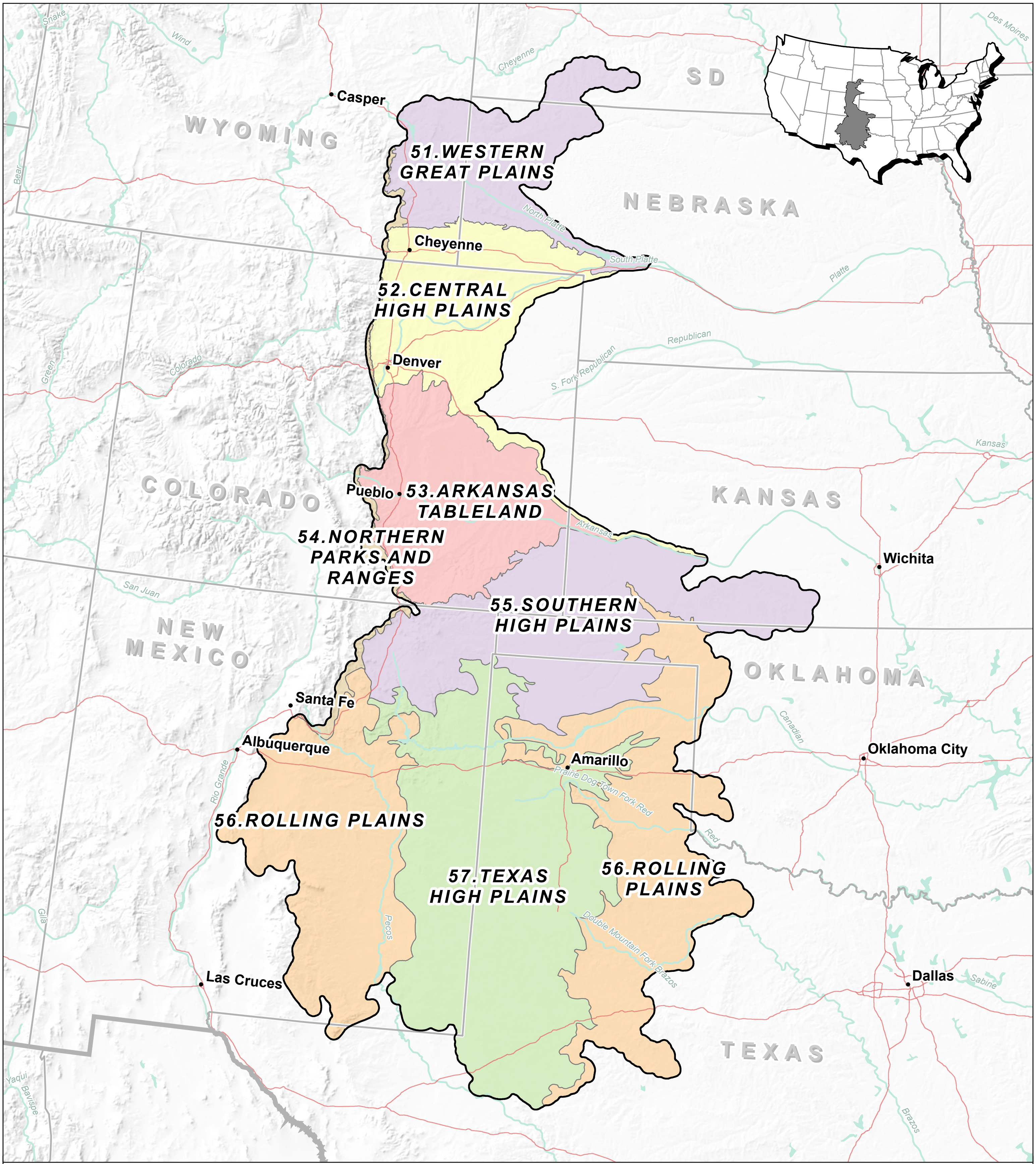

Golden Eagle Nest Site Model

Model Subregions

Southwestern Plains

0 95 190 380 Kilometers

N

State Boundaries

Highways

Major Rivers

Waterbodies

Model Region

Model Subregion

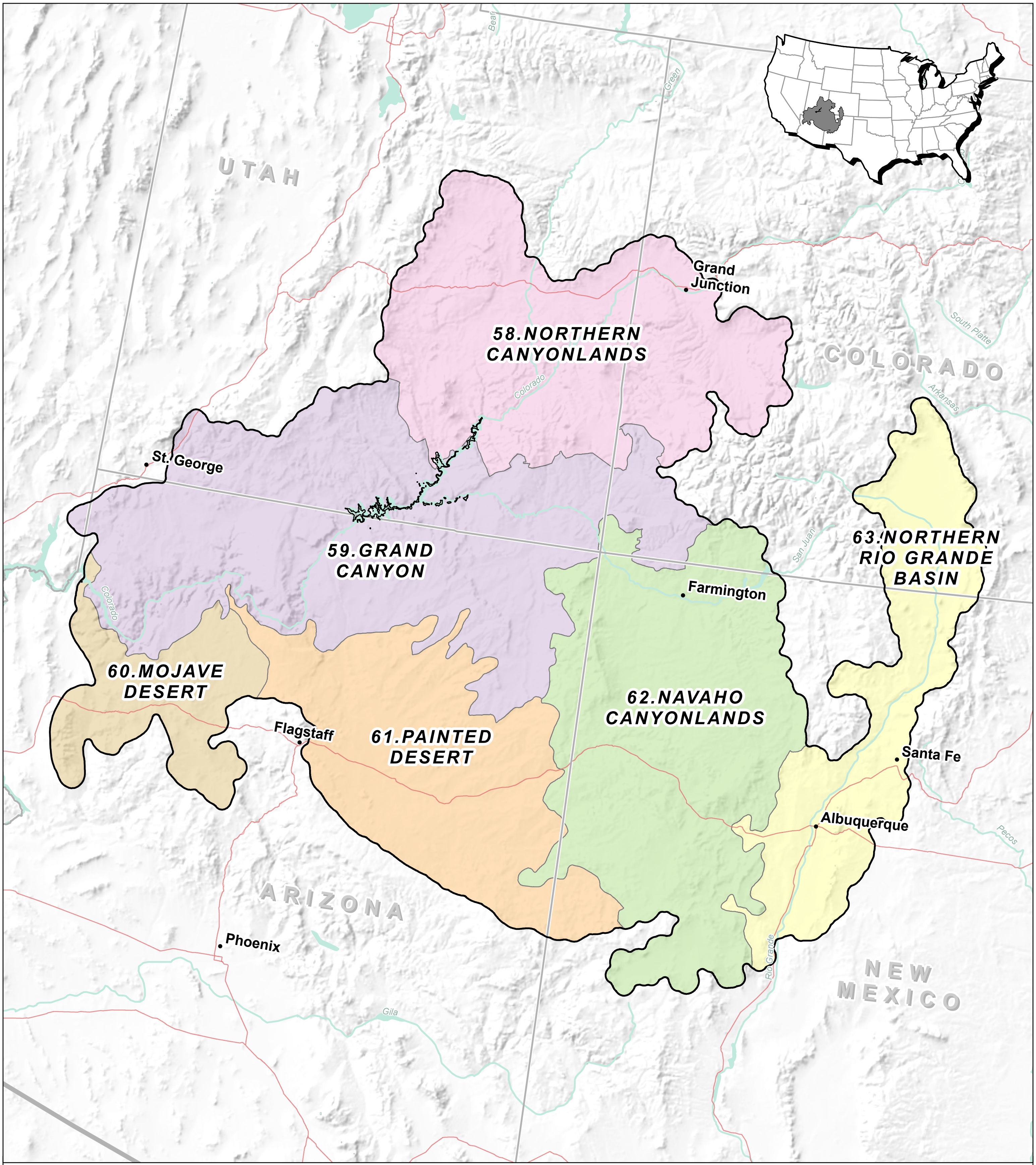

Golden Eagle Nest Site Model

Model Subregions

Southwestern Plateaus

0 62.5 125 250 Kilometers

N

State Boundaries

Highways

Major Rivers

Waterbodies

Model Region

Model Subregion

Non-habitat Area

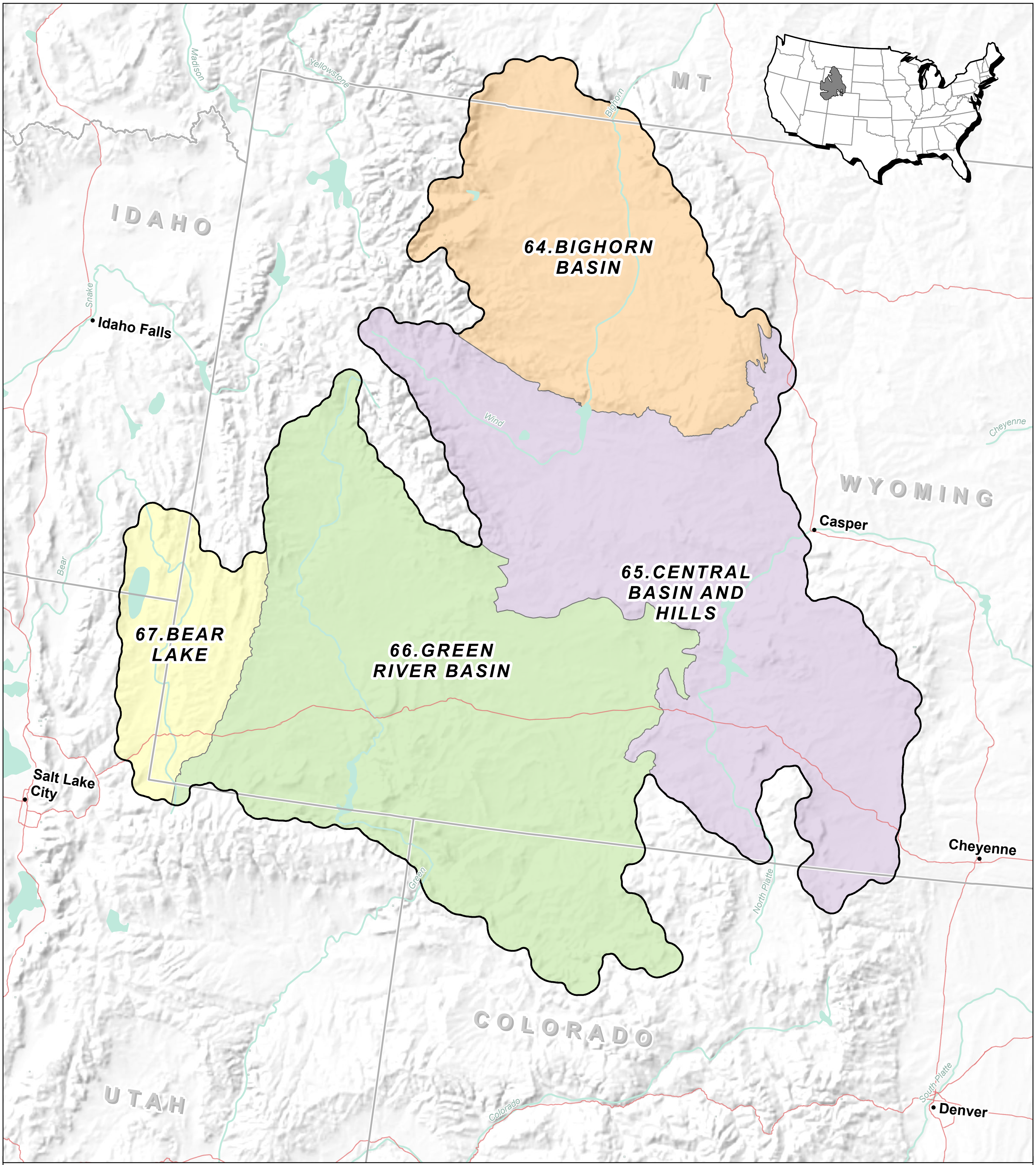

Golden Eagle Nest Site Model

Model Subregions

Wyoming Basin & Uinta Basin and North Park

0 40 80 160 Kilometers

N

State Boundaries
Highways
Major Rivers
Waterbodies
Model Region
Model Subregion
