# Supplementary material for: Modeling spatial variation in density of golden eagle nest sites in the western United States
Source: PLoS One. 2019 Sep 30;14(9):e0223143. doi: 10.1371/journal.pone.0223143 (PMC6768475; doi:10.1371/journal.pone.0223143)

S3 Fig. Functional forms and percent contribution of model covariates for twelve modeling regions of the western United States.

(A) California Foothills

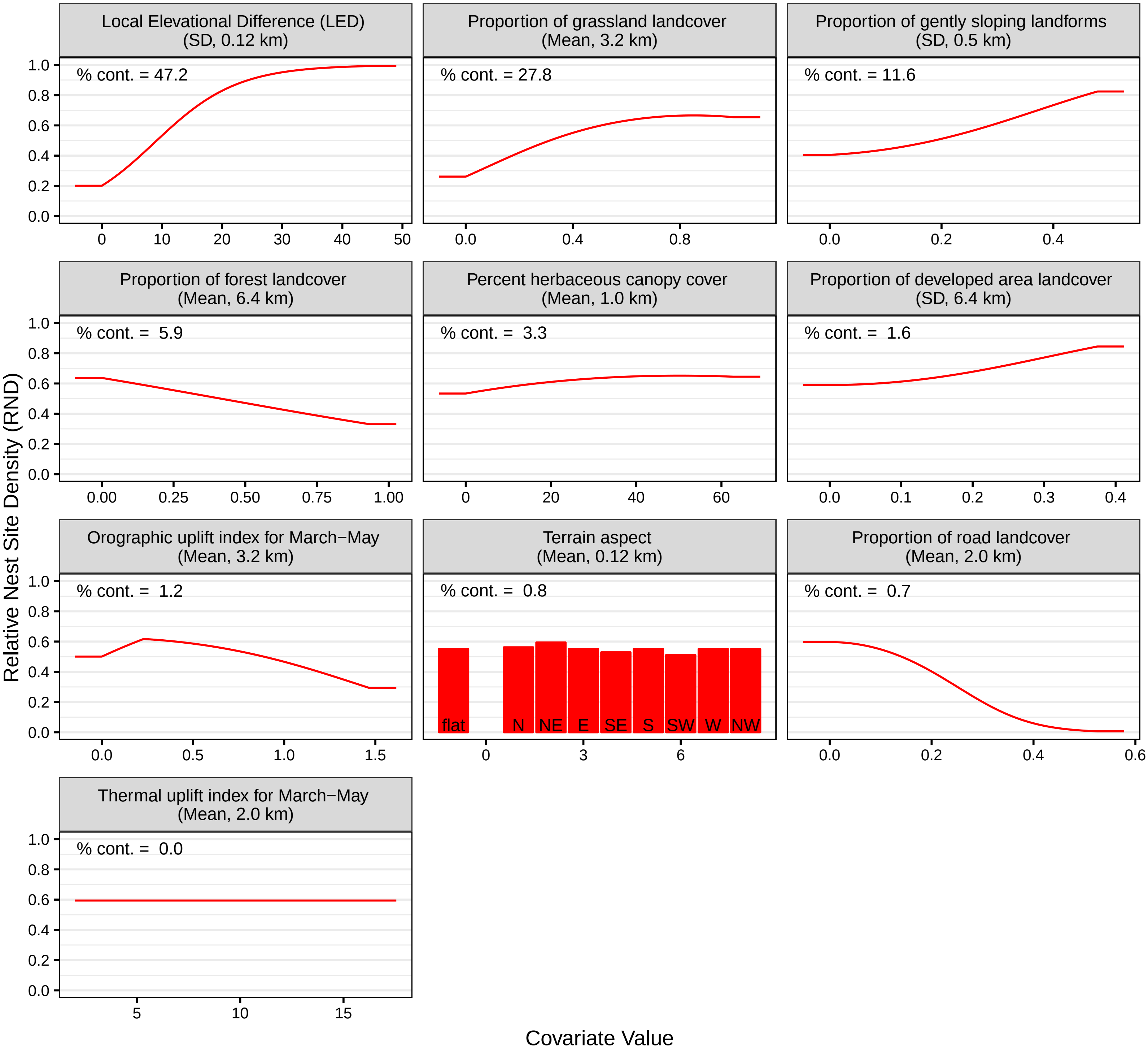

S3 Fig. Functional forms and percent contribution of model covariates for twelve modeling regions of the western United States.

(B) Central Basin and Range

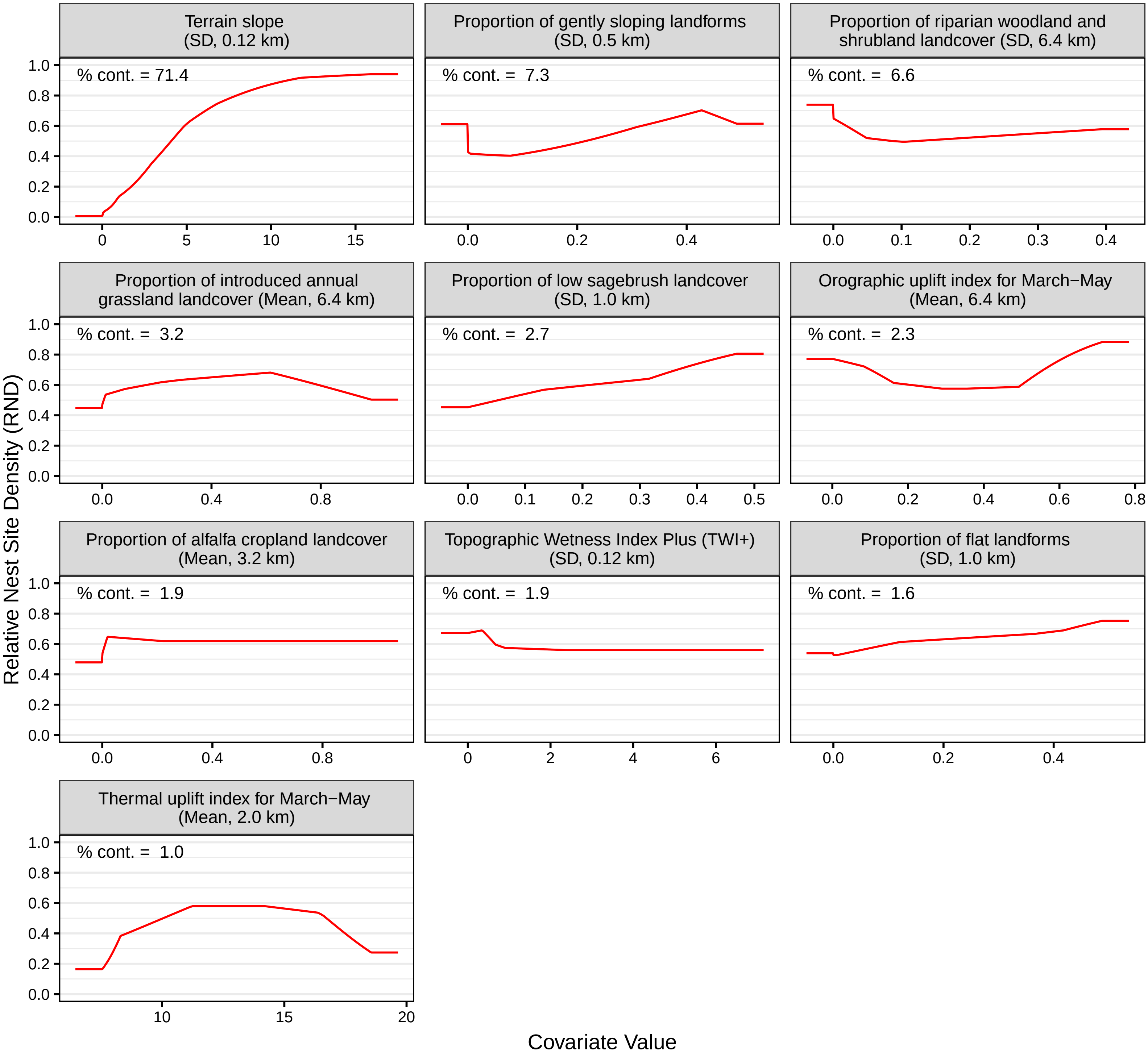

S3 Fig. Functional forms and percent contribution of model covariates for twelve modeling regions of the western United States.

(C) Chihuahuan Desert

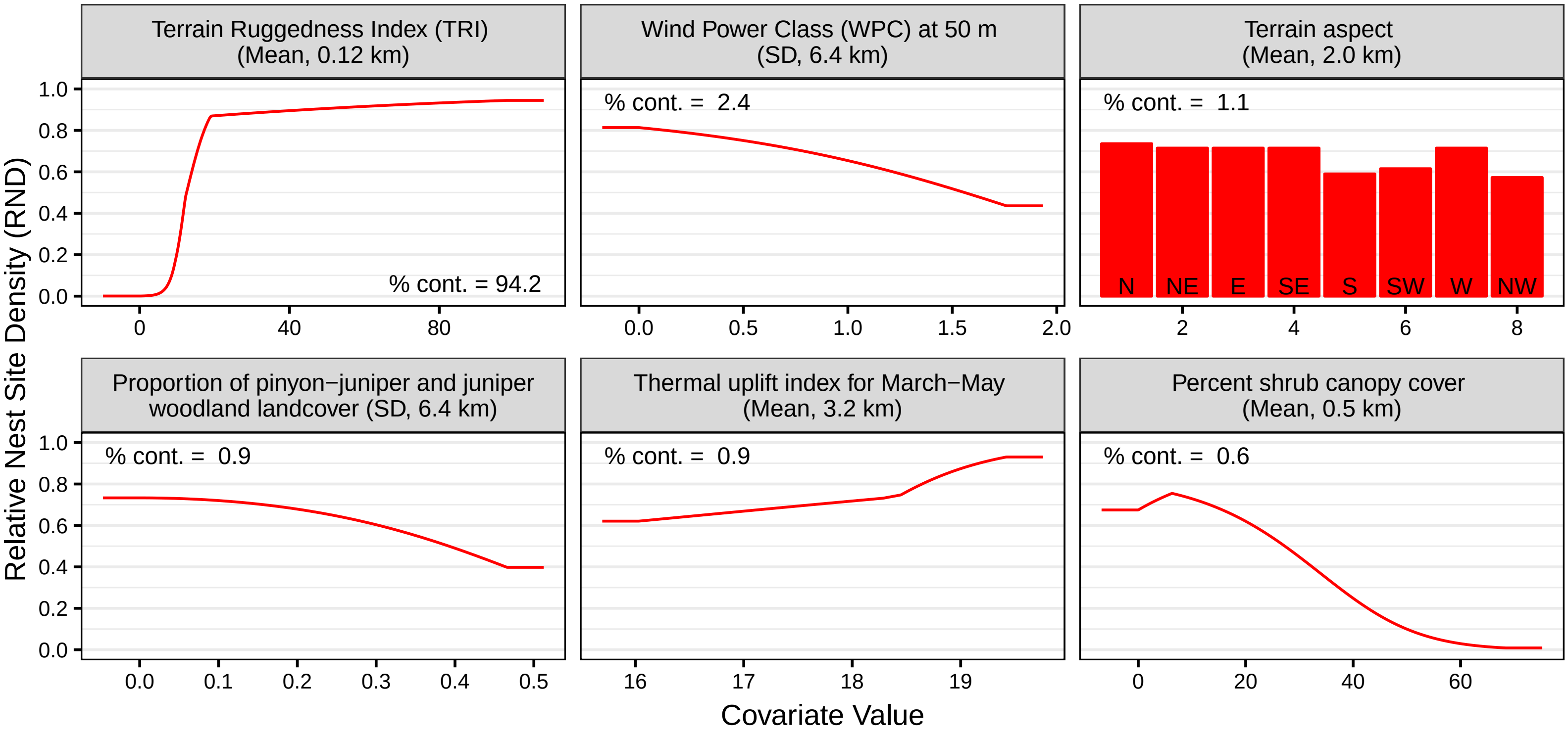

S3 Fig. Functional forms and percent contribution of model covariates for twelve modeling regions of the western United States.

(D) Columbia Plateau

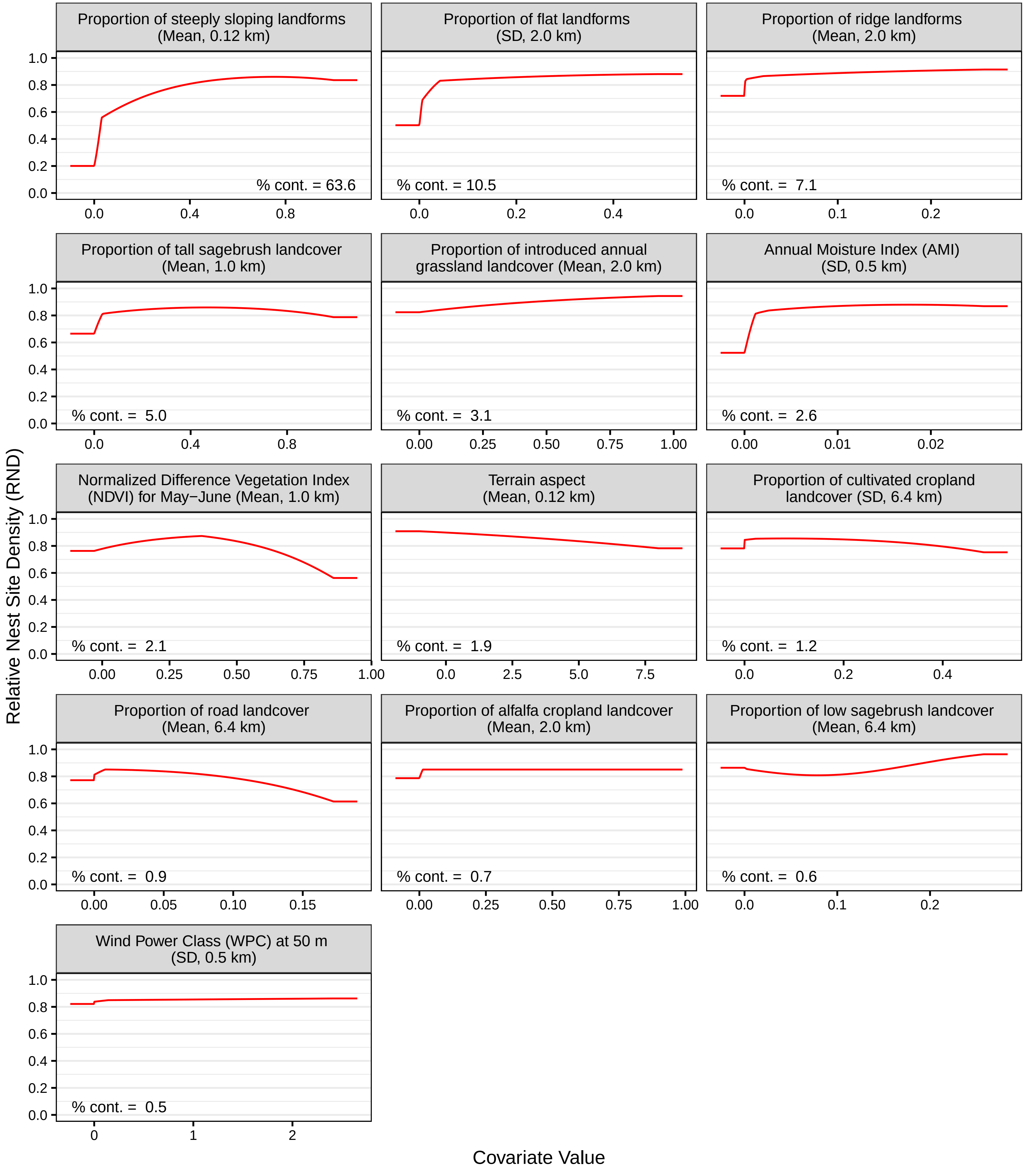

S3 Fig. Functional forms and percent contribution of model covariates for twelve modeling regions of the western United States.

(E) Forested Montane

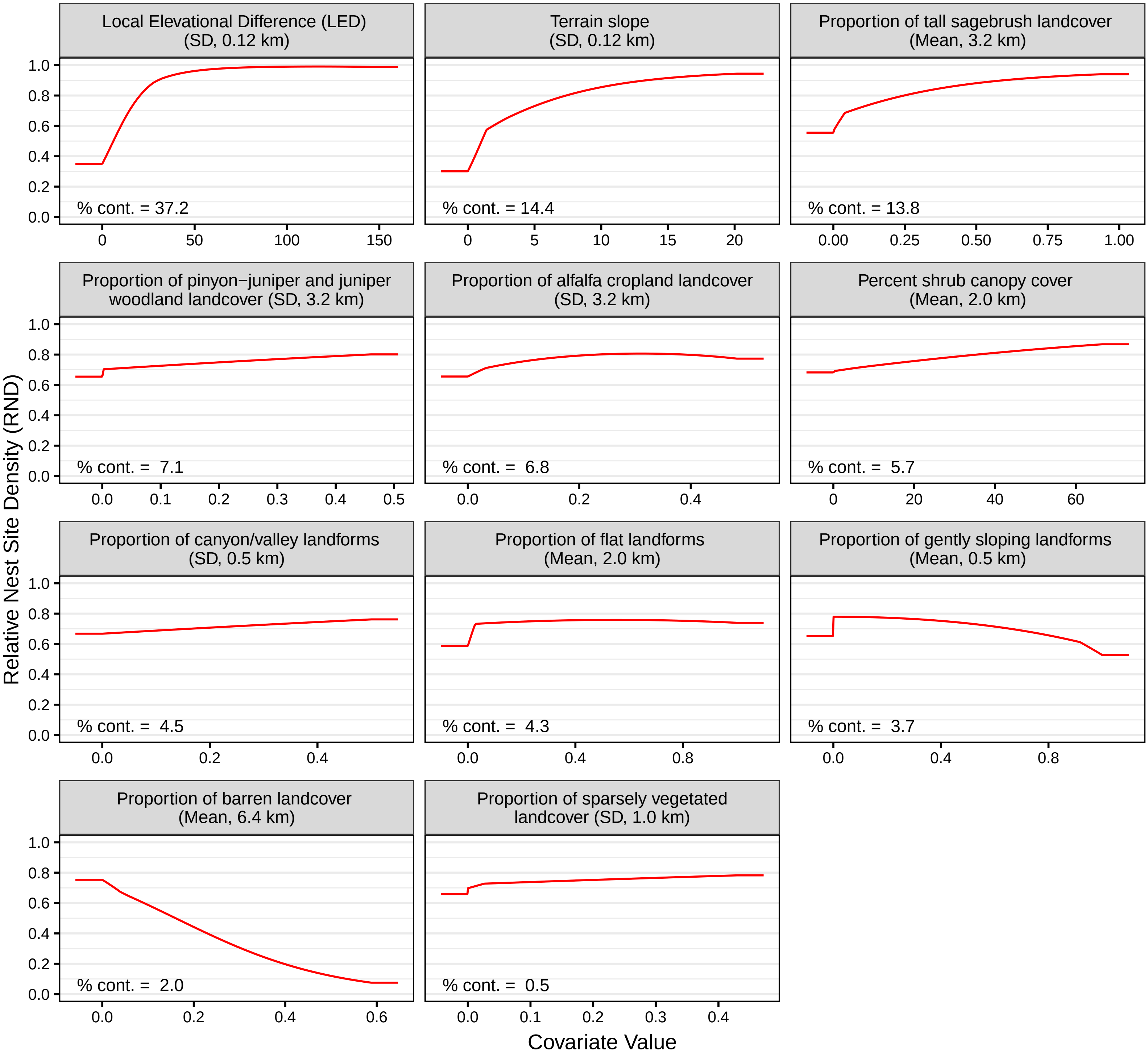

S3 Fig. Functional forms and percent contribution of model covariates for twelve modeling regions of the western United States.

(F) Intermontane Basins and Valleys

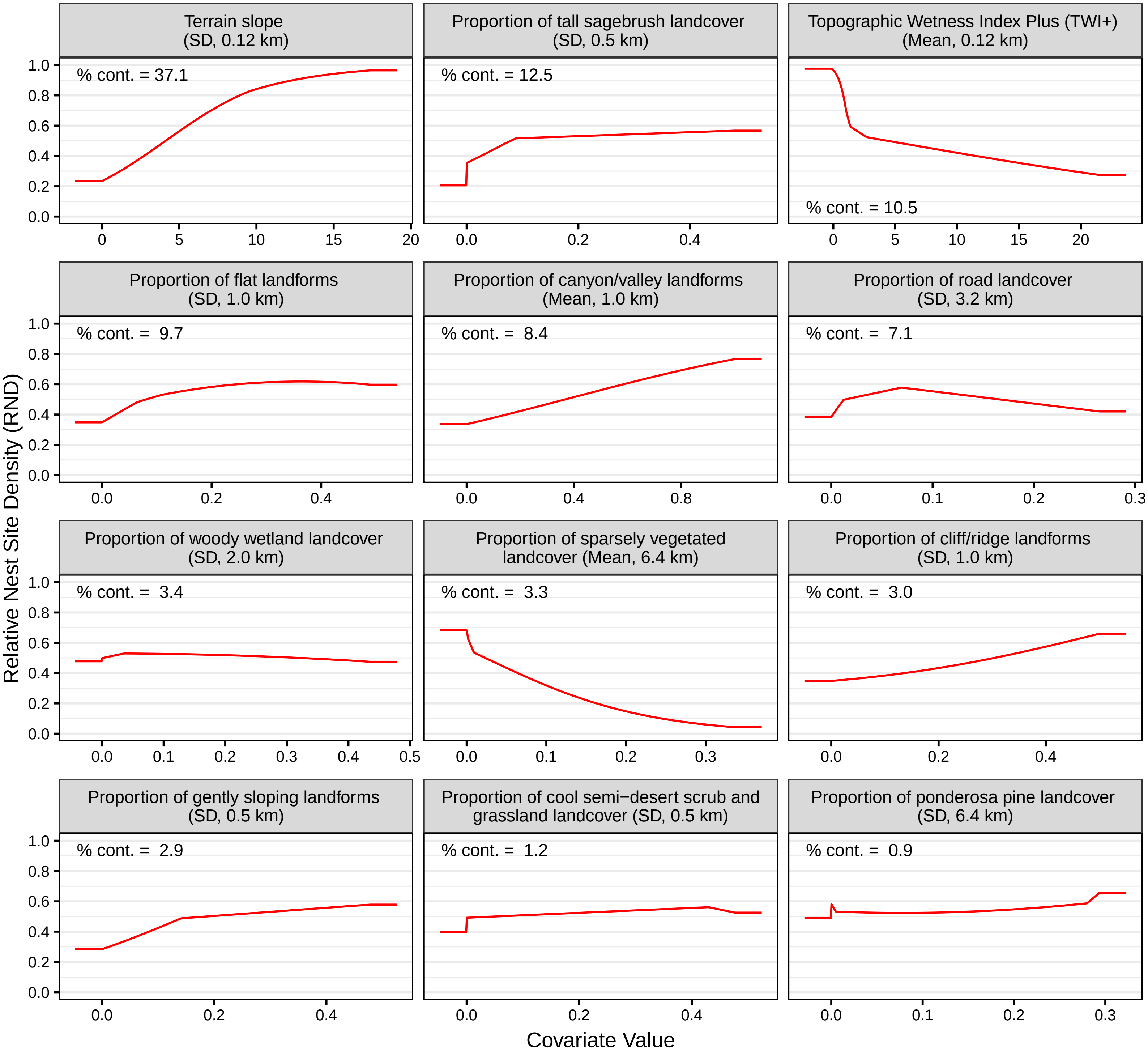

S3 Fig. Functional forms and percent contribution of model covariates for twelve modeling regions of the western United States.

(G) Northern Great Basin

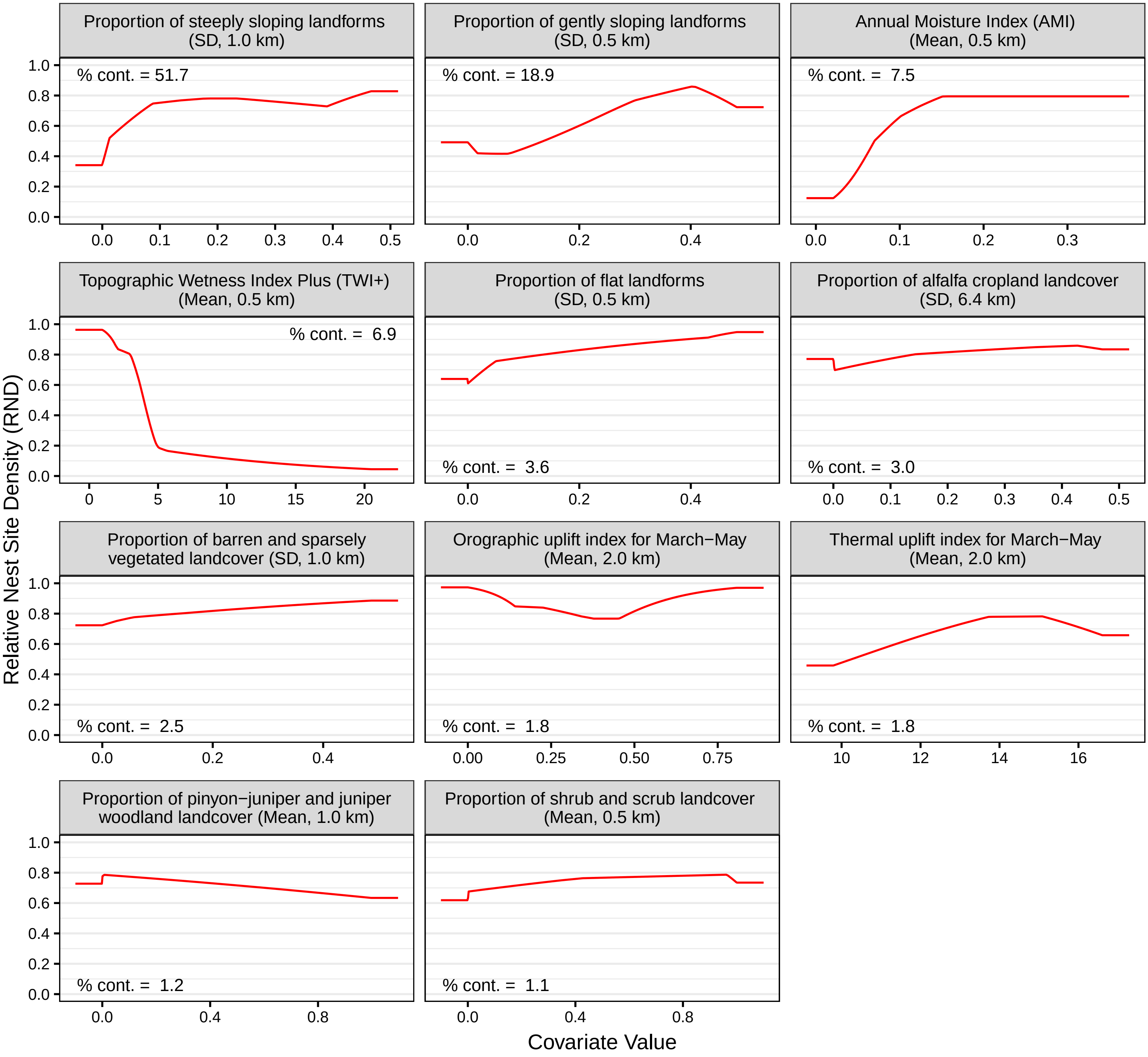

S3 Fig. Functional forms and percent contribution of model covariates for twelve modeling regions of the western United States.

(H) Northwestern Plains

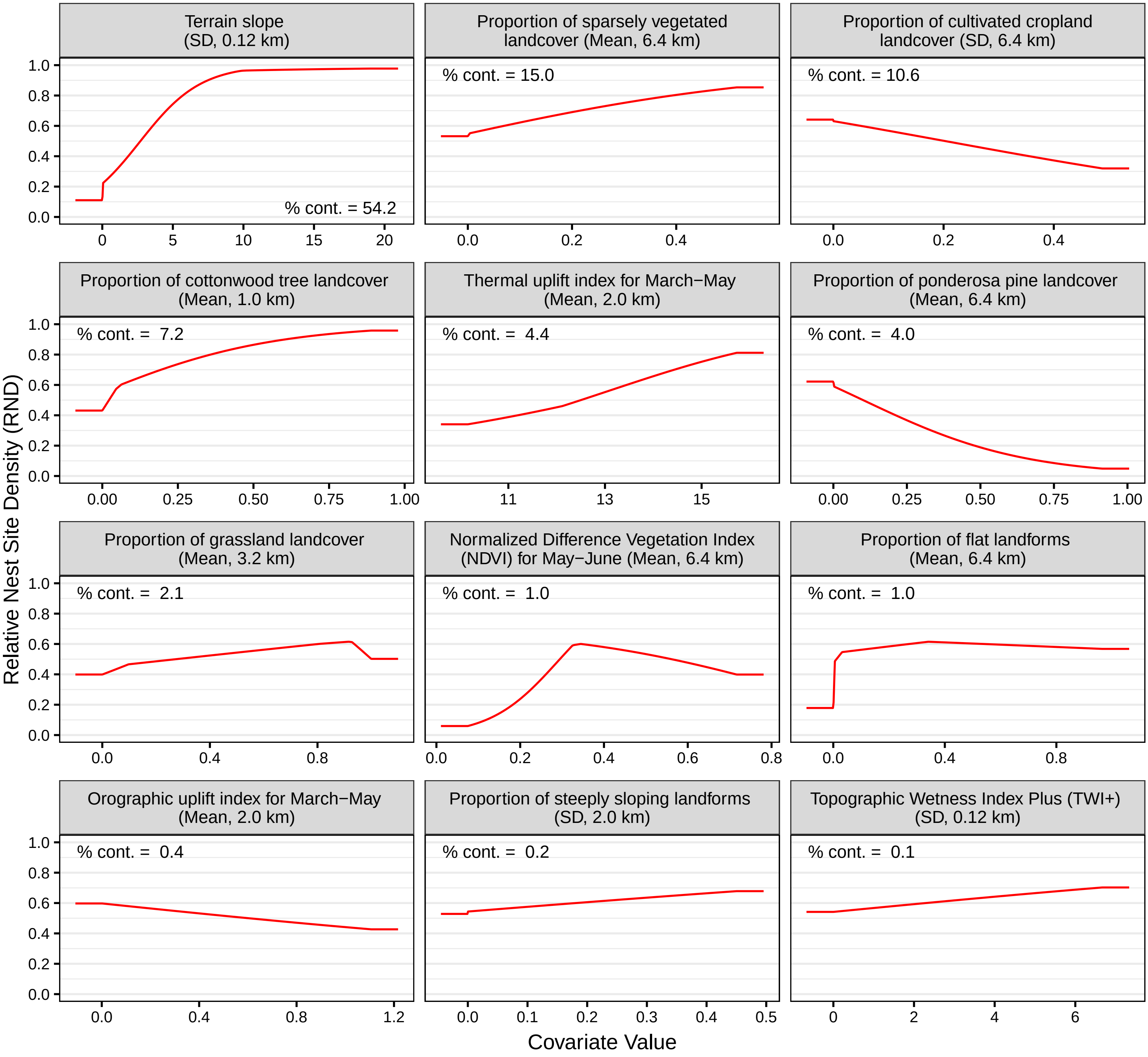

S3 Fig. Functional forms and percent contribution of model covariates for twelve modeling regions of the western United States.

(I) Southwestern Deserts

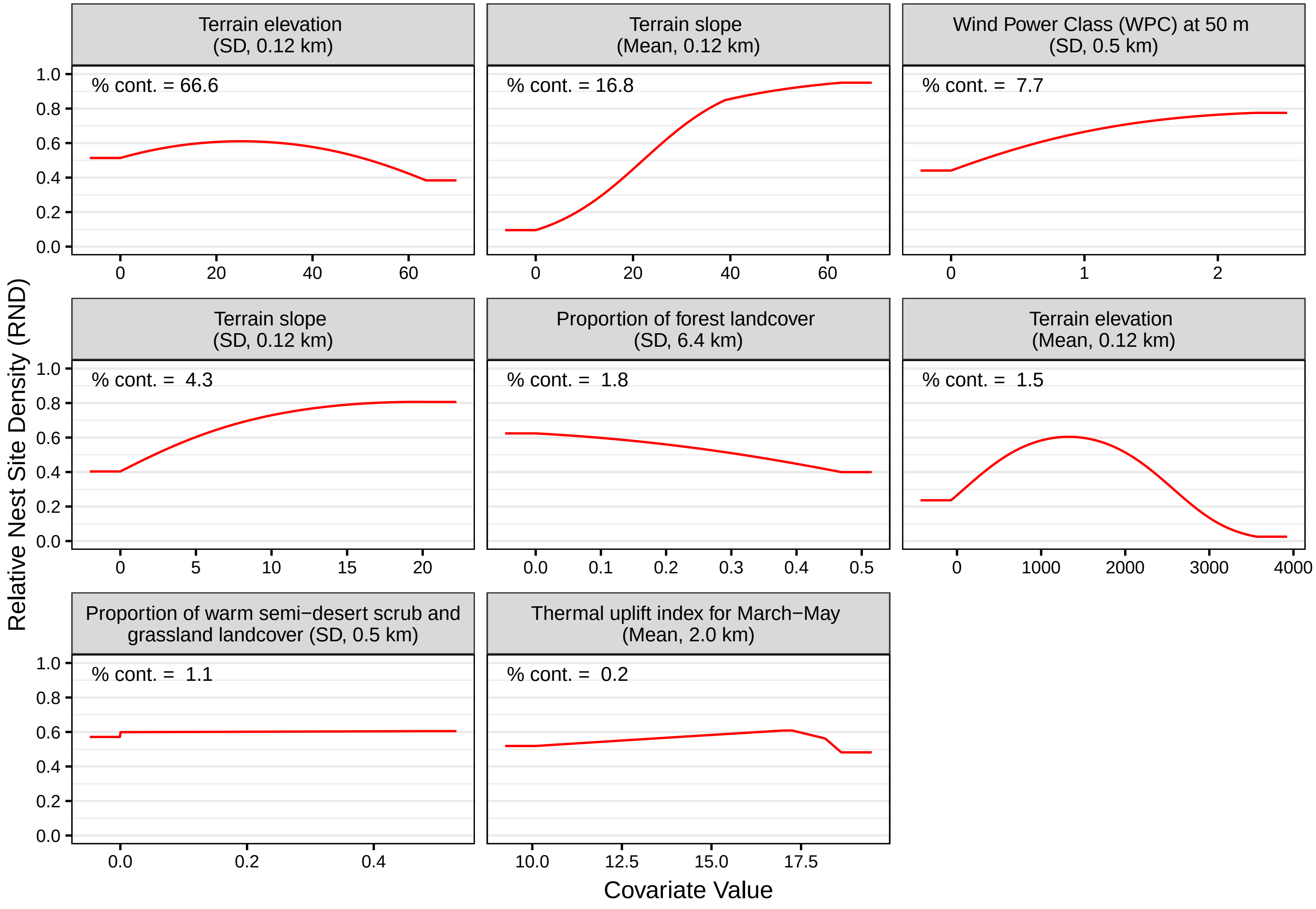

S3 Fig. Functional forms and percent contribution of model covariates for twelve modeling regions of the western United States.

(J) Southwestern Plains

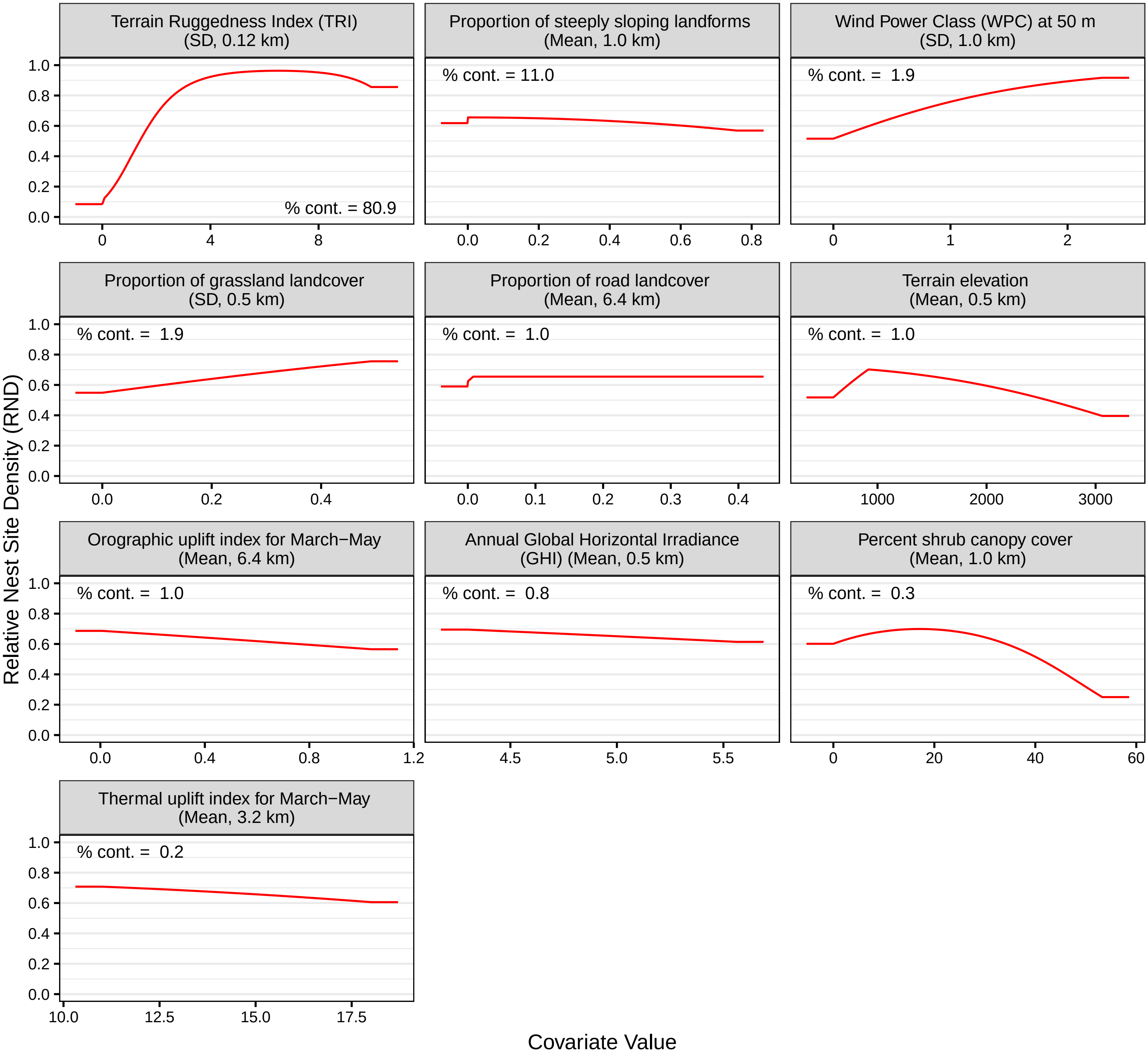

S3 Fig. Functional forms and percent contribution of model covariates for twelve modeling regions of the western United States.

(K) Southwestern Plateaus

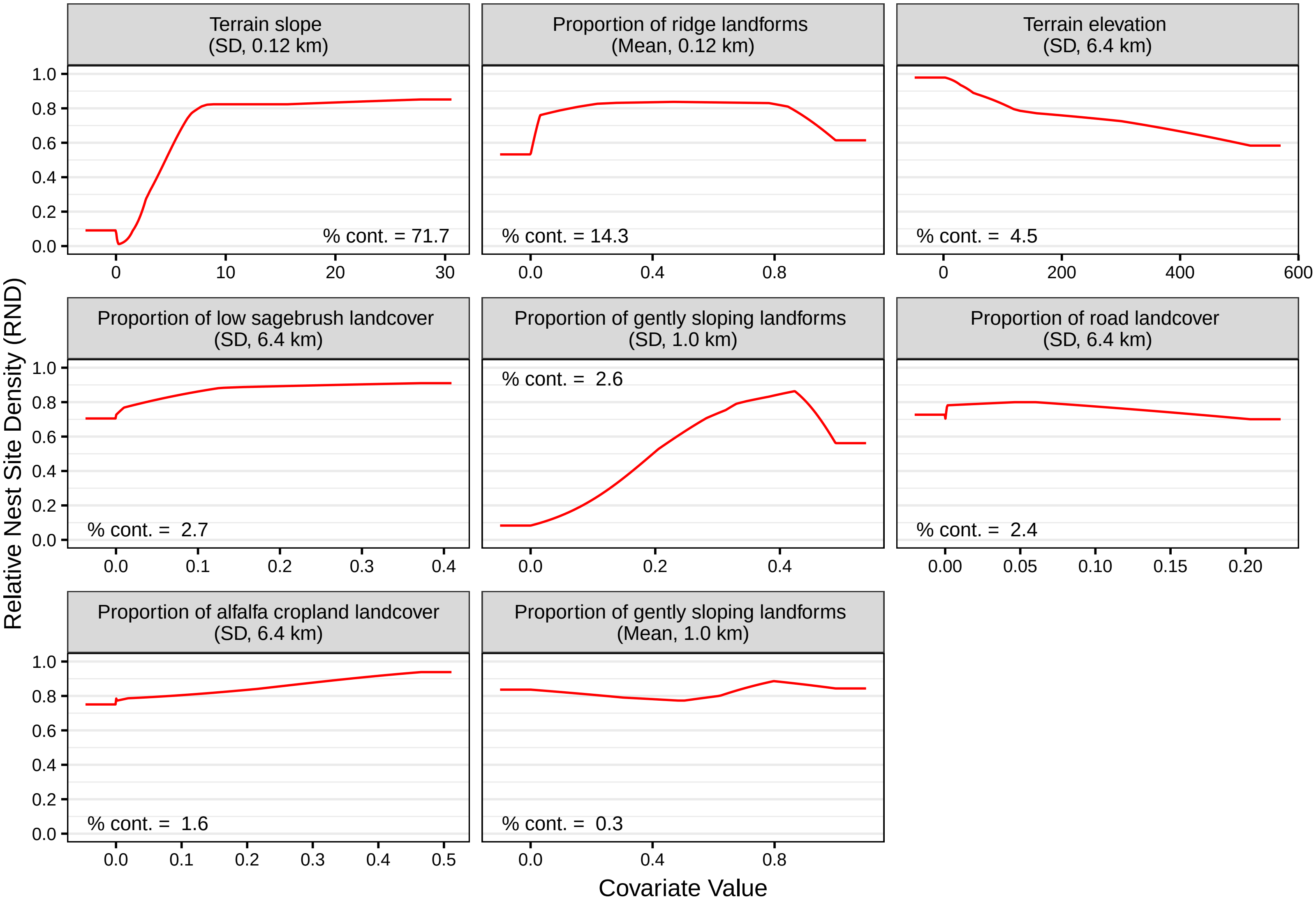

S3 Fig. Functional forms and percent contribution of model covariates for twelve modeling regions of the western United States.

(L) Wyoming Basin

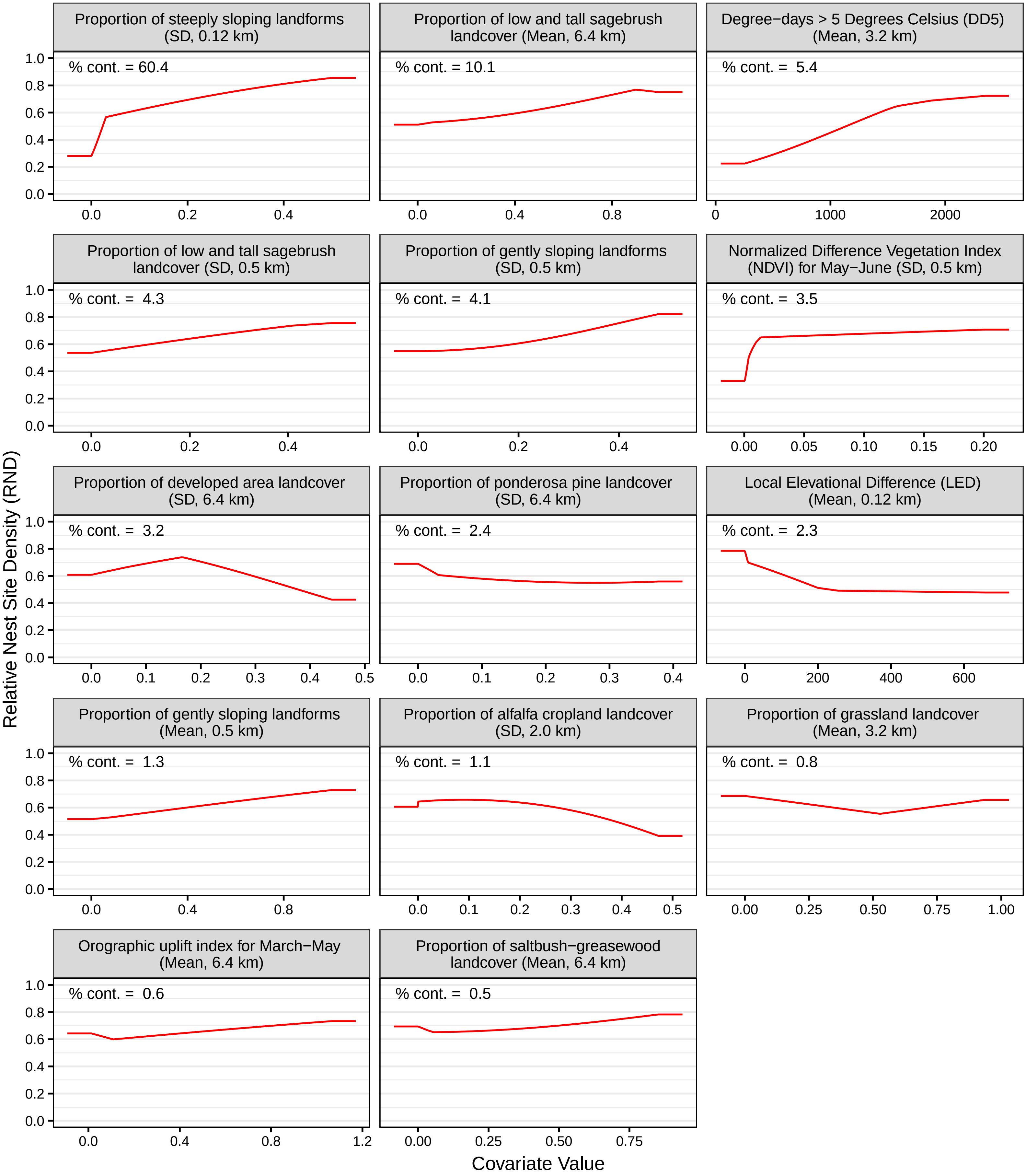

Supplement: S3 Fig — (PDF) [file pone.0223143.s003.pdf]
