# Supplementary material for: Modeling spatial variation in density of golden eagle nest sites in the western United States
Source: PLoS One. 2019 Sep 30;14(9):e0223143. doi: 10.1371/journal.pone.0223143 (PMC6768475; doi:10.1371/journal.pone.0223143)
Supplement: S4 Fig — (PDF) [file pone.0223143.s004.pdf]

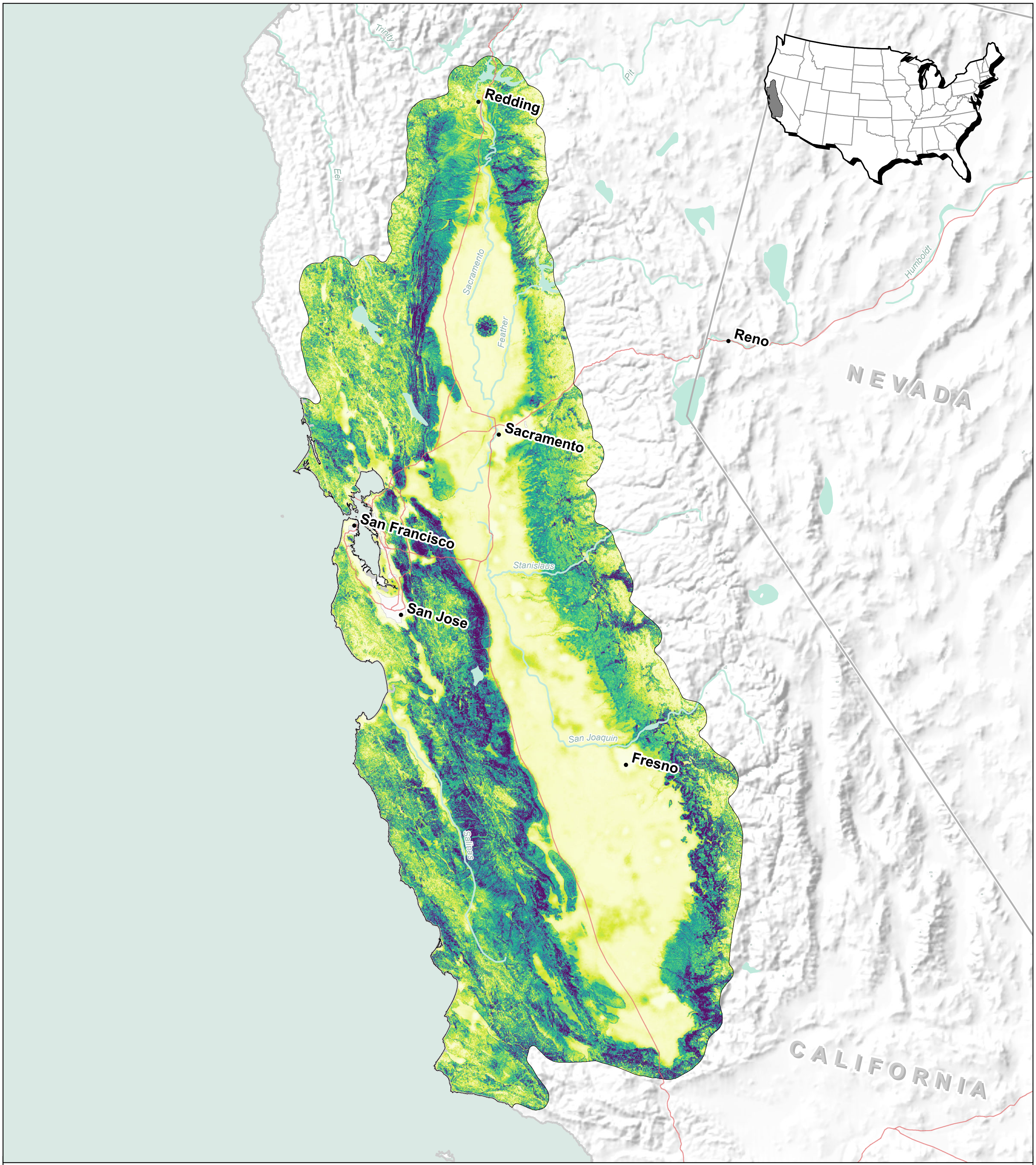

Golden Eagle Nest Site Model

Predicted Relative Nest Site Density (RND)

California Foothills & California Central Valley

0 50 100 200 Kilometers

N

State Boundaries

Highways

Major Rivers

Waterbodies

RND

High

Low

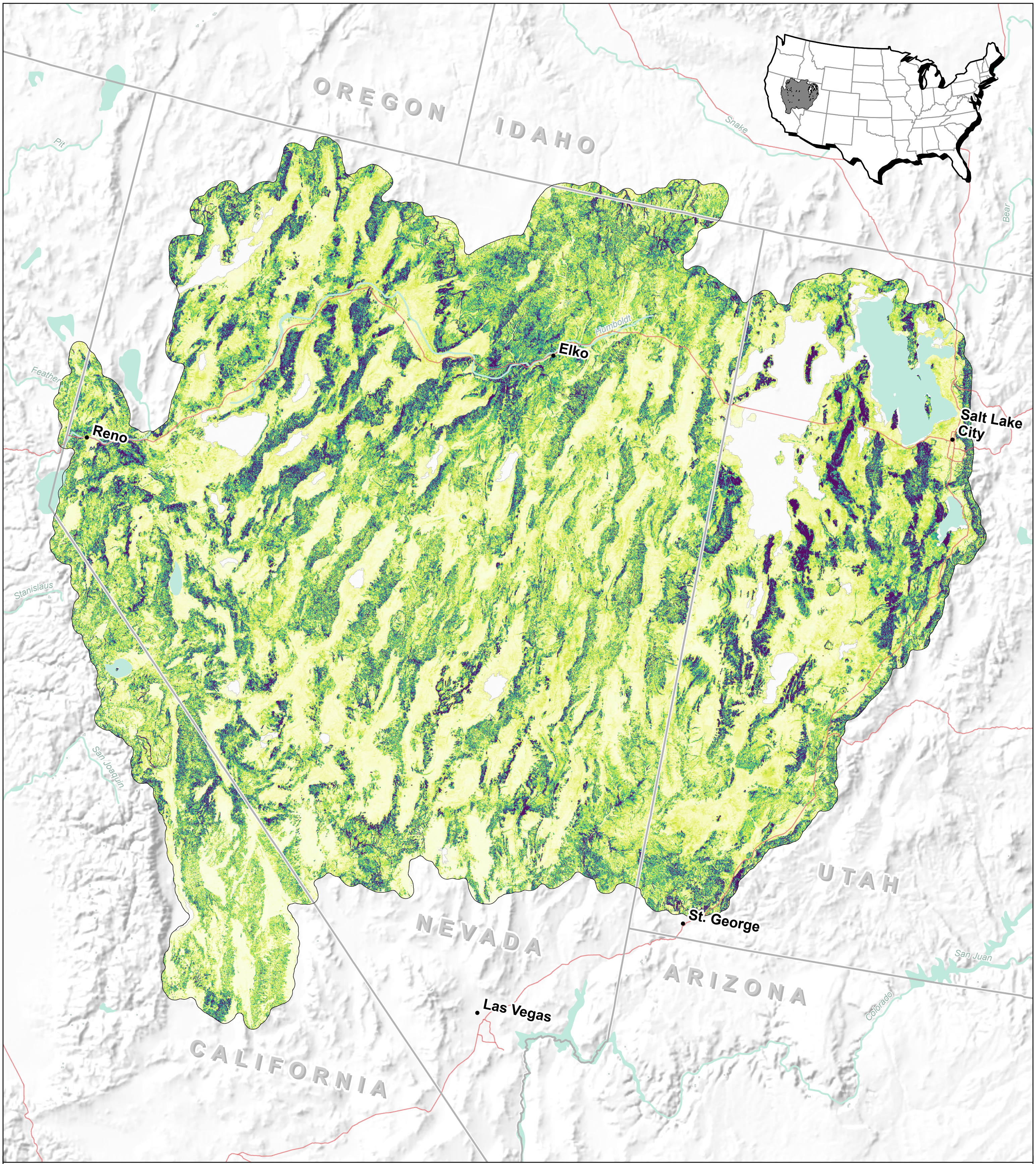

Golden Eagle Nest Site Model

Predicted Relative Nest Site Density (RND)

Central Basin and Range

0 55 110 220 Kilometers

N

State Boundaries
Highways
Major Rivers
Waterbodies

RND
High
Low

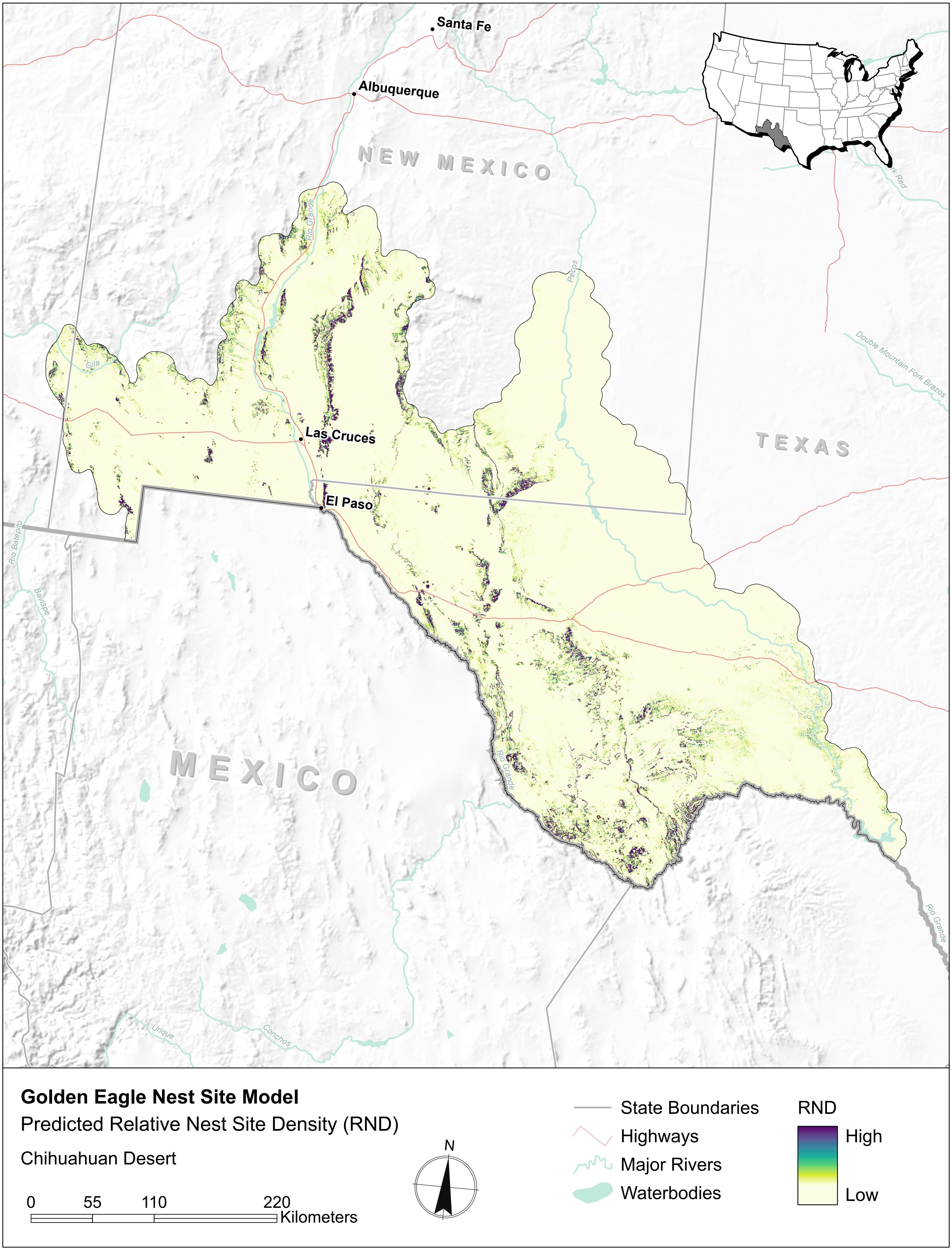
Santa Fe
Albuquerque
NEW MEXICO
TEXAS
MEXICO
Las Cruces
El Paso
Rio Grande
Pecos
Gila
Double Mountain Fork Brazos
Rio Batepito
Bavispe
Urique
Conchos
Rio Grande
Golden Eagle Nest Site Model
Predicted Relative Nest Site Density (RND)
Chihuahuan Desert
0 55 110 220
Kilometers
N
State Boundaries
Highways
Major Rivers
Waterbodies
RND
High
Low

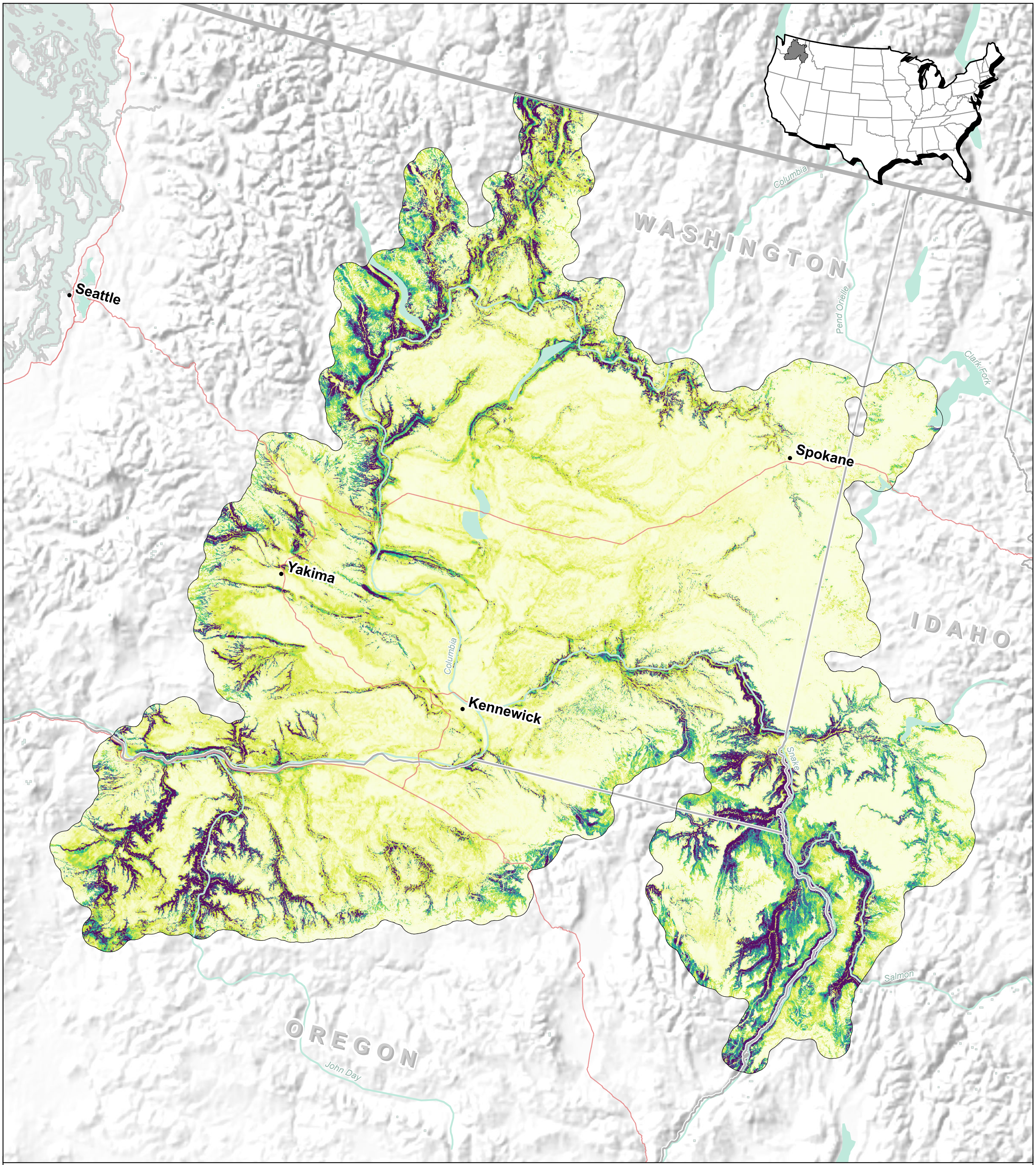

Golden Eagle Nest Site Model

Predicted Relative Nest Site Density (RND)

Columbia Plateau

0 35 70 140 Kilometers

N

State Boundaries

Highways

Major Rivers

Waterbodies

RND

High

Low

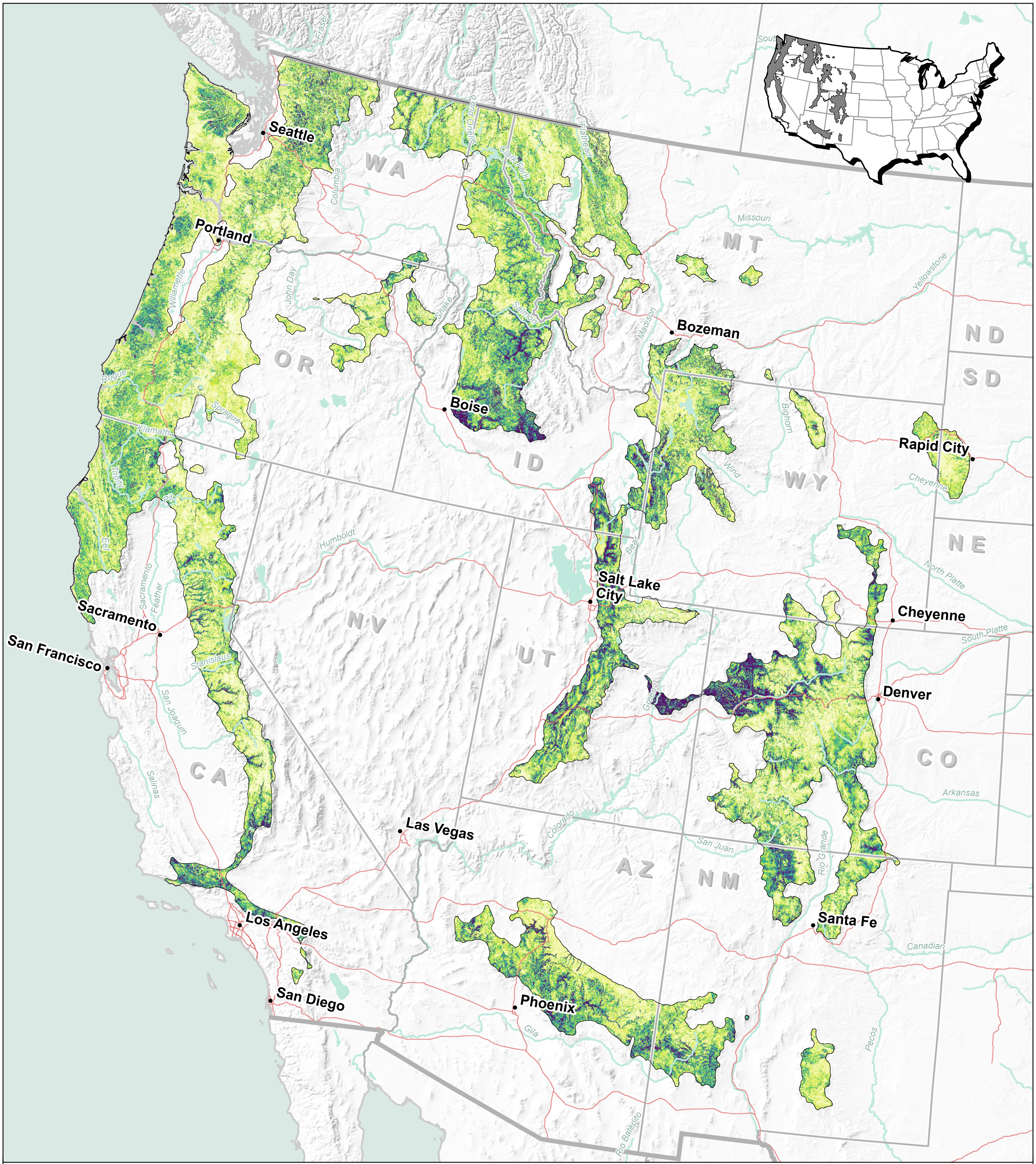

Golden Eagle Nest Site Model

Predicted Relative Nest Site Density (RND)

Forested Montane

0 140 280 560 Kilometers

N

State Boundaries

Highways

Major Rivers

Waterbodies

RND

High

Low

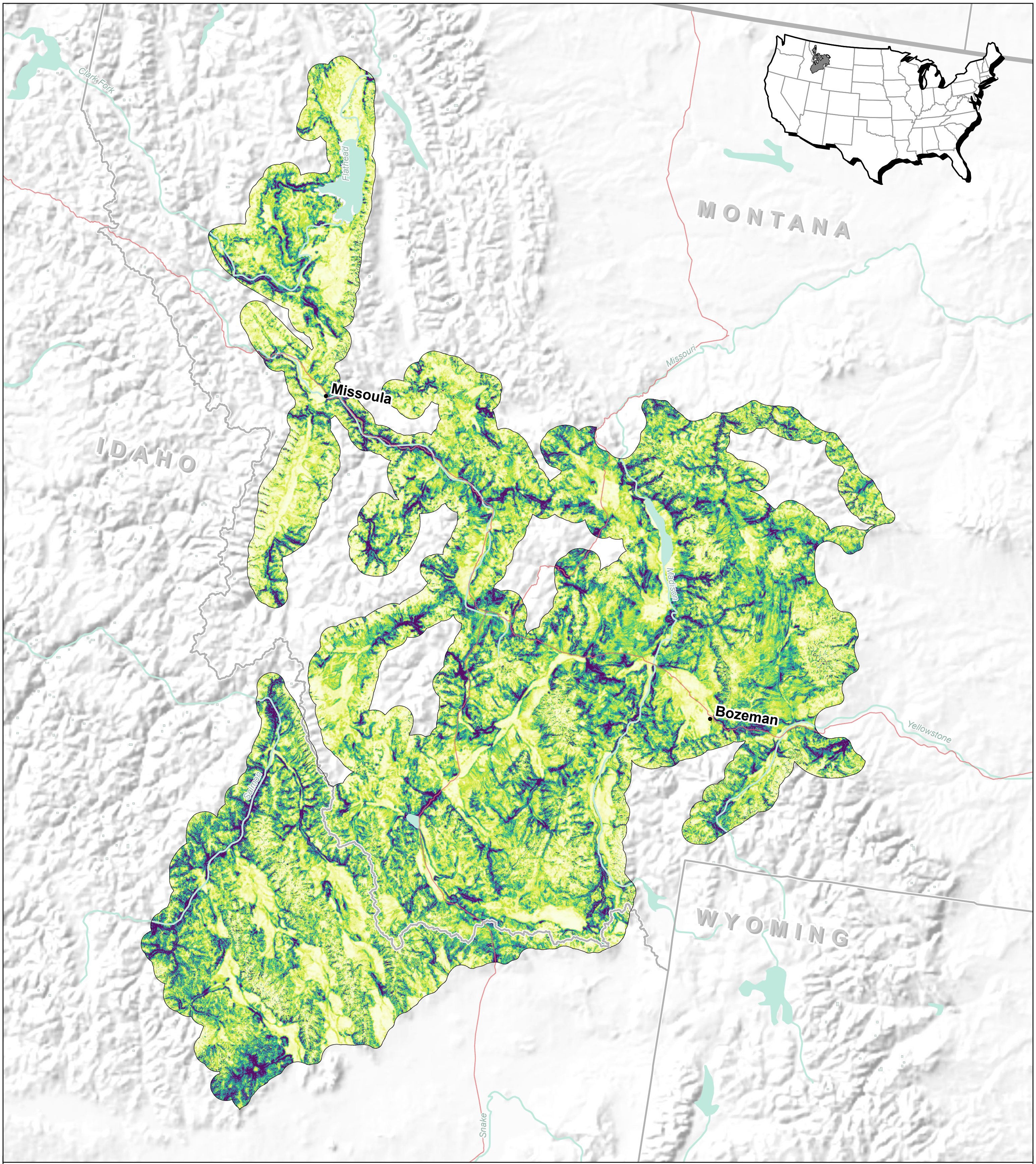

Golden Eagle Nest Site Model

Predicted Relative Nest Site Density (RND)

Intermontane Basins and Valleys

0 37.5 75 150 Kilometers

N

State Boundaries
Highways
Major Rivers
Waterbodies

RND
High
Low

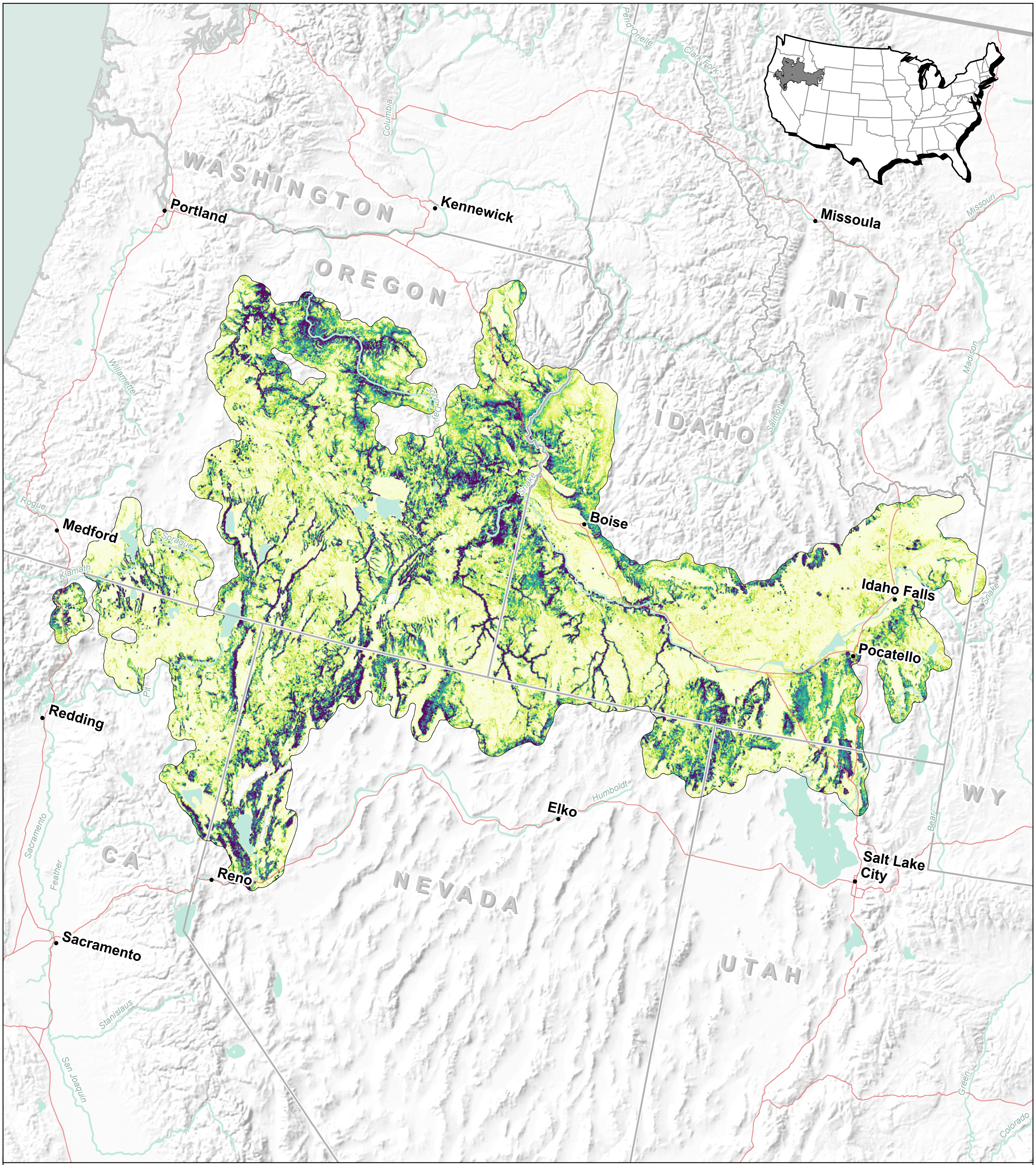

Golden Eagle Nest Site Model

Predicted Relative Nest Site Density (RND)

Northern Great Basin

0 75 150 300 Kilometers

N

State Boundaries

Highways

Major Rivers

Waterbodies

RND

High

Low

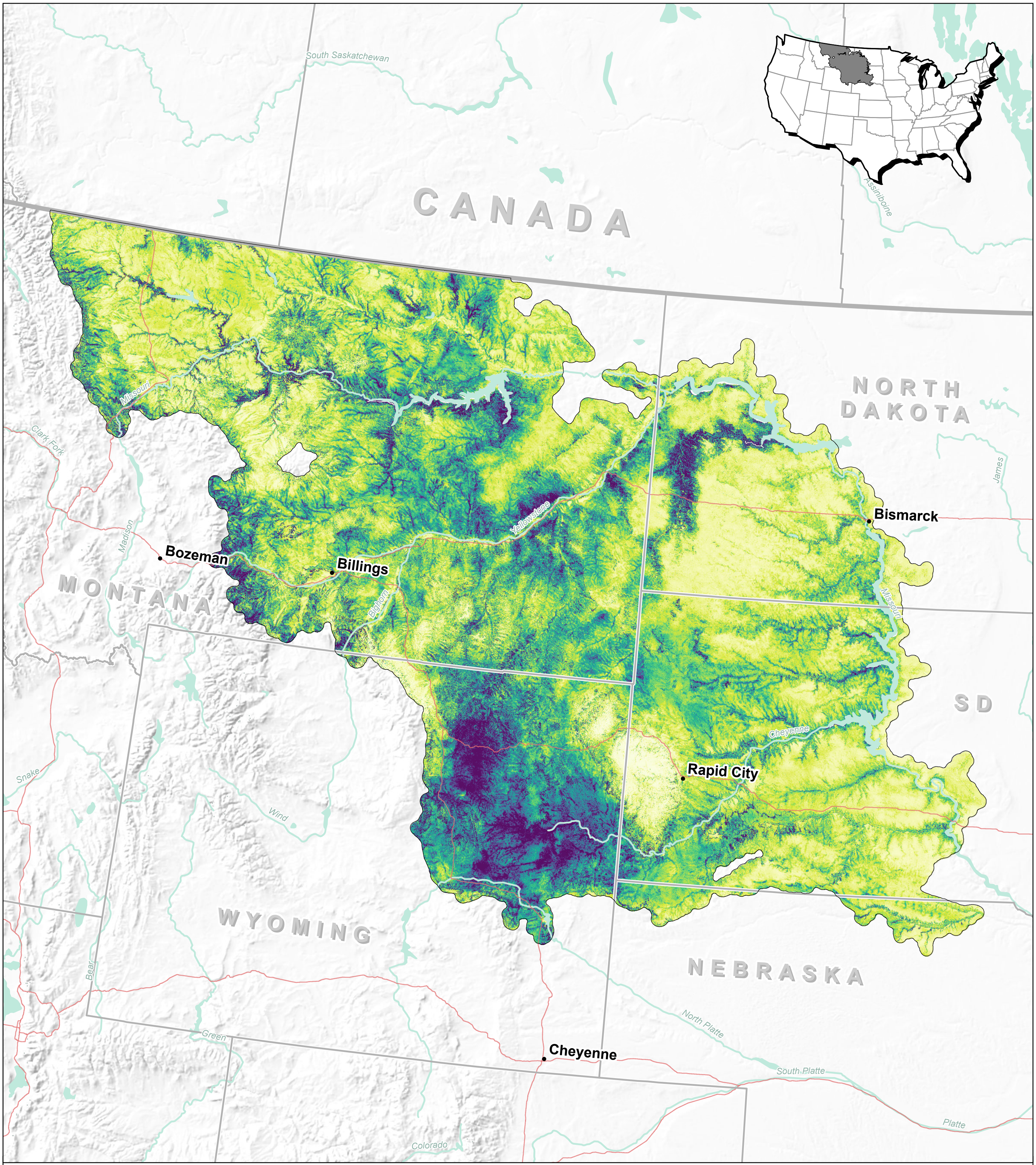

Golden Eagle Nest Site Model

Predicted Relative Nest Site Density (RND)

Northwestern Plains

0 80 160 320 Kilometers

N

State Boundaries
Highways
Major Rivers
Waterbodies

RND
High
Low

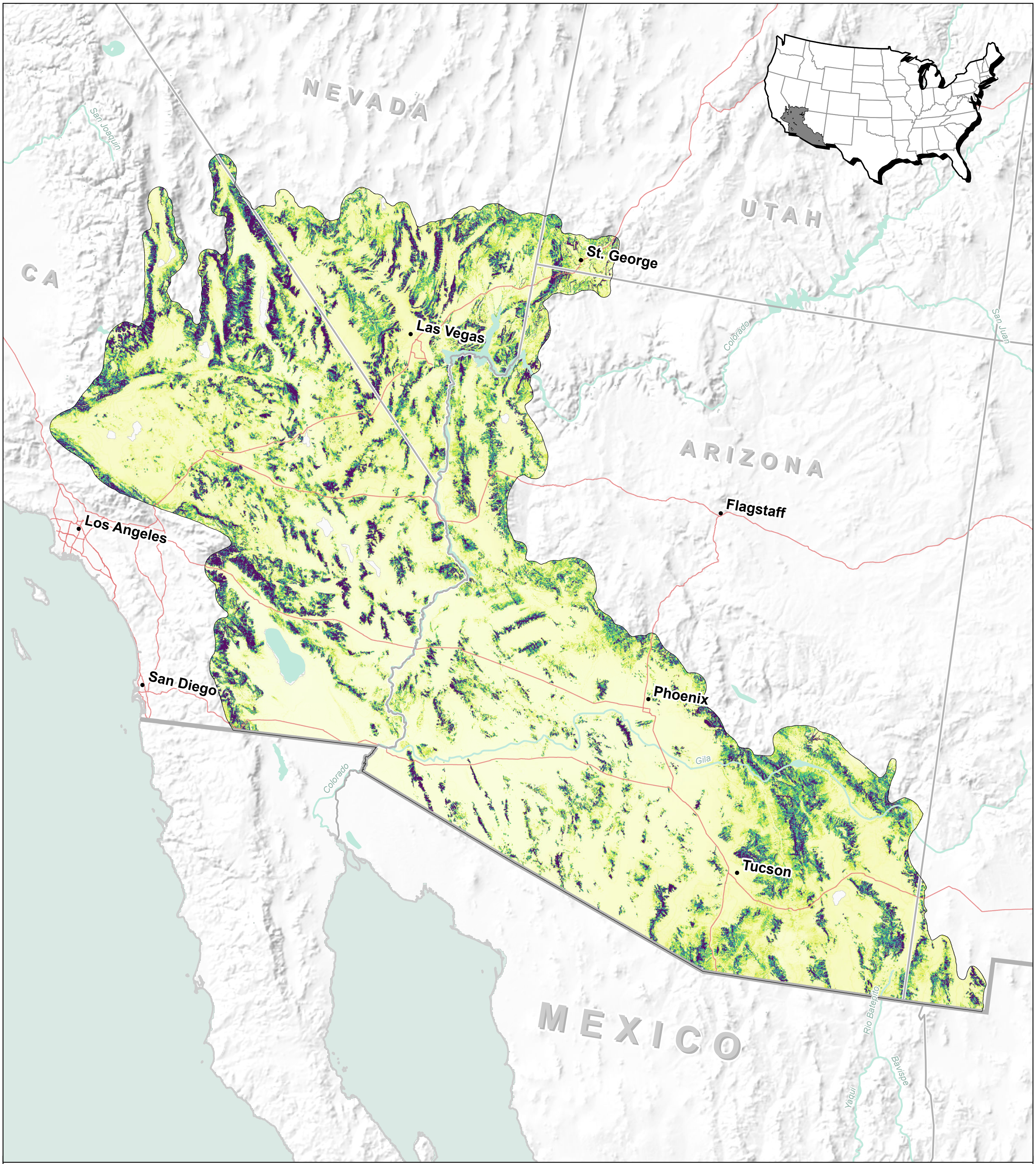

Golden Eagle Nest Site Model

Predicted Relative Nest Site Density (RND)

Southwestern Deserts & Madrean Archipelago

0 65 130 260 Kilometers

N

State Boundaries
Highways
Major Rivers
Waterbodies

RND
High
Low

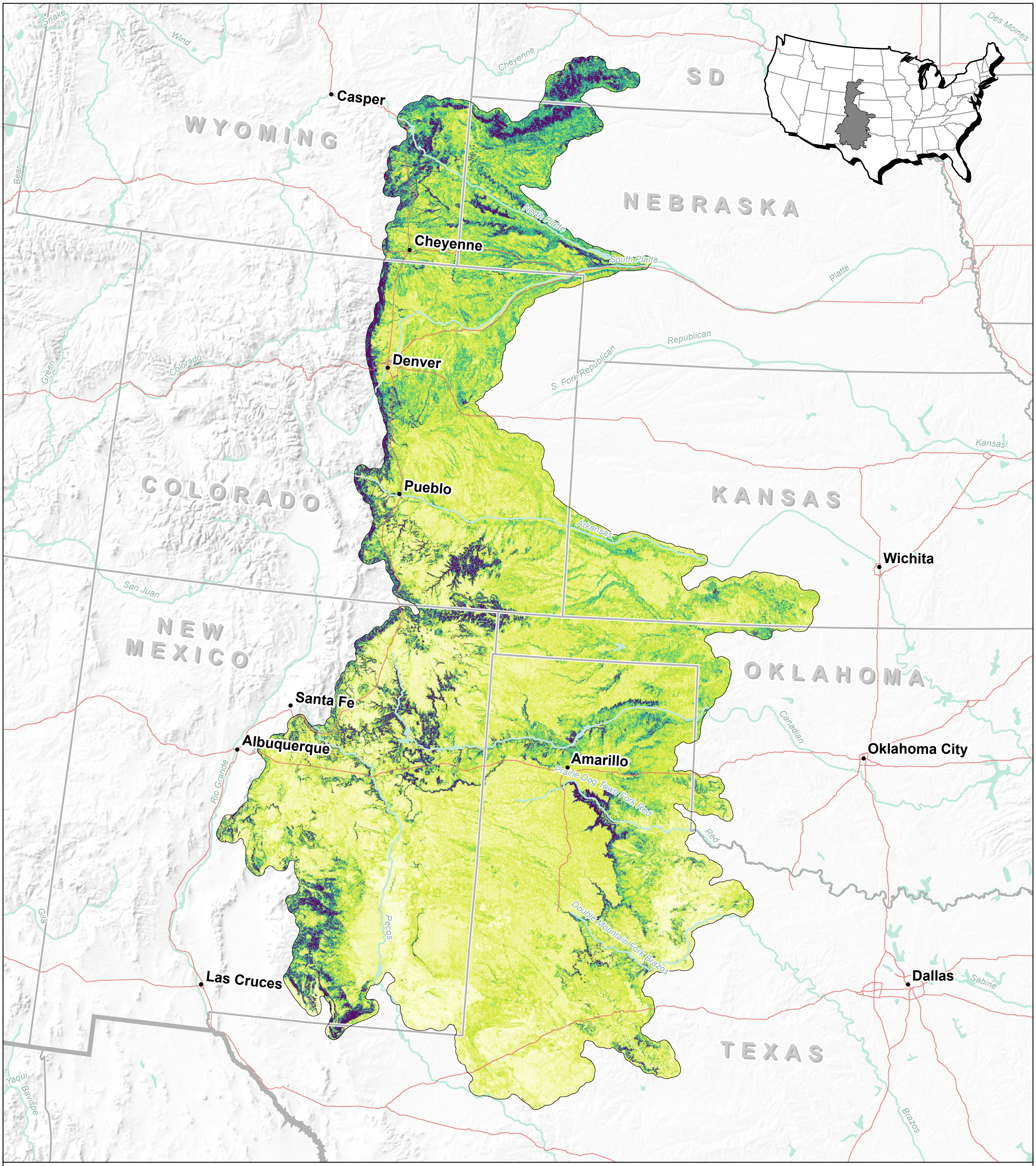

Golden Eagle Nest Site Model

Predicted Relative Nest Site Density (RND)

Southwestern Plains

0 95 190 380 Kilometers

N

State Boundaries
Highways
Major Rivers
Waterbodies

RND
High
Low

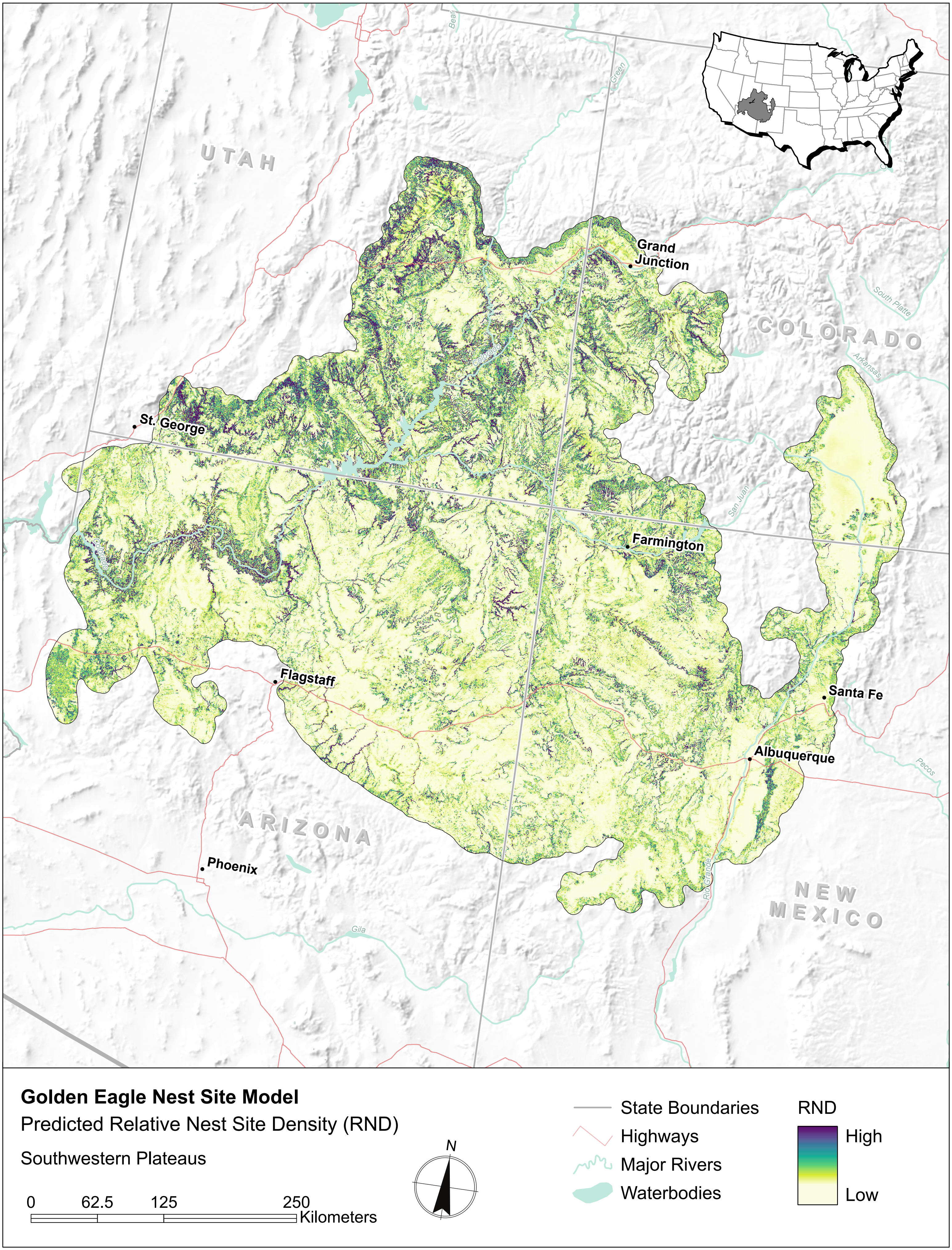

Golden Eagle Nest Site Model

Predicted Relative Nest Site Density (RND)

Southwestern Plateaus

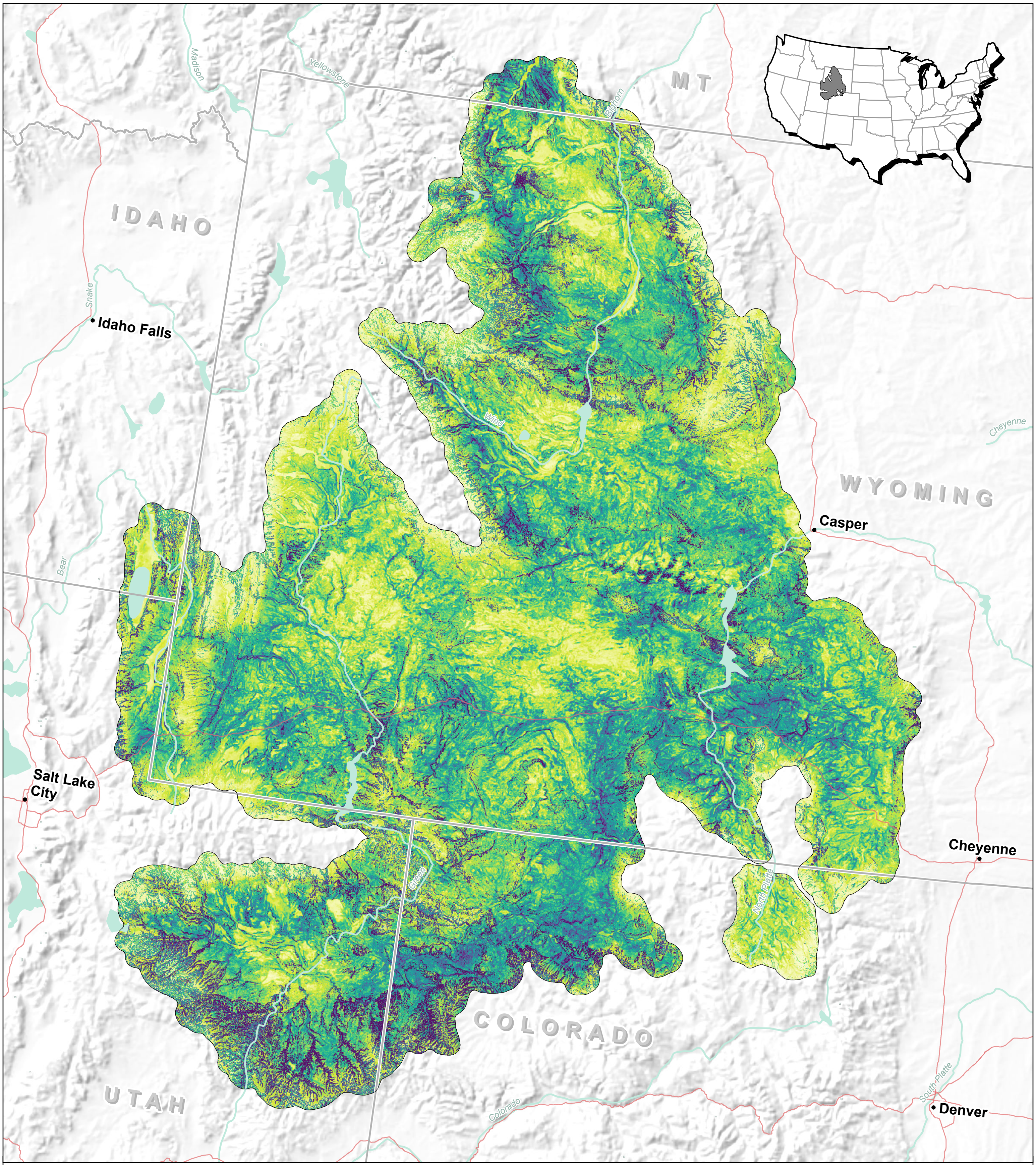

Golden Eagle Nest Site Model

Predicted Relative Nest Site Density (RND)

Wyoming Basin & Uinta Basin and North Park

0 40 80 160 Kilometers

N

State Boundaries
Highways
Major Rivers
Waterbodies

RND
High
Low
