# Supplementary material for: Modeling spatial variation in density of golden eagle nest sites in the western United States
Source: PLoS One. 2019 Sep 30;14(9):e0223143. doi: 10.1371/journal.pone.0223143 (PMC6768475; doi:10.1371/journal.pone.0223143)
Supplement: S2 Table — Percentage of each (A) modeling area and (B) modeling region composed of each of 10 RND classes/bins based on each modeling regions’ final model. (PDF) [file pone.0223143.s006.pdf]

S2 Table A. Percentage of each modeling area composed of each of 10 RND classes/bins based on each modeling regions' final model.

| RND Bin | Modeling Area | | | | | | | | | | | |
|---|---|---|---|---|---|---|---|---|---|---|---|---|
| | California Foothills | Central Basin and Range | Chihuahuan Desert | Columbia Plateau | Forested Montane | Intermontane Basins and Valleys | Northern Great Basin | Northwestern Plains | Southwestern Deserts | Southwestern Plains | Southwestern Plateaus | Wyoming Basin |
| 0.0-0.1 | 21.77 | 58.79 | 94.97 | 74.83 | 50.63 | 43.13 | 61.95 | 28.51 | 71.48 | 64.09 | 78.51 | 11.86 |
| 0.1-0.2 | 21.88 | 17.86 | 1.87 | 8.75 | 21.95 | 25.84 | 13.34 | 26.96 | 9.68 | 20.76 | 8.70 | 26.58 |
| 0.2-0.3 | 19.16 | 9.03 | 1.01 | 4.41 | 11.20 | 13.30 | 7.70 | 17.35 | 5.79 | 5.89 | 4.46 | 26.57 |
| 0.3-0.4 | 14.70 | 5.38 | 0.68 | 3.31 | 6.50 | 7.46 | 5.59 | 11.36 | 4.15 | 3.10 | 2.89 | 17.03 |
| 0.4-0.5 | 9.67 | 3.48 | 0.50 | 2.76 | 3.98 | 4.38 | 4.35 | 7.17 | 3.18 | 2.07 | 1.98 | 8.35 |
| 0.5-0.6 | 6.07 | 2.35 | 0.37 | 2.28 | 2.52 | 2.64 | 3.21 | 4.28 | 2.42 | 1.49 | 1.35 | 3.93 |
| 0.6-0.7 | 3.76 | 1.57 | 0.29 | 1.81 | 1.60 | 1.61 | 2.13 | 2.40 | 1.74 | 1.09 | 0.99 | 2.49 |
| 0.7-0.8 | 2.08 | 0.97 | 0.20 | 1.32 | 0.97 | 0.96 | 1.21 | 1.23 | 1.04 | 0.80 | 0.72 | 1.96 |
| 0.8-0.9 | 0.81 | 0.47 | 0.12 | 0.52 | 0.51 | 0.50 | 0.49 | 0.57 | 0.44 | 0.54 | 0.36 | 1.13 |
| 0.9-1.0 | 0.09 | 0.10 | 0.02 | 0.02 | 0.14 | 0.17 | 0.04 | 0.17 | 0.08 | 0.18 | 0.04 | 0.11 |

S2 Table B. Percentage of each modeling region composed of each of 10 RND classes/bins based on each modeling regions' final model.

| RND Bin | Modeling Region | | | | | | | | | | | |
|---|---|---|---|---|---|---|---|---|---|---|---|---|
| | California Foothills | Central Basin and Range | Chihuahuan Desert | Columbia Plateau | Forested Montane | Intermontane Basins and Valleys | Northern Great Basin | Northwestern Plains | Southwestern Deserts | Southwestern Plains | Southwestern Plateaus | Wyoming Basin |
| 0.0-0.1 | 24.59 | 60.30 | 97.24 | 82.68 | 55.05 | 44.26 | 65.45 | 36.58 | 77.42 | 77.14 | 77.92 | 12.43 |
| 0.1-0.2 | 22.74 | 17.50 | 1.07 | 6.41 | 21.28 | 25.85 | 12.64 | 27.71 | 7.81 | 15.19 | 8.95 | 26.40 |
| 0.2-0.3 | 19.37 | 8.62 | 0.55 | 3.20 | 10.53 | 13.02 | 7.14 | 15.56 | 4.60 | 3.43 | 4.53 | 26.23 |
| 0.3-0.4 | 13.98 | 5.14 | 0.36 | 2.28 | 5.72 | 7.21 | 5.02 | 9.00 | 3.26 | 1.60 | 2.92 | 17.22 |
| 0.4-0.5 | 8.56 | 3.35 | 0.27 | 1.81 | 3.27 | 4.19 | 3.79 | 5.22 | 2.46 | 0.98 | 1.98 | 8.40 |
| 0.5-0.6 | 5.21 | 2.25 | 0.19 | 1.46 | 1.93 | 2.51 | 2.75 | 2.96 | 1.86 | 0.65 | 1.37 | 3.90 |
| 0.6-0.7 | 3.16 | 1.47 | 0.15 | 1.11 | 1.16 | 1.50 | 1.80 | 1.62 | 1.33 | 0.44 | 1.07 | 2.45 |
| 0.7-0.8 | 1.70 | 0.88 | 0.10 | 0.76 | 0.67 | 0.87 | 1.00 | 0.83 | 0.83 | 0.31 | 0.83 | 1.86 |
| 0.8-0.9 | 0.62 | 0.41 | 0.07 | 0.29 | 0.32 | 0.44 | 0.39 | 0.39 | 0.39 | 0.20 | 0.40 | 1.03 |
| 0.9-1.0 | 0.07 | 0.09 | 0.01 | 0.01 | 0.07 | 0.14 | 0.03 | 0.12 | 0.06 | 0.07 | 0.05 | 0.09 |
