# Supplementary material for: Modeling spatial variation in density of golden eagle nest sites in the western United States
Source: PLoS One. 2019 Sep 30;14(9):e0223143. doi: 10.1371/journal.pone.0223143 (PMC6768475; doi:10.1371/journal.pone.0223143)
Supplement: S3 Table — (PDF) [file pone.0223143.s007.pdf]

S3 Table. Acknowledgment of individuals and institutions who contributed data and valuable input to this project.

Contact Name	Contact Organization
Shannon Albeke	University of Wyoming
Chad Anderson	National Park Service, Bryce Canyon National Park
Tara Anderson	Bureau of Land Managament, Shoshone Field Office
Mark Balman	BirdLife International
Lisa Baril	National Park Service, Yellowstone National Park
Joe Barnes	Nevada Department of Wildlife
Carie Battisone	California Department of Fish and Wildlife
Greg Beatty	US Fish and Wildlife Service, Western Golden Eagle Team
Dale Becker	The Confederated Salish and Kootenai Tribes
Bryan Bedrosian	Teton Raptor Center
Allison Begley	Montana Fish, Wildlife, and Parks
Katie Benzel	Bureau of Land Managament, Dillon Field Office
David Bittner	Wildlife Research Institute Inc
Gretchen Blatz	Washington Department of Fish and Wildlife
Pete Bloom	Bloom Biological, Inc
Clint Boal	Texas Tech University, USGS Texas Cooperative Fish and Wildlife Research Unit
John Boone	Great Basin Bird Observatory
Sandra Borthwick	National Park Service, Capitol Reef National Park
Nick Brown	NA
Kerry Burns	US Forest Service, Black Hills National Forest
Melissa Burns	US Fish and Wildlife Service, Utah Ecological Services Field Office
Tim Byer	US Forest Service, Thunder Basin National Grassland
Jason Carlisle	Western EcoSystems Technology, Inc
Jay Carlisle	Intermountain Bird Observatory
John Carlson	Bureau of Land Managament, Montana/Dakotas State Office
Dan Casey	Northern Great Plains Joint Venture
Ross Crandall	Craighead Beringia South
Miguel Cruz	Pronatura Mexico
Trish Cutler	US Department of Defense, White Sands Missile Range
Nathan Darnall	US Fish and Wildlife Service, Wyoming Ecological Services Field Office
Keith Day	Utah Division of Wildlife Resources
Jim Dick	US Fish and Wildlife Service, Western Golden Eagle Team
Thomas Dietsch	US Fish and Wildlife Service, R8 Carlsbad Field Office
Tom Dilts	University of Nevada, Reno
Chris Dirk	North Dakota Parks and Recreation Department
Daniel Driscoll	American Eagle Research Institute
Jamey Driscoll	Arizona Game and Fish Department
Kathy Duttenhefner	North Dakota Natural History Inventory
Steve Dyke	North Dakota Game and Fish Department
April Estep	Colorado Parks and Wildlife
Tammy Fletcher	US Forest Service, Caribou National Forest
Thomas Flowers	Private Researcher
Gail Garber	Hawks Aloft, Inc
Rick Gerhardt	SageScience
Sarah Haas	National Park Service, Bryce Canyon National Park
Derek Hall	National Security Tehcnologies, LLC
Eric Hallingstad	Western EcoSystems Technology, Inc
Al Harmata	Montana State University
Rick Harness	EDM International
Destin Harrell	Bureau of Land Managament, Cody Field Office
Gerald Hayes	Washington Department of Fish and Wildlife
Gjon Hazard	US Fish and Wildlife Service, Western Golden Eagle Team
Casey Heimerl	South Dakota Department of Game, Fish, and Parks
Highlands Ranch Community Association	Highlands Ranch Community Association
Mary Jo Hill	US Fish and Wildlife Service, Charles M Russell National Wildlife Refuge
Dylan Hopkins	Utah State University, Department of Biology - Sullivan Lab
Gregory Holm	National Park Service, Grand Canyon National Park
Grainger Hunt	The Peregrine Fund

Rich Inman	US Geological Survey, Las Vegas Field Office
Frank Isaacs	Oregon Eagle Foundation
Kenneth Jacobson	Arizona Game and Fish Department
Mackenzie Jeffries	Nevada Department of Wildlife
Aran Johnson	Southern Ute Nation
Sandy Johnson	North Dakota Game and Fish Department
Kate Keiser	California Department of Fish and Wildlife
Kent Keller	Private Researcher
John Kendall	Bureau of Land Managament, Farmington Field Office
Christy Klinger	Nevada Department of Wildlife
David Klute	Colorado Parks and Wildlife
Sonya Knetter	Idaho Department of Fish and Game
Robbie Knight	US Army, Dugway Proving ground
Michael Kochert	US Geological Survey, Forest & Rangeland Ecosystem Science Center
Patrick Kolar	US Geological Survey, Forest & Rangeland Ecosystem Science Center
Patty Kruger	USDA Forest Service, Pacific Southwest Region
Rory Lamp	Nevada Department of Wildlife
Russ Lawrence	US Air Force, Hill AFB, UTTR
David Leal	US Fish and Wildlife Service, Western Golden Eagle Team
Karen Lee	US Forest Service, Rocky Mountain Regional Office
Doug Leslie	ICF International, Inc
Sarah Lindsey	Utah Natural Heritage Program
Jim Lindstrom	US Fish and Wildlife Service, Wyoming Ecological Services Field Office
Mike Lockhart	Wildlands Photography and Bio-Consulting
Julie Luetzelschwab	US Forest Service, Sante Fe National Forest
Robyn MacDuff	Raptor Inventory Nest Surveys
Erin Madson	US Fish and Wildlife Service Wyoming Ecological Services Field Office
Paige Maskill	US Fish and Wildlife Service Montana Ecological Services Field Office
Randy Matchett	US Fish and Wildlife Service, Charles M Russell National Wildlife Refuge
Kyle McCarty	Arizona Game and Fish Department
Trent McDonald	Western EcoSystems Technology, Inc
Gwyn McKee	Thunderbird Widllife Consulting, Inc
Tony McKinney	US Fish and Wildlife Service, R8 Carlsbad Field Office
Martin Miller	Montana Natural Heritage Program
David Moen	Nez Perce Wildlife Division
Libby Mojica	EDM International
Colleen Moulton	Idaho Department of Fish and Game
Robert Murphy	US Fish and Wildlife Service, R2 Division of Migratory Birds
Robin Naeve	Bureau of Land Managament, Utah State Office
Randy Naugle	US Fish and Wildlife Service, Carlsbad Field Office
Janice Naylor	US Forest Service, Pawnee National Grassland
Justin Neighbor	Red Cliffs Desert Reserve
Chris Nicolai	US Fish and Wildlife Service, Reno Field Office
Russ Norvell	Utah Division of Wildlife Resources
Brian Novosak	Bureau of Land Management, Nevada
Bob Oakleaf	Wyoming Game and Fish Department (WGFD)
Wayne Peay	Raptor Inventory Nest Surveys (RINS)
Kristen Philbrook	US Forest Service, Pawnee National Grassland
Lonnie Pilkington	National Park Service, Glen Canyon National Recreation Area
Marty Piorkowski	Arizona Game and Fish Department
Steve Plunkett	US Forest Service, Southwestern Regional Office
Katie Powell	US Fish and Wildlife Service, Western Golden Eagle Team
Fritz Prellwitz	Bureau of Land Managament, Malta Field Office
Chuck Preston	Buffalo Bill Center of the West, Draper Natural History Museum
Louis Provencher	The Nature Conservancy
Drew Rayburn	Jefferson County Open Space
Larry Reigel	US Fish and Wildlife Service, Oregon Fish and Wildlife Office
David Rivers	Native Range Capture Services
Dan Roddy	National Park Service, Wind Cave National Park

Carl Rudeen	Mountain Home Air Force Base
Matt Rustand	Bureau of Land Managament, Royal Gorge Field Office
Robert Sacco	Colorado Parks and Wildlife
Amanda Schluter	Hawks Aloft, Inc
Rachel Simpson	Nebraska Game and Parks Commission, Nebraska Natural Heritage Program
David Skinner	US Forest Service, Sawtooth National Forest
Steve Slater	Hawkwatch International
Brian Smith	US Fish and Wildlife Service, R6 Division of Migratory Birds
Chad Smith	Navajo Nation
Doug Smith	National Park Service, Yellowstone National Park
Jeff Smith	HT Harvey and Associates
Kristina Smucker	Montana Fish, Wildlife, and Parks
Scott Somershoe	US Fish and Wildlife Service, R6 Division of Migratory Birds
Rob Spaul	Boise State University
John Spence	National Park Service, Glen Canyon National Recreation Area
Darci Stafford	Bureau of Land Managament, Buffalo Field Office
Dale Stahlecker	Eagle Environmental, Inc
John Stephenson	National Park Service, Grand Teton National Park
Janice Stroud-Settles	National Park Service, Grand Canyon National Park
Brian Sullivan	Cornell University, eBird
Jason Sutter	US Bureau of Land Management Idaho State Office
Trish Sweanor	US Fish and Wildlife Service, Wyoming Ecological Services Field Office
Bruce Thompson	Washington Department of Fish and Wildlife
Cris Tomlinson	Nevada Department of Wildlife
David Topolewski	US Forest Service, Rio Grande National Forest
Nick Van Lanen	Rocky Mountain Bird Observatory
Chet VanDellen	Nevada Department of Wildlife
Chris Vennum	Colorado State University
Joe Vieira	Bureau of Land Managament, Renewable Energy Office
Zach Wallace	Wyoming Natural Diversity Database
Arden Warm	US Forest Service, Dakota Prairie Grasslands
Jim Watson	Washington Department of Fish and Wildlife
Peter Weisberg	University of Nevada, Reno
Bonnie Weller	Nevada Department of Wildlife
Nathaniel West	Bureau of Land Managament, Tres Rios Field Office
Paul Whitefield	National Park Service, Walnut Canyon National Monument
Lynn Wickersham	Fort Lewis College, San Juan Institute of Natural and Cultural Resources
David Wiens	US Geological Survey, Forest & Rangeland Ecosystem Science Center
Gary Williams	US Fish and Wildlife Service, Western Golden Eagle Team
Don Wolfe	University of Oklahoma, GM Sutton Avian Research Center
Simon Wray	Oregon Dept of Fish and Wildlife
Dave Wrobleski	US Forest Service, Lolo National Forest
Mike Yates	BioResource Consultants
